# Supplementary material for: Reductive Activity and Mechanism of Hypoxia- Targeted AGT Inhibitors: An Experimental and Theoretical Investigation
Source: Int J Mol Sci. 2019 Dec 13;20(24):6308. doi: 10.3390/ijms20246308 (PMC6941096; doi:10.3390/ijms20246308)
Supplement: Supplementary file 1 [file ijms-20-06308-s001.pdf]

Supplementary Materials

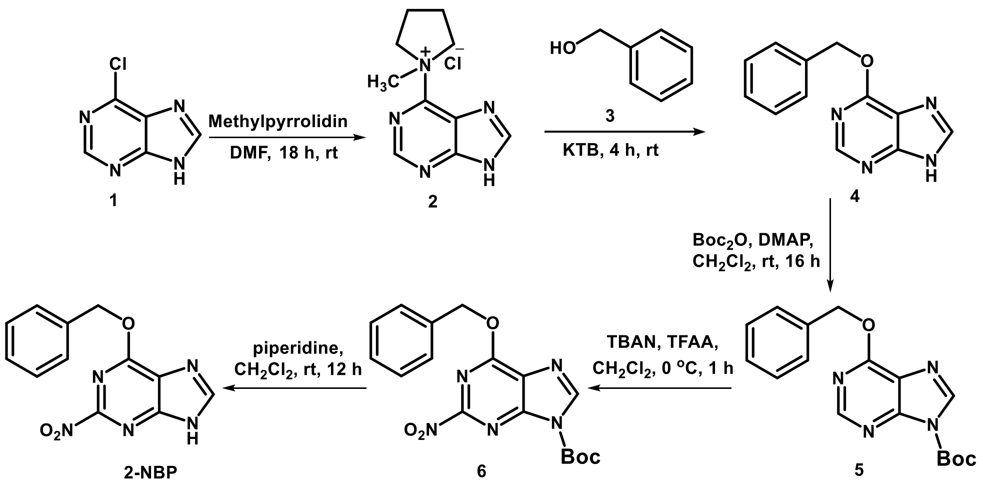

Figure S1. Synthesis of 2-NBP

1-methyl-1-(9*H*-purin-6-yl) pyrrolidin-1-ium chloride (compound 2)

To a solution of compound **1** (909 mg, 5.9 mmol) in DMF (40 mL) 1-methylpyrrolidine (1.4 mL) was added and the mixture was stirred at room temperature for 18 h. After completion of the reaction, the obtained crystals were filtrated and were recrystallized by ether, which gave compound **2** as a white solid (1041 mg, 5.1 mmol, yield 86%). ^{1}H-NMR (400 MHz, DMSO-d_6) δ: 1.754-1.738 (s, 3H, -CH_3), 4.131-4.157 (t, J=5.6 Hz, 4H, -CH_2-CH_2-), 4.684-4.709 (t, J=5.6 Hz, 4H, -CH_2-N^+(CH_3)-CH_2-), 8.175 (s, 1H, -N-*CH*-NH-), 8.934 (s, 1H, -N-*CH*-N-), 9.025 (s, 1H, -NH-) ppm. IR (KBr): 3437, 3039, 2912, 1612, 1323 cm^{-1}. HRMS (ESI), *m*/*z* calcd for $C_{10}H_{14}ClN_5$ $[M-Cl]^+$ 204.1244, found 204.1249.

6-(benzyloxy)-9*H*-purine (compound 4)

To a solution of compound **3** (374 μL, 3.4 mmol) in DMF (7 mL) potassium tert-butoxide (930 mg, 8.3 mmol) and compound **2** (430 mg, 2.1 mmol) were added and the mixture was stirred at room temperature for 4 h under nitrogen protection. After completion of the reaction, 14 mL deionized water containing 440 μL glacial acetic acid was added to the mixture. Then the mixture was extracted with 50 mL saturated NH_4Cl and 50 mL ethyl acetate solution, the organic phase was collected and dried over anhydrous sodium sulfate overnight. The solvent was removed by vacuum, and the residue was purified by column chromatography using methanol/CH_2Cl_2 (1/50~1/10), which gave compound **4** as a white solid (597 mg, 2.6 mmol, yield 77.8%). ^{1}H-NMR (400 MHz, DMSO-d_6) δ: 5.620 (s, 2H, -O-CH_2-), 7.356-7.428 (m, 5H, Ar), 8.517 (s, 1H, -N-*CH*-NH-), 8.354(s, 1H, -N-*CH*-N-), 13.430 (s, 1H, -NH-) ppm. IR (KBr): 3434, 3133, 2962, 1608, 1471, 1212 cm^{-1}. HRMS (ESI), *m*/*z* calcd for $C_{12}H_{10}N_4O$ $[M+H]^+$ 227.0927, found 227.0928.

tert-butyl 6-(benzyloxy)-9*H*-purine-9-carboxylate (compound 5)

To a solution of compound **4** (678 mg, 3 mmol) in CH_2Cl_2 (15 mL) di-tert-butyl dicarbonate (933 mg, 4.3 mmol) and dimethylaminopyridine (39 mg, 0.3 mmol) were added and the mixture was stirred at

room temperature for 16h. After completion of the reaction, the solvent was removed by vacuum, and the residue was purified by column chromatography using petroleum ether/ethyl acetate (1/1), which gave compound **5** as a white solid (734 mg, 2.25 mmol, yield 75%). ^{1}H-NMR (400 MHz, $CHCl_3$) δ: 1.716 (s, 9H, -$(CH_3)_3$), 5.696 (s, 2H, -O-CH_2-Ar), 7.335-7.399 (m, 5H, Ar), 8.741 (s, 1H, -N-*CH*-N-), 8.377 (s, 1H, -N-*CH*-N-COO-) ppm. IR (KBr): 3434, 3133, 2962.54, 1608, 1471, 1212 cm^{-1}. HRMS (ESI), *m/z* calcd for $C_{17}H_{18}N_4O_3$ $[M+H]^+$ 327.1452, found 327.1457.

tert-butyl 6-(benzyloxy)-2-nitro-9*H*-purine-9-carboxylate (compound 6)

To a solution of compound **5** (652 mg, 2 mmol) in CH_2Cl_2 (15 mL) tetrabutylammonium nitrate (852 mg, 2.8 mmol) and dichloromethane (0.5 mL) containing trifluoroacetic anhydride (366 μL, 1.2 mmol) were added dropwisely for 1-2 min and the mixture was stirred for 1h under ice bath. After completion of the reaction, the mixture was extracted with 30 mL saturated $NaHCO_3$ and 30 mL ether solution, the organic phase was collected and was dried over anhydrous sodium sulfate overnight. The solvent removed by vacuum, and then was added cold methanol, which gave compound **6** as a yellow solid (586 mg, 1.6 mmol, yield 80%). ^{1}H-NMR (400 MHz, $CHCl_3$) δ: 1.740 (s, 9H, -$(CH_3)_3$), 5.785 (s, 2H, -CH_2-), 7.369-7.429 (m, 5H, Ar), 8.606(s, 1H, -CH-) ppm. IR (KBr): 3435, 3122, 2982, 1762, 1609, 1558, 1308 cm^{-1}. HRMS (ESI), *m/z* calcd for $C_{17}H_{17}N_5O_5$ $[M+H]^+$ 372.1302, found 372.1308.

6-(benzyloxy)-2-nitro-9*H*-purine (2-NBP)

To a solution of compound **6** (371 mg, 1 mmol) in CH_2Cl_2 (10 mL) piperidine (100 μL, 1 mmol) was added and the mixture was stirred at room temperature for 12 h. After completion of the reaction, the solvent was removed by vacuum, and then was added ethyl acetate, which gave **2-NBP** as a yellow solid (81 mg, 0.3 mmol, yield 30%). ^{1}H-NMR (400 MHz, DMSO) δ: 5.617 (s, 2H, -CH_2-), 7.370-7.438 (m, 5H, Ar), 7.581 (s, 1H, -CH-), 8.442 (s, 1H, -NH-) ppm. IR (KBr): 3435, 3118, 2950, 1596, 1548, 1356 cm^{-1}. HRMS (ESI), *m/z* calcd for $C_{12}H_9N_5O_3$ $[M+H]^+$ 272.0778, found 272.0770.

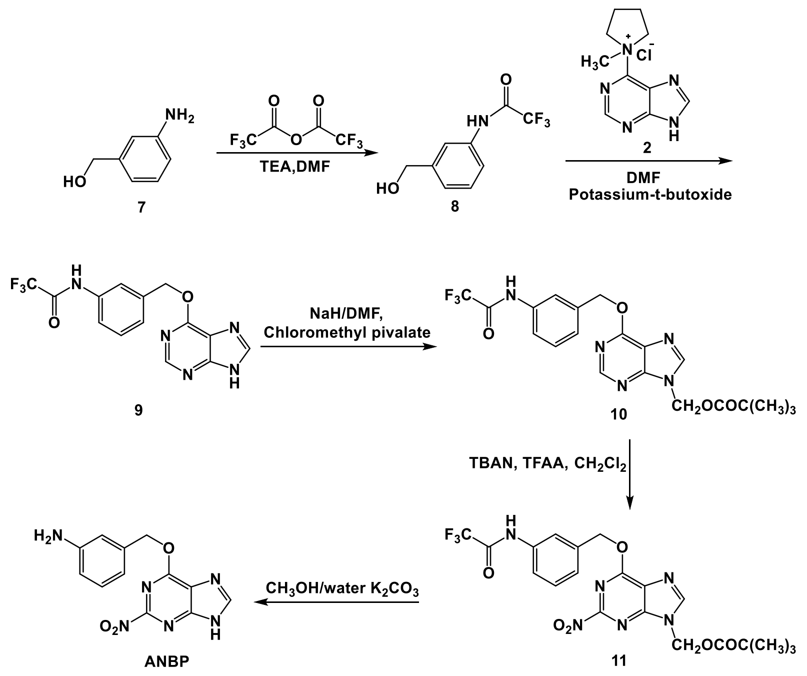

Figure S2. Synthesis of ANBP

2,2,2-trifluoro-*N*-(3-(hydroxymethyl)phenyl)acetamide (compound 8)

To a solution of compound **7** (369 mg, 3 mmol) in tetrahydrofuran (10 mL) triethylamine (484 μL, 3.5 mmol) and trifluoroacetic anhydride (493 μL, 3.5 mmol) were added dropwisely at 0 ° C for 30 minutes and the mixture was stirred at room temperature for 3 hours. After completion of the reaction, the solution was extracted with ethyl acetate (30 mL) and deionized water (30 mL). The organic phase was collected and was dried over anhydrous sodium sulfate overnight. The solvent was removed by vacuum, and the residue was purified by column chromatography using petroleum ether/ethyl acetate (1/1~1/1), which gave compound **8** as a white solid (483 mg, 2.21 mmol, yield 74%). ^{1}H-NMR (400 MHz, DMSO-d_6) δ: 4.505(s, 2H, -CH_2-), 7.152 (s, 1H, -OH), 7.170-7.656(m, 4H, Ar), 11.212 (s, 1H, -NH-) ppm. IR (KBr): 3473, 3263, 3095, 2935, 1699, 1574, 1465, 1019, 950 cm^{-1}. HRMS (ESI), *m*/*z* calcd for $C_9H_8F_3NO_2$ $[M+H]^+$ 220.0580, found 220.0585 .

***N*-(3-(((9*H*-purin-6-yl)oxy)methyl)phenyl)-2,2,2-trifluoroacetamide (compound 9)**

To a solution of compound **8** (556 mg, 2.54 mmol) in DMF (15 mL) potassium tert-butoxide (599 mg, 5.33 mmol) and compound 2 (300 mg, 1.18 mol) were added and the mixture was stirred for 4 h at room temperature under nitrogen potection. After completion of the reaction, to the mixture 7 mL deionized water containing 200 μL glacial acetic acid was added. The mixture was extracted with NH_4Cl (50 mL) and ethyl acetate (50 mL). The organic phase was collected and was dried over anhydrous sodium sulfate overnight. The solvent was removed by vacuum, and the residue was purified by column chromatography using methanol / dichloromethane (1/50~1/10), which gave compound **9** as a white solid (676 mg, 2.01 mmol, yield 79%). ^{1}H-NMR (400 MHz, DMSO-d_6) δ: 4.512 (s, 2H, -CH_2-), 5.258 (s, 1H, -CO-*NH*-Ar-), 7.151-7.349 (m, 4H, -Ar-), 7.369 (s, 1H, -N-*CH*-NH-), 7.655 (s, 1H, -N-*CH*-N-), 11.212 (s, 1H, -C-*NH*-C-) ppm. IR (KBr): 3474, 3227, 3095, 2935, 1699, 1599, 1465, 1003, 879 cm^{-1}. HRMS (ESI), *m*/*z* calcd for $C_{14}H_{10}F_3N_5O_2$ $[M+H]^+$ 338.0859, found 338.0854.

(6-((3-(2,2,2-trifluoroacetamido)benzyl)oxy)-9*H*-purin-9-yl)methyl pivalate (compound 10)

To a solution of compound **9** (711 mg, 2.1 mmol) in DMF (12 mL) NaH (43 mg, 1.8 mmol) was added and the mixture was stirred at 0°C for 30 minutes. Then, chloromethyl pivalate (0.432 mL. 3 mmol) was added and the mixture was stirred at room temperature for 1 h. After completion of the reaction, acetic acid was added to the mixture to adjust the pH to 7.0followed by extraction with ethyl acetate (50 mL) and deionized water (50 mL). The organic phase was collected and was dried over anhydrous sodium sulfate overnight. The solvent was removed by vacuum, and the residue was purified by column chromatography using ethyl acetate/petroleum ether (1/1), which gave compound **10** as a white solid (540 mg, 1.2 mmol, yield 57%). ^{1}H-NMR (400 MHz, DMSO-d_6) δ: 1.184 (s, 9H, -$(CH_3)_3$), 6.197 (s, 2H, -CO-CH_2-N-), 5.633 (s, 2H, -O-CH_2-Ar-), 7.279-7.685 (m, 4H, Ar), 8.207 (s, 1H, -N-*CH*-N-), 8.615 (s, 1H, -N-*CH*-NCOCH$_2$Ar-), 8.826 (s, 1H, -NH-) ppm. IR (KBr): 3385, 2924, 2853, 1689, 1590, 1467, 1364, 1272, 890, 794 cm^{-1}. HRMS (ESI), *m/z* calcd for $C_{20}H_{20}F_3N_5O_4$ [M+H]$^+$ 452.1540, found 452.1546.

(2-nitro-6-((3-(2,2,2-trifluoroacetamido)benzyl)oxy)-9*H*-purin-9-yl)methyl pivalate (compound 11)

To a solution of compound **10** (677 mg, 1.5 mmol) in CH_2Cl_2 (20 mL) tetrabutylammonium nitrate (685 mg, 2.24 mmol) and trifluoroacetic anhydride (0.289 mL, 2.0 mmol) were added dropwisely and the mixture was stirred at 0°C for 2h. After completion of the reaction, the mixture was extracted with ether (50 mL) and saturated $NaHCO_3$ (50 mL). The organic phase was collected and dried over anhydrous sodium sulfate overnight. The solvent was removed by vacuum, and the residue was added ice methanol (4.5 mL), which gave compound **11** (549 mg, 1.11 mmol, yield 74%). ^{1}H-NMR (400 MHz, DMSO-d_6) δ: 1.184 (s, 9H, -$(CH_3)_3$), 6.197 (s, 2H, -CO-CH_2-N-), 5.633 (s, 2H, -O-CH_2-Ar-), 7.279-7.685 (m, 4H, Ar), 8.615 (s, 1H, -CH-), 8.826 (s, 1H, -NH-) ppm. IR (KBr): 3387, 2824, 2803, 1679, 1587, 1437, 1354, 1252, 890, 794, 740, 719 cm^{-1}. HRMS (ESI), *m/z* calcd for $C_{20}H_{19}F_3N_6O_6$ [M+H]$^+$ 497.1391, found 497.1393.

3-(((2-nitro-9*H*-purin-6-yl)oxy)methyl)aniline (ANBP)

To a solution of compound **11** (723 mg, 1.5 mmol) in methanol (34 mL) and deionized water (2 mL) anhydrous potassium carbonate (1105 mg, 8 mmol) was added and the mixture was refluxed at 68°C for 2h. After completion of the reaction, the solvent was removed by vacuum, and the residue was purified by column chromatography using methanol/dichloromethane/triethylamine (1/5/0.05), which gave **ANBP** as a yellow solid (322 mg, 1.12 mmol, yield 75%). ^{1}H-NMR (400 MHz, DMSO-d_6) δ: 4.512 (s, 2H, -O-CH_2-), 5.258 (s, 2H, -NH_2), 7.151-7.369 (m, 4H, Ar), 7.655 (s, 1H, -N-*CH*-N-), 11.212 (s, 1H, -NH-) ppm. IR (KBr): 3435, 1619, 1468, 1364, 1204, 1064, 791 cm^{-1}. HRMS (ESI), *m/z* calcd for $C_{12}H_{10}N_6O_3$ [M+H]$^+$ 287.0887, found 287.0881.

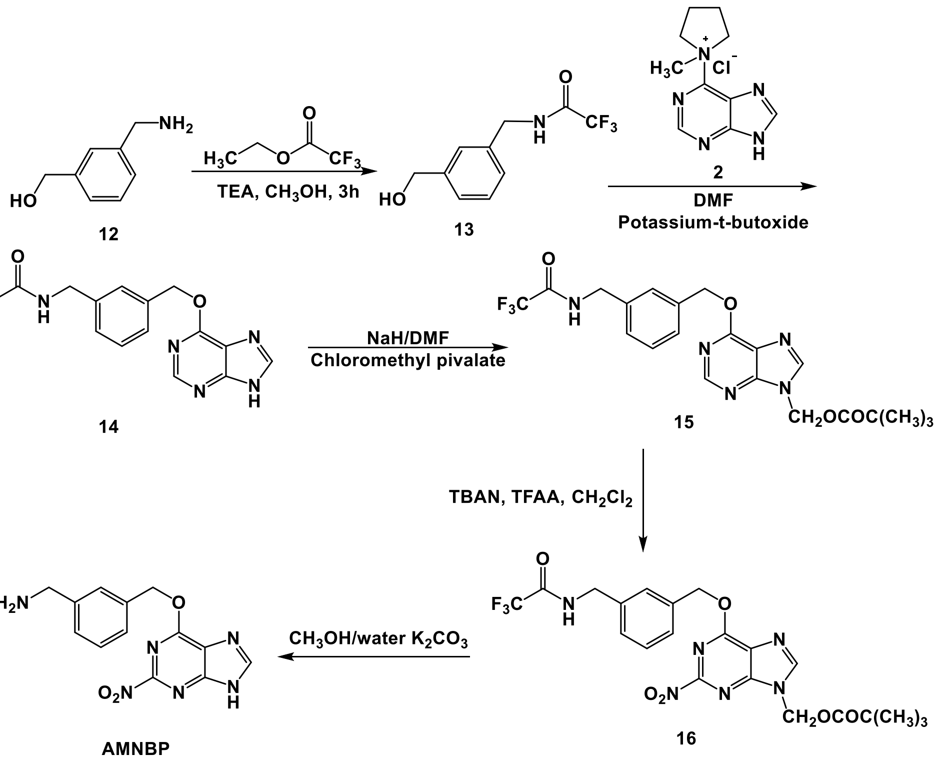

Figure S3. Synthesis of AMNBP

2,2,2-trifluoro-*N*-(3-(hydroxymethyl)benzyl)acetamide (compound 13)

To a solution of compound **12** (430 mg, 3.1 mmol) in anhydrous methanol (6 mL) triethylamine (559 μL, 4.1 mmol) and trifluoroacetate (500 μL, 4.1 mmol) were added dropwisely and the mixture was stirred at room temperature for 3h. After completion of the reaction, the mixture was extracted with ethyl acetate (50 mL) and deionized water (50 mL). The organic phase was collected and was dried over anhydrous sodium sulfate overnight. The solvent was removed by vacuum, and the residue was purified by column chromatography using petroleum ether/ethyl acetate (1/1), which gave compound **13** as a white solid (592 mg, 2.54 mmol, yield 82%). ^{1}H-NMR (400 MHz, DMSO-d_6) δ: 4.544-4.559 (d, *J*=6.0 Hz, 2H, -Ar-*CH*$_2$-NH-), 4.724 (s, 2H, -Ar-*CH*$_2$-OH), 6.635 (s, 1H, -NH-), 7.279 (s, 1H, -OH), 7.322-7.394 (m, 4H, Ar) ppm. IR (KBr): 3287, 3106, 2935, 1703, 1553, 1178, 1047, 890 cm^{-1}. HRMS (ESI), *m*/*z* calcd for $C_{10}H_{10}F_3NO_2$ $[M+H]^+$ 234.0736, found 234.0738.

***N*-(3-(((9*H*-purin-6-yl)oxy)methyl)benzyl)-2,2,2-trifluoroacetamide (compound 14)**

To a solution of compound **13** (349 mg, 1.5 mmol) in DMF (8 mL) potassium tert-butoxide (337 mg, 3 mmol) and compound **2** (178 mg, 0.7 mmol) were added and the mixture was stirred under argon for 4 h at room temperature. After completion of the reaction, the mixture was extracted with NH_4Cl (30 mL) and ethyl acetate (30 mL). The organic phase was collected and was dried over anhydrous sodium sulfate overnight. The solvent was removed by vacuum, and the residue was purified by column chromatography using methanol / dichloromethane (1/50~1/10), which gave compound **14** as a white solid (384 mg, 1.09 mmol, yield 73%). ^{1}H-NMR (400 MHz, DMSO-d_6) δ: 4.405-4.420 (d, *J*=6.0 Hz, 2H, -NH-*CH*$_2$-Ar-), 5.626 (s, 2H, -Ar-CH$_2$-O-), 7.277-7.435 (m, 4H, Ar), 7.955 (s, 1H, -CO-*NH*-CH$_2$-), 8.346(s,

1H, -NH-*CH*-N-), 8.498(s, 1H, -N-*CH*-N-), 13.420(s, 1H, -C-*NH*-CH-) ppm. IR (KBr): 3418, 3212, 2905, 1715, 1591, 1201, 1179, 945cm^{-1}. HRMS (ESI), *m/z* calcd for $C_{15}H_{12}F_3N_5O_2$ $[M+H]^+$ 352.1016, found 352.1012.

(6-((3-(2,2,2-trifluoroacetaminomethyl)benzyl)oxy) -9*H*-purin-9-yl)methyl pivalate (compound 15)

To a solution of compound **14** (527 mg, 1.5 mmol) in DMF (12 mL) HNa (48 mg, 2 mmol) was added and the mixture was stirred at 0°C for 30 min. Then, chloromethyl pivalate (0.432 mL. 3mmol) was added and the mixture was stirred at room temperature for 1 h. After completion of the reaction, acetic acid was added to adjust the pH to 7.0 followed by extraction with ethyl acetate (40 mL) and deionized water (40 mL). The organic phase was collected and was dried over anhydrous sodium sulfate overnight. The solvent was removed by vacuum, and the residue was purified by column chromatography using ethyl acetate/petroleum ether (1/1), which gave compound **15** as a white solid (453 mg, 0.98 mmol, yield 65%). ^{1}H-NMR (400 MHz, DMSO-d_6)δ:1.169 (s, 9H, -$(CH_3)_3$), 4.471-4.485 (d, *J*=5.6 Hz 2H, -Ar-*CH*$_2$-NH-), 5.635 (s, 2H, -CO-*CH*$_2$-N-), 6.177 (s, 2H, -O-*CH*$_2$-Ar-), 7.268-7.379 (m, 4H, Ar), 8.171 (s, 1H, -N-*CH*-N-), 7.431 (s, 1H, -N-*CH*-NCO-), 8.603 (s, 1H, -NH-) ppm.IR (KBr): 3220, 3052, 2932, 1731, 1601, 1458, 1178, 999 cm^{-1}. HRMS (ESI), *m/z* calcd for $C_{21}H_{22}F_3N_5O_4$ $[M+H]^+$ 466.1697, found 466.1690.

(2-nitro-6-((3-((2,2,2-trifluoroacetamido)methyl)benzyl)oxy)-9*H*-purin-9-yl)methyl pivalate (compound 16)

To a solution of compound **15** (699 mg, 1.5 mmol) in CH_2Cl_2 (20 mL) tetrabutylammonium nitrate (918 mg, 3 mmol) and dropwise trifluoroacetic anhydride (0.282 mL, 1.95 mmol) were added and the mixture was stirred at 0°C for 1.5 h. After completion of the reaction, the mixture was extracted with ether (30 mL) and saturated $NaHCO_3$ (30 mL). The organic phase was collected and was dried over anhydrous sodium sulfate overnight. The solvent was removed by vacuum, and the residue was added ice methanol (3 mL), which gave compound **16** (581 mg, 1.14 mmol, yield 76%). ^{1}H-NMR (400 MHz, DMSO-d_6) δ: 1.188(s, 9H, -$(CH_3)_3$), 4.567-4.581 (d, *J*=5.6 Hz, 2H, -Ar-*CH*$_2$-NH-), 5.765 (s, 2H, -CO-*CH*$_2$-N-), 6.231 (s, 2H, -O-*CH*$_2$-Ar-), 7.331-7.437 (m, 4H, Ar), 7.612 (t, *J*=7.6 Hz 1H, -NH-), 8.430 (s, 1H, -CH-) ppm. IR (KBr): 3449, 2959, 1631, 1468, 1365, 1273, 1202, 793 cm^{-1}. HRMS (ESI), *m/z* calcd for $C_{21}H_{21}F_3N_6O_6$ $[M+H]^+$ 511.1547, found 511.1542.

(3-(((2-nitro-9*H*-purin-6-yl)oxy)methyl)phenyl)methanamine (AMNBP)

To a solution of compound **16** (767 mg, 1.5 mmol) in methanol (34 mL) and deionized water (2 mL) anhydrous potassium carbonate (1107 mg, 7.3 mmol) was added and the mixture was refluxed at 68°C for 2 h. After completion of the reaction, the solvent was removed by vacuum, and the residue was purified by column chromatography using methanol / dichloromethane / triethylamine (1/5/0.05), which gave **AMNBP** as a red solid (342 mg, 1.14 mmol, yield 76%). ^{1}H-NMR (400 MHz, DMSO-d_6)δ: 4.405-4.420 (d, *J*=6.0 Hz, 2H, -Ar-*CH*$_2$-NH$_2$-), 5.626 (s, 2H, -O-*CH*$_2$-Ar-), 7.151-7.369 (m, 4H, Ar), 8.346-8.498 (s, 2H, -NH$_2$-), 10.005 (s, 1H, -CH-), 13.420 (s, 1H, -NH-) ppm. IR (KBr): 3385, 2957, 1732, 1592, 1551, 1468, 1365, 1161, 1045, 897, 702 cm^{-1}. HRMS (ESI), *m/z* calcd for $C_{13}H_{12}N_6O_6$ $[M+H]^+$ 301.1044, found 301.1041.

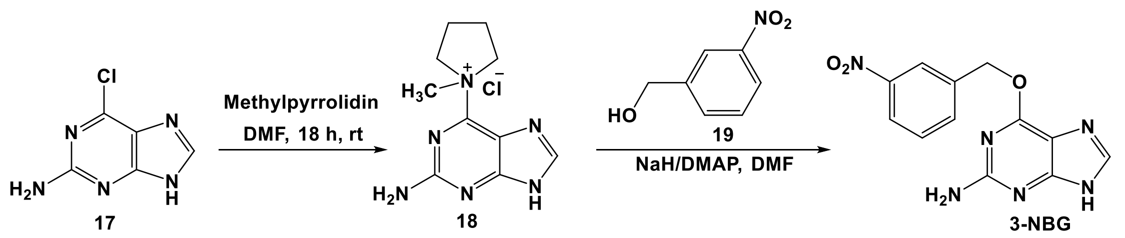

Figure S4. Synthesis of 3-NBG

1-(2-amino-9*H*-purin-6-yl)-1-methylpyrrolidin-1-ium chloride (compound 18)

To a solution of compound **17** (1 mg, 5.9 mmol) in DMF (50 mL) 1-methylpyrrolidine (1.4 mL, 13.2 mmol) was added and the mixture was stirred at room temperature for 19 h.. After completion of the reaction, 3 mL acetone was added to the mixture, which gave compound **18** as a white solid (1041 mg, 4.1 mmol, yield 70%). ^{1}H-NMR (400 MHz, DMSO-d_6) δ: 2.055-2.248 (t, *J*=2.5 Hz, 2H, -*CH*$_2$-CH$_2$-), 2.498-2.507 (t, *J*=3.6 Hz, 2H, -CH$_2$-*CH*$_2$-), 3.651 (s, 3H, -CH$_3$), 3.937-3.979 (s, 2H, -*CH*$_2$-N$^+$(CH$_3$)-CH$_2$-), 4.587-4.611 (d, *J*=9.6 Hz, 2H, -CH$_2$-N$^+$(CH$_3$)-*CH*$_2$-), 7.104 (s, 2H, -NH$_2$) 8.342 (s, 1H, -NH-) ppm. IR (KBr): 3437, 3039, 2912, 1612, 1323 cm^{-1}. HRMS (ESI), *m*/*z* calcd for $C_{10}H_{15}ClN_6$ [M+H]$^+$ 219.1353, found 219.1358.

6-((3-nitrobenzyl)oxy)-9*H*-purin-2-amine (3-NBG)

To a solution of compound **18** (538 mg, 2.46 mmol) in DMF (15 mL) NaH (163 mg, 6.79 mmol) , compound **19** (310 mg ,1.22 mmol) and dimethylaminopyridine (18 mg, 0.15mmol) were added and the mixture was stirred at room temperature for 5 h under nitrogen protection. After completion of the reaction, to the mixture 7 mL deionized water containing 220 μL glacial acetic acid was added. The mixture was extracted with 40 mL saturated NH_4Cl and 40 mL ethyl acetate, the organic phase was collected and dried over anhydrous sodium sulfate overnight. The solvent was removed by vacuum, and the residue was purified by column chromatography using methanol/CH_2Cl_2 (1/50~1/10), which gave **3-NBG** as a brown solid (232 mg, 0.81 mmol, yield 33 %). ^{1}H-NMR (400 MHz, DMSO-d_6)δ: 5.761 (s, 2H, -O-*CH*$_2$-), 6.629 (s, 2H, -NH$_2$), 8.160-8.336 (m, 4H, Ar), 9.568 (s, 1H, -CH-), 13.718 (s, 1H, -NH-) ppm. IR (KBr): 3400, 2924, 1529, 1403, 1141 cm^{-1}. HRMS (ESI), *m*/*z* calcd for $C_{12}H_{10}N_6O_3$ [M+H]$^+$ 287.0887, found 287.0889.

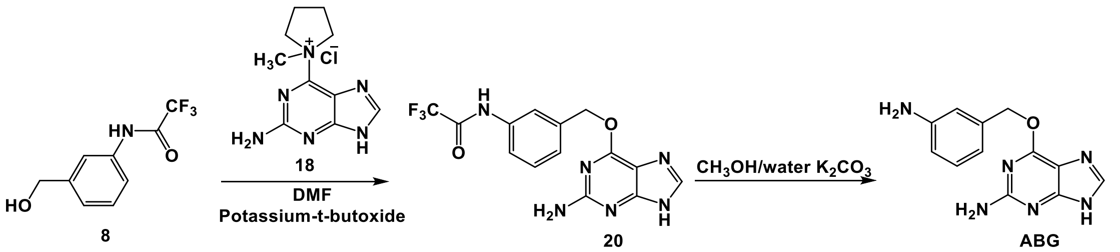

Figure S5. Synthesis of ABG

***N*-(3-(((2-amino-9*H*-purin-6-yl)oxy)methyl)phenyl)-2,2,2-trifluoroacetamide (compound 20)**

To a solution of compound **8** (556 mg, 2.54 mmol) in DMF (15 mL) potassium tert-butoxide (599 mg, 5.33 mmol) and compound 18 (300 mg, 1.37 mmol) were added and the mixture was stirred at room

temperature for 4 h under nitrogen protection. After completion of the reaction, to the mixture 7 mL deionized water containing 220 μL glacial acetic acid was added. The mixture was extracted with 40 mL saturated NH_4Cl and (40 mL) ethyl acetate. The organic phase was collected and was dried over anhydrous sodium sulfate overnight. The solvent was removed by vacuum, and the residue was purified by column chromatography using methanol/CH_2Cl_2 (1/50~1/10), which gave compound **20** as a white solid (327 mg, 0.93 mmol, yield 37%). ^{1}H-NMR (400 MHz, DMSO-d_6) δ: 5.633 (s, 2H, -O-*CH*$_2$-Ar-), 6.197 (s, 2H, -NH_2), 7.279-7.685 (m, 4H, Ar), 8.207 (s, 1H, -CO-*NH*-Ar-), 8.615 (s, 1H, -N-*CH*-N-), 12.425 (s, 1H, -NH-) ppm. IR (KBr): 3529, 3399, 3208, 3007, 1453, 1267, 979, 878 cm^{-1}. HRMS (ESI), *m*/*z* calcd for $C_{14}H_{11}F_3N_6O_2$ $[M+H]^+$ 353.0968, found 353.0967.

6-((3-aminobenzyl)oxy)-9*H*-purin-2-amine (ABG)

To a solution of compound **20** (320 mg, 0.91 mmol) in methanol (34 mL) and deionized water (2 mL) anhydrous potassium carbonate (656 mg, 4.75 mmol) was added and the mixture was refluxed at 68°C for 2h. After completion of the reaction, the solvent was removed by vacuum, and the residue was purified by column chromatography using methanol / dichloromethane / triethylamine (1/5/0.05), which gave **ABG** as a white solid (202 mg, 0.79 mmol, yield 87%). ^{1}H-NMR (400 MHz, DMSO-d_6) δ: 5.114 (s, 2H, -O-*CH*$_2$-Ar-), 5.332 (s, 2H, -C-*NH*$_2$), 6.275 (s, 2H, -Ar-*NH*$_2$), 6.584-7.031 (m, 4H, Ar), 7.807 (s, 1H, -CH-), 12.416 (s, 1H, -NH-) ppm. IR (KBr): 3651, 3198, 2986, 2800, 2679, 1689, 1588, 1347, 1202, 947, 825, 701 cm^{-1}.HRMS (ESI), *m*/*z* calcd for $C_{12}H_{12}N_6O$ $[M+H]^+$ 257.1145, found 257.1144.

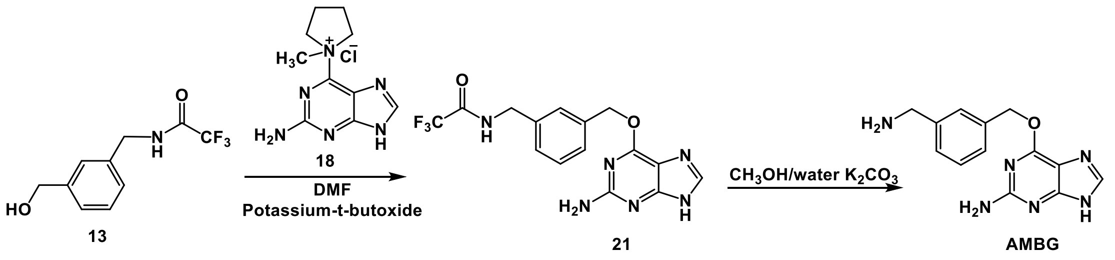

Figure S6. Synthesis of AMBG

***N*-(3-(((2-amino-9*H*-purin-6-yl)oxy)methyl)benzyl)-2,2,2-trifluoroacetamide (compound 21)**

To a solution of compound **13** (592 mg, 2.54 mmol) in DMF (15 mL) potassium tert-butoxide (599 mg, 5.33 mmol) and compound **18** (300 mg, 1.18 mmol) were added and the mixture was stirred under argon for 4 h at room temperature. After completion of the reaction, to the mixture 7 mL deionized water containing 220 μL glacial acetic acid was added. The solution was extracted with NH_4Cl (40 mL) and ethyl acetate (40 mL). The organic phase was collected and was dried over anhydrous sodium sulfate overnight. The solvent was removed by vacuum, and the residue was purified by column chromatography using methanol / dichloromethane (1/50~1/10), which gave compound **21** as a white solid (340 mg, 0.93 mmol, yield 37%). ^{1}H-NMR (400 MHz, DMSO-d_6) δ: 4.485-4.471 (d, *J*=5.6 Hz, 2H, -NH-*CH*$_2$-Ar-), 5.635 (s, 2H, -O-*CH*$_2$-), 6.177 (s, 2H, -NH_2), 7.268-7.431 (m, 4H, Ar), 8.171 (s, 1H, -CH), 8.603 (s, 1H, -CO-*NH*-CH_2-) 11.212 (s, 1H, -C-*NH*-C-) ppm. IR (KBr): 3363, 3240, 3073, 2856, 1629, 1587, 1492, 1420, 1183, 1053, 791, 758, 724 cm^{-1}.HRMS (ESI), *m*/*z* calcd for $C_{15}H_{12}F_3N_6O_2$ $[M+H]^+$ 367.1125, found 367.1123.

6-((3-(aminomethyl)benzyl)oxy)-9*H*-purin-2-amine (AMBG)

To a solution of compound **21** (333 mg, 0.91 mmol) in methanol (34 mL) and deionized water (2 mL) anhydrous potassium carbonate (656 mg, 4.75 mmol) was added and the mixture was refluxed at 70°C for 2h. After completion of the reaction, the solvent was removed by vacuum. And the residue was purified by column chromatography using methanol / dichloromethane / triethylamine (1/5/0.05), which gave **AMBG** as a white solid (213 mg, 0.79 mmol, yield 87%). ^{1}H-NMR (400 MHz, DMSO-d_6) δ: 4.412-4.490 (t, *J*=5.6 Hz, 2H, -NH$_2$-*CH$_2$*-Ar-), 5.485 (s, 2H, -O-*CH$_2$*-), 6.297 (s, 2H, -C-*NH$_2$*), 7.249-7.442 (m, 4H, Ar), 7.817 (s, 2H, -CH$_2$-*NH$_2$*), 10.035 (s, 1H, -CH-), 12.434 (s, 1H, -NH-) ppm. IR (KBr): 3313, 3186, 3005, 2780, 2556, 1632, 1503, 1400, 1228, 950, 792, 764, 632, 608 cm^{-1}.HRMS (ESI), *m*/*z* calcd for $C_{13}H_{14}N_6O$ $[M+H]^+$ 271.1301, found 271.1300.

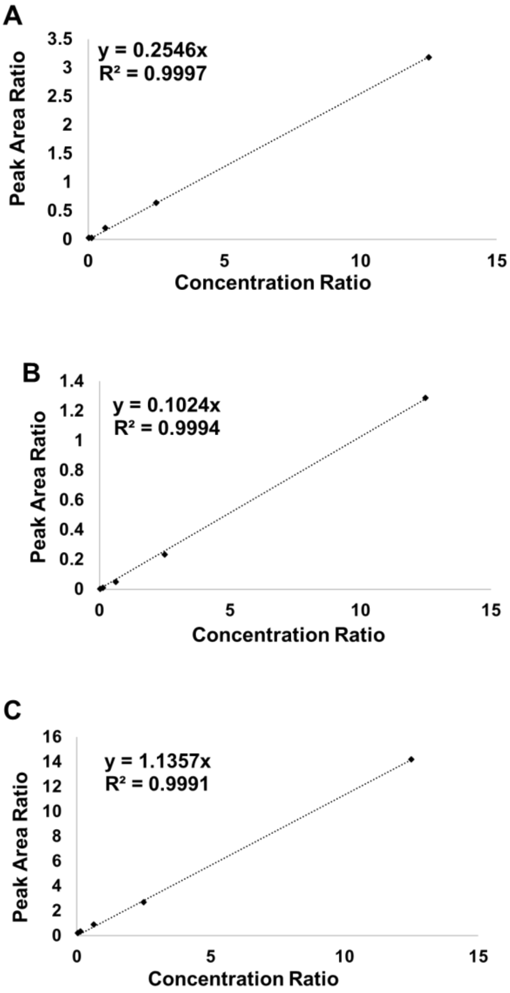

Figure S7. Standard curve of ABG (A), AMBG (B) and O^6-BG (C) which was constructed by plotting SRM peak area rations between ABG/AMBG/O^6-BG and D_6-O^6-BG versus the concentration of ABG/AMBG/O^6-BG and D_6-O^6-BG

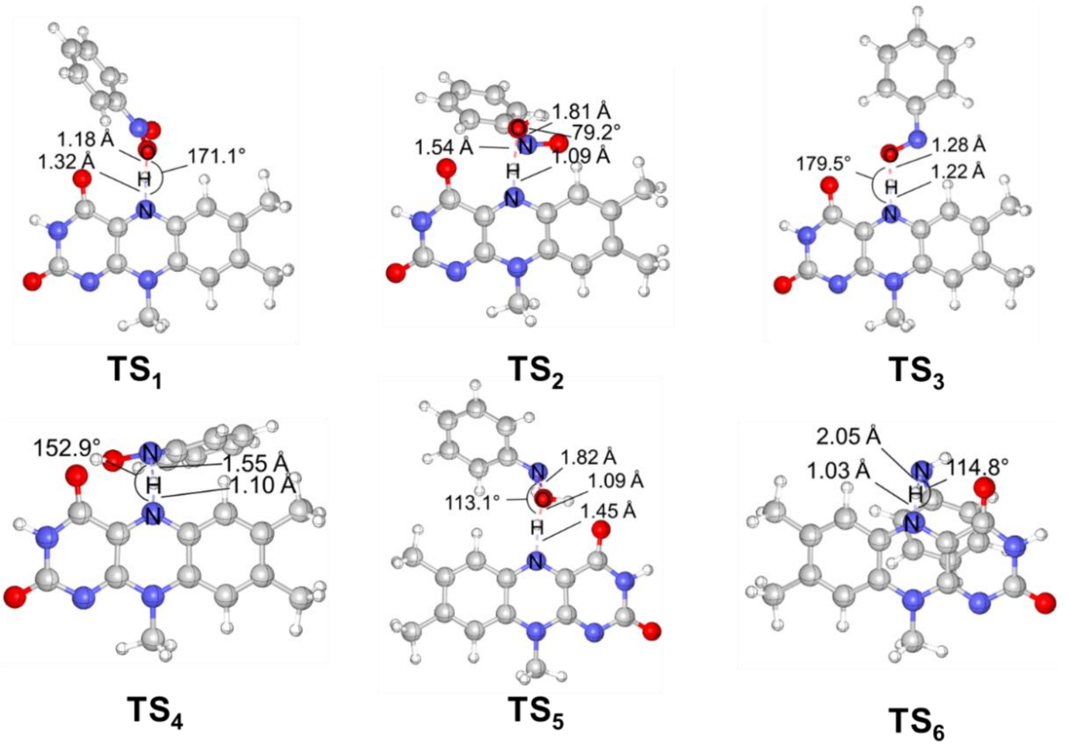

Figure S8. Optimized geometries and main parameters of the TSs in reduction of nitrobenzene mediated by FMNH calculated at the B3LYP/6-31+G (d, p) theoretical level

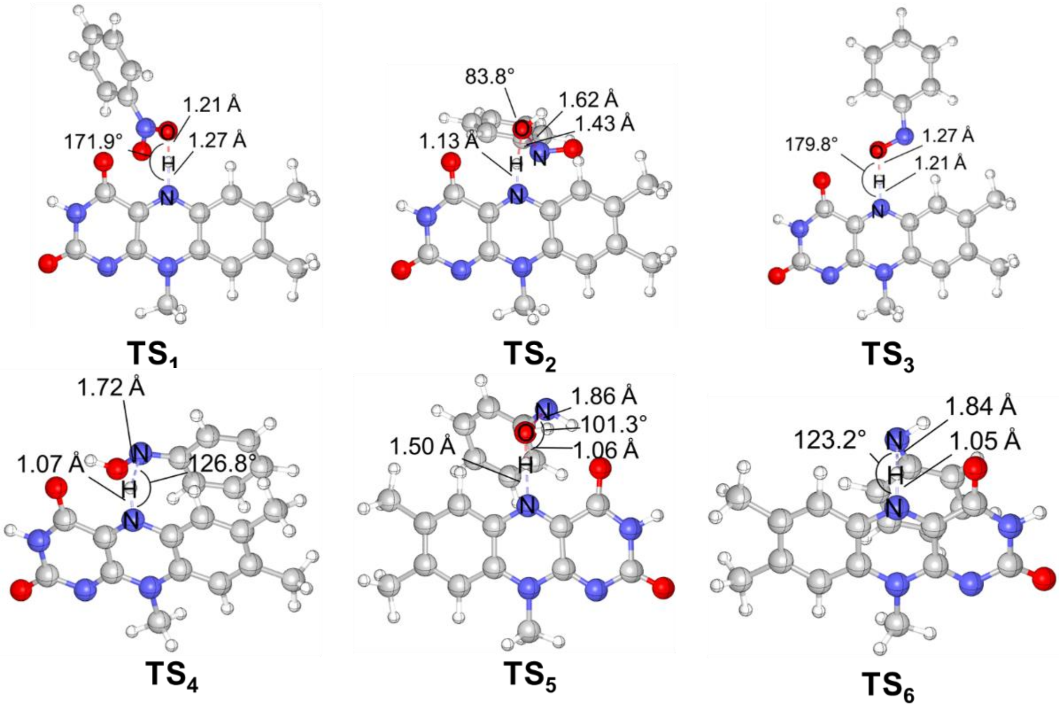

Figure S9. Optimized geometries and main parameters of the TSs in reduction of nitrobenzene mediated by FMNH calculated at the M062X/6-31+G (d, p) theoretical level

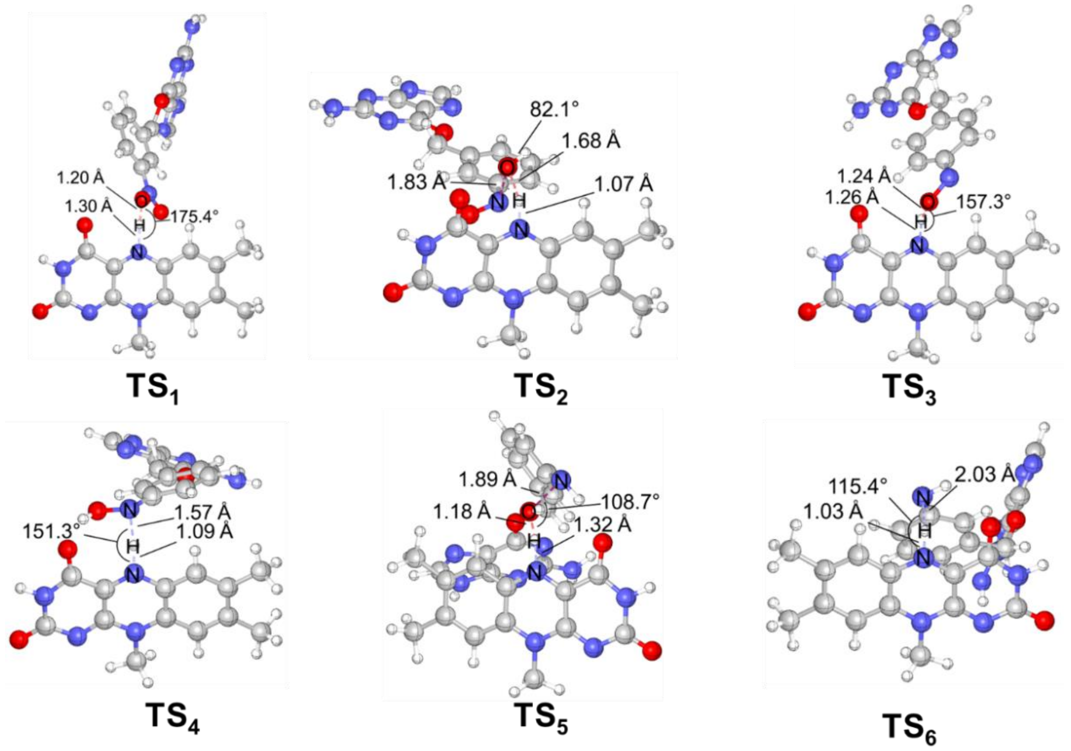

Figure S10. Optimized geometries and main parameters of the TSs in reduction of 3-NBG mediated by FMNH calculated at the B3LYP/6-31+G (d, p) theoretical level

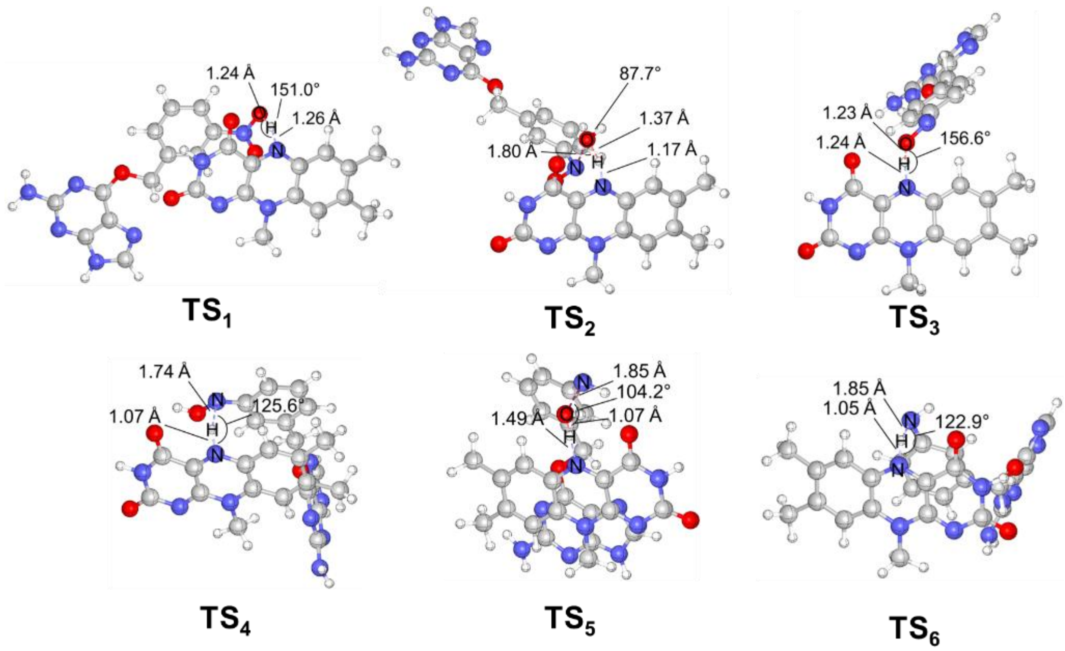

Figure S11. Optimized geometries and main parameters of the TSs in reduction of 3-NBG mediated by FMNH calculated at the M062X/6-31+G (d, p) theoretical level

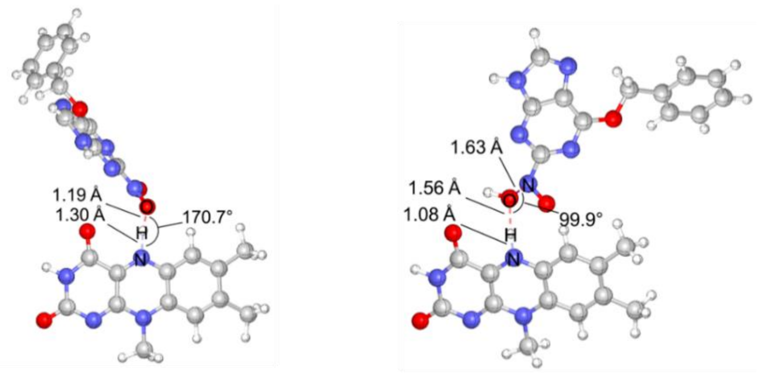

TS$_1$ **TS$_2$**

Figure S12. Optimized geometries and main parameters of the TSs in reduction of 2-NBP mediated by FMNH calculated at the B3LYP/6-31+G (d, p) theoretical level

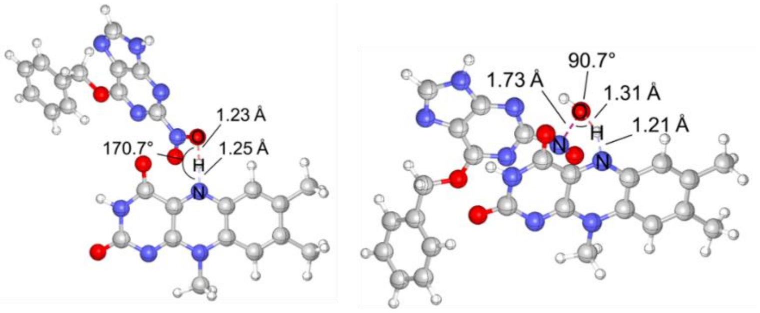

TS$_1$ **TS$_2$**

Figure S13. Optimized geometries and main parameters of the TSs in reduction of 2-NBP mediated by FMNH calculated at the M062X/6-31+G (d, p) theoretical level

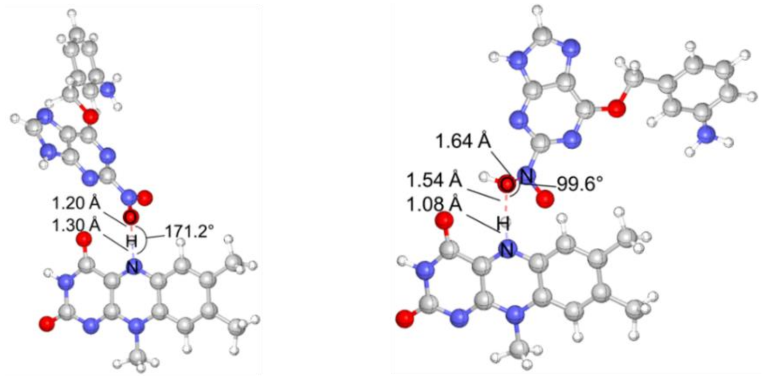

TS$_1$ **TS$_2$**

Figure S14. Optimized geometries and main parameters of the TSs in reduction of ANBP mediated by FMNH calculated at the B3LYP/6-31+G (d, p) theoretical level

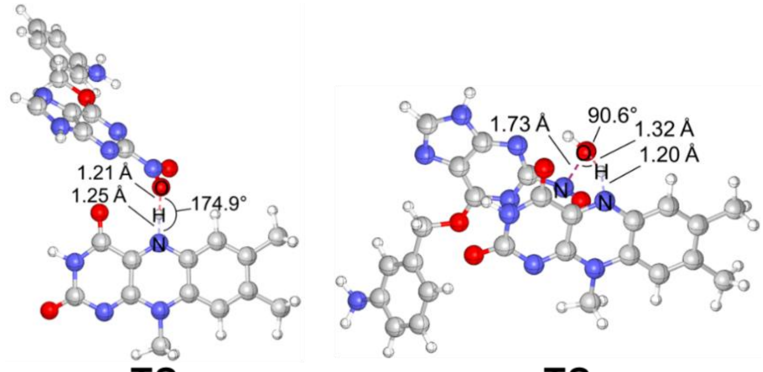

TS_1 TS_2

Figure S15. Optimized geometries and main parameters of the TSs in reduction of ANBP mediated by FMNH calculated at the M062X/6-31+G (d, p) theoretical level.

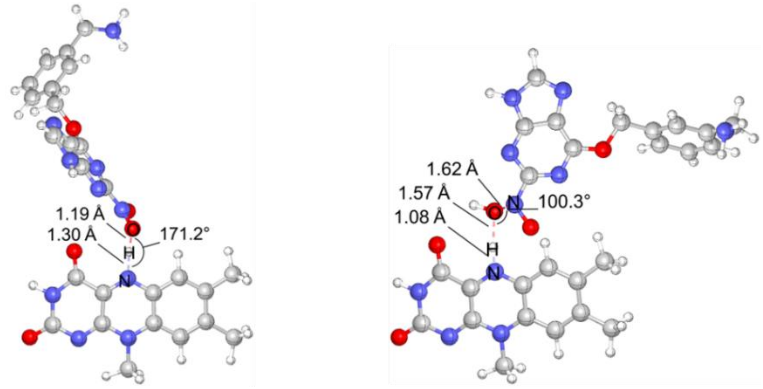

TS_1 TS_2

Figure S16. Optimized geometries and main parameters of the TSs in reduction of AMNBP mediated by FMNH calculated at the B3LYP/6-31+G (d, p) theoretical level

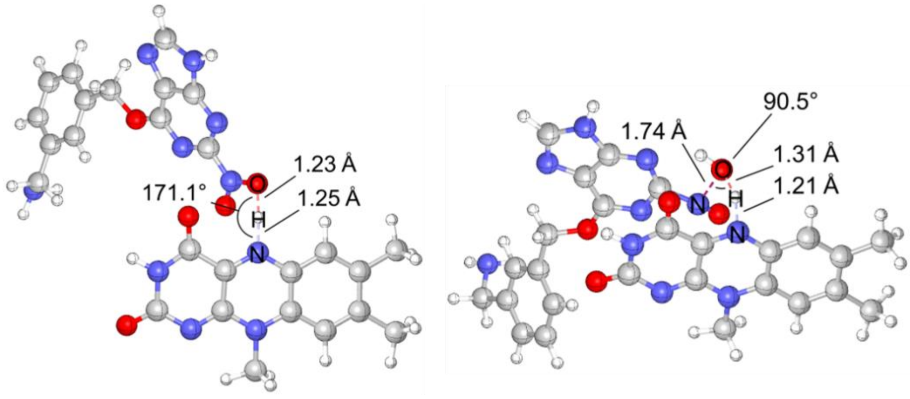

TS_1 TS_2

Figure S17. Optimized geometries and main parameters of the TSs in reduction of AMNBP mediated by FMNH calculated at the M062X/6-31+G (d, p) theoretical level

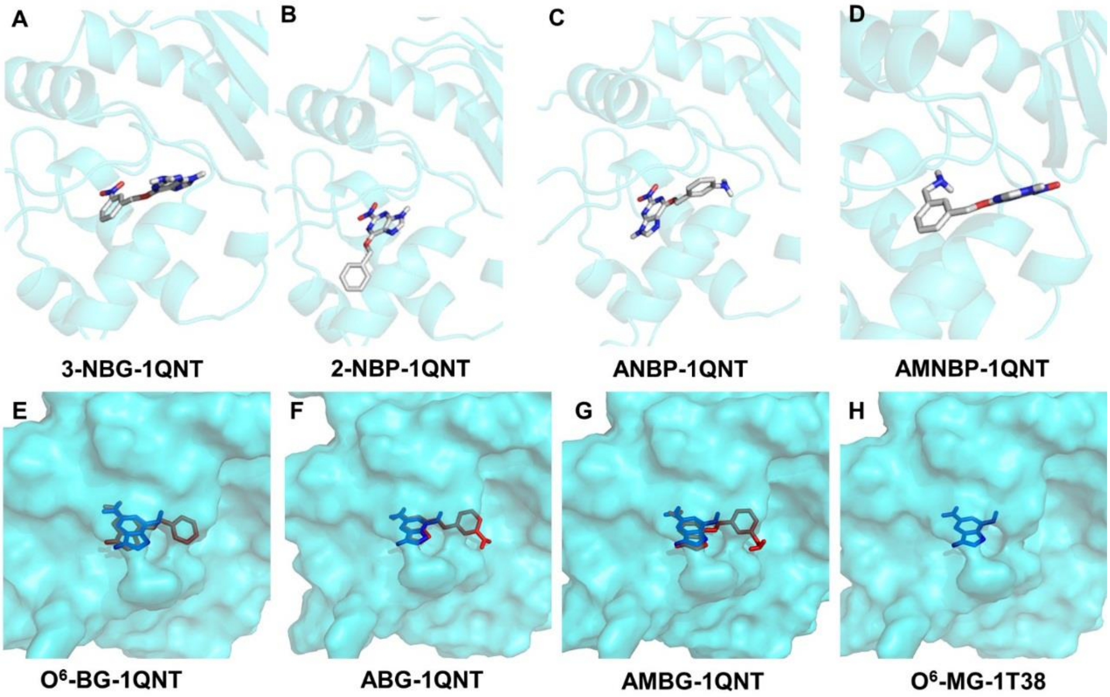

Figure S18. Molecular docking results of 3-NBG or 2-NBP or ANBP or AMNBP or O^6-BG or ABG or AMBG and AGT. Key hydrogen bonding interactions between 3-NBG (**A**) or 2-NBP (**B**) or ANBP (**C**) or AMNBP (**D**) and the amino acid residues in the active pocket of AGT. 3-NBG, 2-NBP, ANBP and AMNBP are present in the stick model with white representing carbon atoms. The remaining protein displayed in the cartoon model. Overlap of the ligand between the docked pose with red (O^6-BG (**E**) or ABG (**F**) or AMBG (**G**) or O^6-MG (**H**)) and the pose with blue (O^6-MG in the 1T38). The protein is displayed as solid surface with cyan. The ligands are presented in stick models. Nonpolar hydrogens are hidden. All figures were generated using *PyMOL* software (Educational version; www.pymol.org; DeLano Scientific, Scan Carlas, CA, USA).

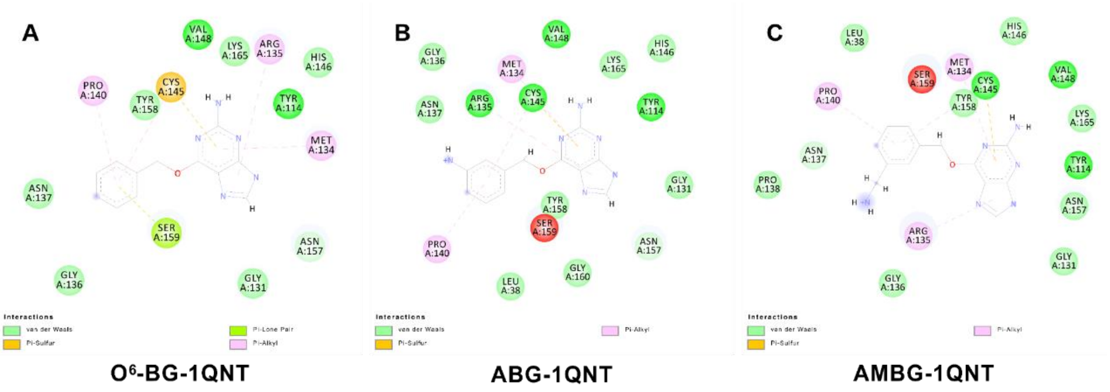

Figure S19. 2D representation of the binding interactions between O^6-BG (**A**) or ABG (**B**) or AMBG (**C**) and the amino acid residues in the active pocket of AGT obtained from molecular docking.

Table S1. Concentrations of reduction product of 3-NBG under different conditions with indicated treatment time

Time (h)	0.25	0.5	0.75	1	1.5	2	2.5	3
Hypoxia (nM)	10.2[a]	10.1	10.8	13.6	15.1	20.9	20.7	19.6
	(11.7)[b]	(11.8)	(15.4)	(22.3)	(23.2)	(24.3)	(25.3)	(37.3)
Normoxia (nM)	3.3	6.5	6.2	5.2	5.5	5.6	5.4	5.4
	(3.1)	(4.4)	(5.9)	(6.5)	(5.3)	(5.3)	(6.4)	(6.7)
Hypoxia/Normoxia	3.14	1.66	1.74	2.63	2.72	3.73	3.84	3.61
	(3.84)	(2.69)	(2.60)	(3.44)	(4.45)	(4.58)	(3.93)	(5.55)

[a] 3-NBG concentration is 5 mM

[b] 3-NBG concentration is 10 mM

Table S2. Concentrations of reduction product of ANBP under different conditions with indicated treatment time

Time (h)	0.25	0.5	0.75	1	1.5	2	2.5	3
Hypoxia (nM)	305.6[a]	448.7	435.9	362.1	407.7	481.1	407.7	509.4
	(623.7)[b]	(834.8)	(755.8)	(860.6)	(963.6)	(976.9)	(1074.4)	(929.6)
Normoxia (nM)	269.9	298.0	309.1	268.0	334.7	327.3	293.78	383.7
	(534.2)	(484.4)	(646.5)	(681.6)	(587.3)	(679.9)	(737.6)	(705.0)
Hypoxia/Normoxia	1.13	1.50	1.41	1.35	1.22	1.47	1.38	1.33
	(1.17)	(1.72)	(1.17)	(1.26)	(1.64)	(1.44)	(1.46)	(1.31)

[a] ANBP concentration is 5 mM

[b] ANBP concentration is 10 mM

Table S3. Concentrations of reduction product of 2-NBP under different conditions with indicated treatment time

Time (h)	0.25	0.5	0.75	1	1.5	2	2.5	3
Hypoxia (nM)	66.6[a]	112.0	107.2	128.2	123.9	133.5	135.8	149.8
	(56.9)[b]	(127.2)	(120.6)	(140.9)	(140.7)	(154.2)	(186.8)	(190.0)
Normoxia (nM)	11.8	20.9	25.6	25.3	20.6	24.4	35.9	40.2
	(21.9)	(32.3)	(25.3)	(28.9)	(29.6)	(38.9)	(39.1)	(52.2)
Hypoxia/Normoxia	5.63	5.35	4.19	5.07	6.01	5.47	3.78	3.73
	(2.60)	(3.94)	(4.77)	(4.88)	(4.76)	(3.96)	(4.78)	(3.64)

[a] 2-NBP concentration is 5 mM

[b] 2-NBP concentration is 10 mM

Table S4. Concentrations of reduction product of AMNBP under different conditions with indicated treatment time

Time (h)	0.25	0.5	0.75	1	1.5	2	2.5	3
Hypoxia (nM)	56.8[a]	130.4	137.8	131.8	160.8	180.7	158.2	187.9
	(233.3)[b]	(332.9)	(340.4)	(354.0)	(477.1)	(580.9)	(551.0)	(522.4)
Normoxia (nM)	37.4	85.2	91.7	108.0	103.4	103.9	107.3	132.7
	(223.6)	(237.5)	(247.0)	(247.4)	(327.5)	(352.4)	(325.4)	(388.9)
Hypoxia/Normoxia	1.52	1.53	1.50	1.22	1.55	1.74	1.47	1.42
	(1.04)	(1.40)	(1.38)	(1.43)	(1.46)	(1.65)	(1.69)	(1.34)

[a] AMNBP concentration is 5 mM

[b] AMNBP concentration is 10 mM

Table S5. Relative energies (*E*), enthalpies (*H*) and Gibbs free energies (*G*) for the reduction of nitrobenzene mediated by FMNH [a]

Summary of Energetic	E_{0K} [b]	E_{298K} [b]	H_{298Kb}	G_{298K} [b]	G [c]	E [c]
**RC+FMNH**	0.00[d]	0.00	0.00	0.00	0.00[f]	0.00
	(0.00)[e]	(0.00)	(0.00)	(0.00)	(0.00)[h]	(0.00)
**TS1-(RC+FMNH)**	7.48	8.41	7.82	18.47	23.35	12.36
	(7.69)	(8.48)	(7.89)	(19.55)	(25.99)	(14.13)
**IC1+FMN-(RC+FMNH)**	9.56	10.00	10.00	8.76	9.60	10.39
	(8.01)	(8.47)	(8.47)	(7.12)	(8.21)	(9.10)
**TS2+FMN-(RC+2FMNH)**	25.45	26.36	25.76	38.32	42.15	29.28
	(27.89)	(28.56)	(27.97)	(41.07)	(44.78)	(31.59)
**IC2+2FMN+H_2O**	-14.07	-13.18	-12.59	-23.63	-26.74	-17.18
**-(RC+2FMNH)**	(-14.18)	(-13.23)	(-12.64)	(-23.87)	(-25.35)	(-15.65)
**TS3+2FMN+H_2O**	-13.88	-12.25	-12.26	-11.63	-10.67	-12.92
**-(RC+3FMNH)**	(-9.51)	(-7.64)	(-7.65)	(-8.28)	(-8.20)	(-9.42)
**IC3+3FMN+H_2O**	-10.95	-10.02	-9.43	-20.67	-25.92	-16.19
**-(RC+3FMNH)**	(-9.84)	(-8.94)	(-8.35)	(-19.60)	(-23.13)	(-13.37)
**TS4+3FMN+H_2O**	-14.78	-13.62	-13.62	-10.83	-9.78	-13.73
**-(RC+4FMNH)**	(-17.73)	(-16.58)	(-16.58)	(-12.51)	(-10.60)	(-15.74)
**IC4+4FMN+H_2O**	-30.08	-29.26	-28.67	-39.11	-43.93	-34.90
**-(RC+4FMNH)**	(-33.69)	(-32.85)	(-32.26)	(-42.89)	(-45.38)	(-36.18)
**TS5+4FMN+H_2O**	-15.02	-13.42	-13.42	-13.44	-12.62	-14.19
**-(RC+5FMNH)**	(-16.00)	(-14.47)	(-14.48)	(-12.52)	(-9.04)	(-12.53)
**IC5+5FMN+2H_2O**	-43.87	-42.46	-41.28	-62.41	-71.24	-52.69
**-(RC+5FMNH)**	(-39.64)	(-38.11)	(-36.93)	(-58.56)	(-64.60)	(-45.68)

**TS$_6$+5FMN+2H$_2$O**	-41.54	-39.50	-38.92	-46.55	-52.19	-47.17
**-(RC+6FMNH)**	(-49.24)	(-47.37)	(-46.78)	(-53.54)	(-55.77)	(-51.45)
**PC+6FMN+2H$_2$O**	-74.02	-72.59	-71.41	-91.83	-101.52	-83.71
**-(RC+6FMNH)**	(-74.80)	(-73.23)	(-72.05)	(-93.05)	(-99.29)	(-81.03)

[a] All energies are in kcal/mol.

[b] Relative energies calculated at the B3LYP/6-31+G(d,p)[d] and M062X/6-31+G(d,p)[e] theoretical level in gas phase.

[c] Relative energies calculated at the CPCM-B3LYP/6-311+G(3df,2p)//B3LYP/6-31+G(d,p)[f] and CPCM-M062X/6-311+G(3df,2p)//M062X/6-31+G(d,p)[h] theoretical level in aqueous phase.

Table S6. Relative energies (*E*), enthalpies (*H*) and Gibbs free energies (*G*) for the reduction of 3-NBG mediated by FMNH [a]

**Summary of Energetic**	E_{0K}^{b}	E_{298K}^{b}	H_{298Kb}	G_{298K}^{b}	G^{c}	E^{c}
**RC+FMNH**	0.00[d]	0.00	0.00	0.00	0.00[f]	0.00
	(0.00)[e]	(0.00)	(0.00)	(0.00)	(0.00)[h]	(0.00)
**TS$_1$-(RC+FMNH)**	10.45	11.44	10.85	21.82	26.16	14.19
	(8.29)	(8.84)	(8.25)	(22.54)	(29.36)	(15.11)
**IC$_1$+FMN-(RC+FMNH)**	10.03	10.46	10.46	9.44	9.77	10.35
	(8.44)	(8.95)	(8.95)	(7.70)	(8.37)	(9.11)
**TS$_2$+FMN-(RC+2FMNH)**	26.90	27.86	27.27	40.28	42.90	29.52
	(23.90)	(24.57)	(23.97)	(38.40)	(45.24)	(30.73)
**IC$_2$+2FMN+H$_2$O**	-13.98	-13.11	-12.51	-23.26	-26.53	-17.25
**-(RC+2FMNH)**	(-13.99)	(-13.04)	(-12.45)	(-23.53)	(-25.23)	(-15.69)
**TS$_3$+2FMN+H$_2$O**	-3.81	-2.14	-2.14	-0.96	-1.71	-4.56
**-(RC+3FMNH)**	(-2.77)	(-1.11)	(-1.12)	(0.46)	(-0.69)	(-3.91)
**IC$_3$+3FMN+H$_2$O**	-17.11	-16.35	-15.75	-26.31	-30.26	-21.06
**-(RC+3FMNH)**	(-13.91)	(-13.11)	(-12.52)	(-23.19)	(-25.14)	(-15.86)
**TS$_4$+3FMN+H$_2$O**	-12.22	-11.00	-11.00	-8.06	-7.38	-11.54
**-(RC+4FMNH)**	(-20.38)	(-19.40)	(-19.40)	(-13.22)	(-9.02)	(-16.18)
**IC$_4$+4FMN+H$_2$O**	-30.11	-29.34	-28.75	-38.94	-44.03	-35.21
**-(RC+4FMNH)**	(-31.39)	(-30.61)	(-30.02)	(-40.26)	(-43.24)	(-34.38)
**TS$_5$+4FMN+H$_2$O**	-17.13	-16.08	-16.08	-11.91	-15.39	-15.58
**-(RC+5FMNH)**	(-22.98)	(-22.08)	(-22.08)	(-15.75)	(-6.86)	(-14.88)
**IC$_5$+5FMN+2H$_2$O**	-43.39	-42.08	-40.89	-61.60	-71.13	-52.92
**-(RC+5FMNH)**	(-37.03)	(-35.58)	(-34.40)	(-55.42)	(-61.80)	(-43.40)
**TS$_6$+5FMN+2H$_2$O**	-40.18	-38.32	-37.72	-44.06	-50.26	-46.38
**-(RC+6FMNH)**	(-49.44)	(-47.89)	(-47.30)	(-51.59)	(-54.30)	(-51.82)

**PC+6FMN+2H_2O**	-73.38	-72.05	-70.87	-90.96	-102.07	-86.49
**-(RC+6FMNH)**	(-71.93)	(-70.48)	(-69.30)	(-89.16)	(-96.63)	(-78.94)

[a] All energies are in kcal/mol.

[b] Relative energies calculated at the B3LYP/6-31+G(d,p)[d] and M062X/6-31+G(d,p)[e] theoretical level in gas phase.

[c] Relative energies calculated at the CPCM-B3LYP/6-311+G(3df,2p)//B3LYP/6-31+G(d,p)[f] and CPCM-M062X/6-311+G(3df,2p)//M062X/6-31+G(d,p)[h] theoretical level in aqueous phase.

Table S7. Relative energies (*E*), enthalpies (*H*) and Gibbs free energies (*G*) for the reduction of 2-NBP mediated by FMNH [a]

Summary of Energetic	E_{0K} [b]	E_{298K} [b]	H_{298Kb}	G_{298K} [b]	G [c]	E [c]
**RC+FMNH**	0.00[d]<br>(0.00)[e]	0.00<br>(0.00)	0.00<br>(0.00)	0.00<br>(0.00)	0.00[f]<br>(0.00)[h]	0.00<br>(0.00)
**TS_1-(RC+FMNH)**	6.91<br>(6.66)	7.84<br>(7.37)	7.24<br>(6.78)	18.34<br>(20.34)	21.55<br>(27.34)	10.11<br>(13.66)
**IC_1+FMN-(RC+FMNH)**	6.62<br>(4.74)	6.94<br>(5.03)	6.94<br>(5.03)	6.64<br>(5.25)	6.96<br>(6.40)	6.93<br>(5.89)
**TS_2+FMN-(RC+2FMNH)**	19.27<br>(13.91)	20.19<br>(14.37)	19.60<br>(13.78)	32.37<br>(30.43)	37.91<br>(40.88)	24.80<br>(24.36)
**IC_2+2FMN+H_2O -(RC+2FMNH)**	-14.49<br>(-15.27)	-13.61<br>(-14.36)	-13.02<br>(-13.77)	-23.71<br>(-24.49)	-27.02<br>(-26.29)	-17.79<br>(-17.06)

[a] All energies are in kcal/mol.

[b] Relative energies calculated at the B3LYP/6-31+G(d,p)[d] and M062X/6-31+G(d,p)[e] theoretical level in gas phase.

[c] Relative energies calculated at the CPCM-B3LYP/6-311+G(3df,2p)//B3LYP/6-31+G(d,p)[f] and CPCM-M062X/6-311+G(3df,2p)//M062X/6-31+G(d,p)[h] theoretical level in aqueous phase.

Table S8. Relative energies (*E*), enthalpies (*H*) and Gibbs free energies (*G*) for the reduction of ANBP mediated by FMNH [a]

**Summary of Energetic**	E_{0K} [b]	E_{298K} [b]	H_{298K} [b]	G_{298K} [b]	G [c]	E [c]
**RC+FMNH**	0.00[d] (0.00)[e]	0.00 (0.00)	0.00 (0.00)	0.00 (0.00)	0.00[f] (0.00)[h]	0.00 (0.00)
**TS1-(RC+FMNH)**	6.52 (6.60)	7.44 (7.50)	6.85 (6.90)	18.41 (18.89)	22.37 (27.07)	10.48 (14.78)
**IC1+FMN-(RC+FMNH)**	6.72 (5.13)	7.06 (5.50)	7.06 (5.50)	6.28 (4.99)	6.50 (5.60)	6.94 (5.73)
**TS2+FMN-(RC+2FMNH)**	18.98 (15.09)	19.88 (15.65)	19.29 (15.05)	31.66 (30.57)	37.26 (40.29)	24.59 (24.81)
**IC2+2FMN+H_2O -(RC+2FMNH)**	-14.40 (-15.25)	-13.54 (-14.25)	-12.95 (-13.66)	-23.58 (-25.06)	-26.92 (-26.57)	-17.73 (-16.76)

[a] All energies are in kcal/mol.

[b] Relative energies calculated at the B3LYP/6-31+G(d,p)[d] and M062X/6-31+G(d,p)[e] theoretical level in gas phase.

[c] Relative energies calculated at the CPCM-B3LYP/6-311+G(3df,2p)//B3LYP/6-31+G(d,p)[f] and CPCM-M062X/6-311+G(3df,2p)//M062X/6-31+G(d,p)[h] theoretical level in aqueous phase.

Table S9. Relative energies (*E*), enthalpies (*H*) and Gibbs free energies (*G*) for the reduction of AMNBP mediated by FMNH [a]

Summary of Energetic	E_{0K} [b]	E_{298K} [b]	H_{298K} [b]	G_{298K} [b]	G [c]	E [c]
**RC+FMNH**	0.00[d]<br>(0.00)[e]	0.00<br>(0.00)	0.00<br>(0.00)	0.00<br>(0.00)	0.00[f]<br>(0.00)[h]	0.00<br>(0.00)
**TS_1-(RC+FMNH)**	7.03<br>(6.31)	7.91<br>(7.08)	7.32<br>(6.49)	19.21<br>(19.45)	22.24<br>(22.16)	10.06<br>(9.02)
**IC_1+FMN-(RC+FMNH)**	6.75<br>(5.04)	7.07<br>(5.38)	7.07<br>(5.38)	6.79<br>(4.73)	7.02<br>(5.67)	6.97<br>(5.98)
**TS_2+FMN-(RC+2FMNH)**	19.44<br>(13.68)	20.42<br>(14.23)	19.83<br>(13.63)	31.83<br>(29.72)	37.00<br>(40.71)	24.61<br>(24.66)
**IC_2+2FMN+H_2O<br>-(RC+2FMNH)**	-14.44<br>(-15.72)	-13.57<br>(-14.73)	-12.98<br>(-14.14)	-23.60<br>(-25.67)	-26.94<br>(-26.78)	-17.78<br>(-16.83)

[a] All energies are in kcal/mol.

[b] Relative energies calculated at the B3LYP/6-31+G(d,p)[d] and M062X/6-31+G(d,p)[e] theoretical level in gas phase.

[c] Relative energies calculated at the CPCM-B3LYP/6-311+G(3df,2p)//B3LYP/6-31+G(d,p)[f] and CPCM-M062X/6-311+G(3df,2p)//M062X/6-31+G(d,p)[h] theoretical level in aqueous phase.

Appendix A. XYZ coordinates for all RCs, ICs, PCs and TSs

The reduction of nitrobenzene mediated by FMNH calculated at the B3LYP/6-31+G(d,p) theoretical level

RC

0 1

Standard orientation:

Center Number	Atomic Number	Atomic Type	Coordinates (Angstroms) X	Y	Z
1	6	0	0.000000	1.213231	-1.823245
2	6	0	0.000000	0.000000	-2.519575
3	6	0	0.000000	-1.213231	-1.823245
4	6	0	0.000000	-1.222181	-0.428529
5	6	0	0.000000	0.000000	0.244400
6	6	0	0.000000	1.222181	-0.428529
7	7	0	0.000000	0.000000	1.719353
8	8	0	0.000000	1.089987	2.293640
9	8	0	0.000000	-1.089987	2.293640
10	1	0	0.000000	2.153680	-2.365083
11	1	0	0.000000	0.000000	-3.605410
12	1	0	0.000000	-2.153680	-2.365083
13	1	0	0.000000	-2.145619	0.137101
14	1	0	0.000000	2.145619	0.137101

IC_1

0 2

Standard orientation:

Center Number	Atomic Number	Atomic Type	Coordinates (Angstroms) X	Y	Z
1	6	0	1.915534	1.180713	-0.000042
2	6	0	2.580579	-0.052435	-0.000051
3	6	0	1.838170	-1.238959	-0.000023
4	6	0	0.445719	-1.210699	0.000059
5	6	0	-0.207787	0.036643	0.000084
6	6	0	0.526062	1.239289	0.000034
7	8	0	-2.294455	1.163927	-0.000073
8	8	0	-2.275193	-1.116228	-0.000070
9	1	0	2.484611	2.105511	-0.000050
10	1	0	3.665442	-0.087531	-0.000098

11	1	0	2.346875	-2.198295	-0.000031
12	1	0	-0.131485	-2.126073	0.000103
13	1	0	-0.005158	2.183227	0.000065
14	1	0	-3.205104	-0.826849	-0.000361
15	7	0	-1.598236	0.120158	0.000164

IC_2

0 1

Standard orientation:

Center Number	Atomic Number	Atomic Type	Coordinates (Angstroms) X	Y	Z
1	6	0	1.275531	1.339057	0.000000
2	6	0	2.176136	0.261414	0.000002
3	6	0	1.710022	-1.056556	0.000002
4	6	0	0.336094	-1.301553	0.000000
5	6	0	-0.094666	1.103756	-0.000003
6	8	0	-2.733772	0.352395	0.000007
7	1	0	1.652562	2.357472	0.000000
8	1	0	3.244875	0.455263	0.000004
9	1	0	2.412647	-1.883970	0.000003
10	1	0	-0.066055	-2.310113	0.000001
11	1	0	-0.816454	1.913638	-0.000005
12	7	0	-1.948755	-0.584852	-0.000005
13	6	0	-0.555802	-0.222365	-0.000003

IC_3

0 2

Standard orientation:

Center Number	Atomic Number	Atomic Type	Coordinates (Angstroms) X	Y	Z
1	6	0	1.788053	1.020309	0.035786
2	6	0	2.225759	-0.313176	0.039751
3	6	0	1.286553	-1.350818	-0.014511
4	6	0	-0.076852	-1.075244	-0.060171
5	6	0	-0.530752	0.270329	-0.043305
6	6	0	0.432938	1.313171	-0.011197
7	1	0	2.511829	1.829144	0.068756

8	1	0	3.286541	-0.540748	0.074122
9	1	0	1.622450	-2.383486	-0.036232
10	1	0	-0.768854	-1.908126	-0.153360
11	1	0	0.073716	2.337090	-0.013299
12	8	0	-2.781012	-0.284021	0.068993
13	1	0	-2.371823	-1.121822	0.357649
14	7	0	-1.837137	0.696099	-0.075385

IC_4

0 1

Standard orientation:

Center Number	Atomic Number	Atomic Type	Coordinates (Angstroms) X	Y	Z
1	6	0	-1.342672	1.350879	0.013488
2	6	0	-2.285533	0.321993	-0.040331
3	6	0	-1.844360	-1.005749	-0.045272
4	6	0	-0.482743	-1.299659	0.004012
5	6	0	0.460383	-0.260287	0.066758
6	6	0	0.025374	1.070649	0.068109
7	1	0	-1.669627	2.387004	0.009728
8	1	0	-3.346328	0.548424	-0.082642
9	1	0	-2.562700	-1.819285	-0.093148
10	1	0	-0.148230	-2.334525	0.003392
11	1	0	0.754622	1.871041	0.095008
12	8	0	2.710923	0.418586	-0.249824
13	1	0	3.251190	0.614601	0.528624
14	7	0	1.827625	-0.607129	0.210566
15	1	0	2.057613	-1.432997	-0.336908

IC_5

0 2

Standard orientation:

Center Number	Atomic Number	Atomic Type	Coordinates (Angstroms) X	Y	Z
1	6	0	-1.081967	1.236126	-0.000074
2	6	0	-1.810698	0.032925	0.000340
3	6	0	-1.134681	-1.200694	0.000159

4	6	0	0.249072	-1.240097	-0.000357
5	6	0	1.020892	-0.030143	-0.000462
6	6	0	0.302711	1.214389	-0.000350
7	1	0	-1.609297	2.185889	-0.000114
8	1	0	-2.896275	0.057443	0.000976
9	1	0	-1.703337	-2.126133	-0.000113
10	1	0	0.792553	-2.179472	-0.000665
11	1	0	0.869218	2.142678	-0.000290
12	7	0	2.357233	-0.136577	0.000484
13	1	0	2.774538	0.800596	0.001281

PC

0 1

Standard orientation:

Center Number	Atomic Number	Atomic Type	Coordinates (Angstroms) X	Y	Z
1	6	0	1.136445	1.240084	0.013562
2	6	0	1.881463	0.053127	0.012088
3	6	0	1.226126	-1.185480	-0.005502
4	6	0	-0.174230	-1.237128	-0.021617
5	6	0	-0.919247	-0.050171	-0.020142
6	6	0	-0.263910	1.188436	-0.002553
7	1	0	1.636810	2.185788	0.026992
8	1	0	2.950665	0.092562	0.024392
9	1	0	1.794963	-2.091748	-0.006627
10	1	0	-0.674594	-2.182832	-0.035047
11	1	0	-0.832747	2.094704	-0.001427
12	7	0	-2.388151	-0.104347	-0.037046
13	1	0	-2.731797	-0.124432	0.901839
14	1	0	-2.746123	0.703181	-0.505825

TS_1

0 2

Standard orientation:

Center Number	Atomic Number	Atomic Type	Coordinates (Angstroms) X	Y	Z
1	7	0	-1.945409	3.363328	-0.024443

2	6	0	-0.815698	4.148733	-0.096205
3	8	0	-0.838175	5.370920	-0.113839
4	7	0	0.436860	3.490336	-0.150108
5	6	0	0.661769	2.130458	-0.142030
6	8	0	1.786241	1.637091	-0.175912
7	6	0	-0.580885	1.338327	-0.072716
8	7	0	-0.501079	0.011360	-0.068922
9	6	0	-1.649653	-0.738089	-0.002377
10	6	0	-1.571788	-2.145987	-0.014350
11	6	0	-2.707985	-2.939441	0.050604
12	6	0	-2.587917	-4.444098	0.036799
13	6	0	-3.979855	-2.307191	0.122306
14	6	0	-5.242266	-3.129845	0.188121
15	6	0	-4.066203	-0.913500	0.125621
16	6	0	-2.920212	-0.105770	0.063931
17	7	0	-2.984862	1.285090	0.064850
18	6	0	-1.831416	2.055745	-0.011144
19	6	0	-4.287242	1.955351	0.134818
20	1	0	1.251993	4.091415	-0.198584
21	1	0	0.631323	-0.625727	0.184689
22	1	0	-0.587461	-2.596796	-0.082116
23	1	0	-3.013316	-4.890923	0.943455
24	1	0	-1.541540	-4.751379	-0.027950
25	1	0	-3.120064	-4.883832	-0.815179
26	1	0	-5.347265	-3.771704	-0.694905
27	1	0	-5.242616	-3.792967	1.061444
28	1	0	-6.128943	-2.493938	0.248057
29	1	0	-5.046538	-0.456970	0.179296
30	1	0	-4.804391	1.668942	1.055463
31	1	0	-4.110115	3.027762	0.128045
32	6	0	6.115579	-0.994248	-0.712575
33	6	0	4.787718	-1.238734	-1.045448
34	6	0	3.796004	-1.052043	-0.066400
35	6	0	4.123191	-0.622200	1.230443
36	6	0	5.459077	-0.382457	1.540272
37	6	0	6.458071	-0.567132	0.577278
38	1	0	6.888311	-1.135587	-1.462036
39	1	0	4.499094	-1.564580	-2.036981
40	1	0	3.336161	-0.472661	1.957005
41	1	0	5.721478	-0.043887	2.537896
42	1	0	7.497048	-0.376480	0.828371
43	7	0	2.451269	-1.306877	-0.412661

44	8	0	2.104350	-1.610259	-1.569948
45	8	0	1.554564	-1.247262	0.562213
46	1	0	-4.898185	1.675468	-0.728781

TS_2

0 1

Standard orientation:

Center Number	Atomic Number	Atomic Type	Coordinates (Angstroms) X	Y	Z
1	1	0	1.008256	-2.426650	2.188963
2	6	0	4.226099	-2.782250	-1.767270
3	6	0	2.918338	-2.780932	-1.278515
4	6	0	2.556376	-1.847754	-0.299352
5	6	0	3.479698	-0.915165	0.188461
6	6	0	4.782988	-0.934580	-0.304789
7	6	0	5.160517	-1.861833	-1.284257
8	1	0	4.514114	-3.505457	-2.525011
9	1	0	2.176228	-3.484686	-1.637244
10	1	0	3.180085	-0.215304	0.960435
11	1	0	5.508675	-0.223915	0.079780
12	1	0	6.176833	-1.865557	-1.667338
13	7	0	1.169156	-1.767757	0.113628
14	8	0	0.432280	-2.734318	-0.157486
15	8	0	1.294058	-1.544294	1.902428
16	7	0	-0.427769	3.552029	-0.603873
17	6	0	0.723804	4.133115	-0.119256
18	8	0	1.087369	5.262958	-0.406578
19	7	0	1.517708	3.366604	0.769468
20	6	0	1.272991	2.083660	1.205535
21	8	0	2.001227	1.486914	1.992260
22	6	0	0.042101	1.510555	0.635345
23	7	0	-0.299758	0.252324	0.923995
24	6	0	-1.507914	-0.264289	0.514397
25	6	0	-1.936734	-1.531944	0.950347
26	6	0	-3.156862	-2.060787	0.551695
27	6	0	-3.586223	-3.423636	1.036819
28	6	0	-3.973055	-1.303679	-0.327390
29	6	0	-5.299366	-1.845244	-0.796925
30	6	0	-3.553378	-0.043275	-0.762129
31	6	0	-2.330492	0.504446	-0.352721

32	7	0	-1.904172	1.764199	-0.760538
33	6	0	-0.743205	2.328915	-0.250033
34	6	0	-2.720663	2.542174	-1.698734
35	1	0	2.349142	3.822900	1.128463
36	1	0	0.342135	-0.388974	1.532041
37	1	0	-1.296053	-2.086457	1.621028
38	1	0	-4.539333	-3.381269	1.577282
39	1	0	-2.838055	-3.852964	1.706920
40	1	0	-3.721296	-4.120582	0.201171
41	1	0	-5.171753	-2.789826	-1.338967
42	1	0	-5.966656	-2.053791	0.047700
43	1	0	-5.805705	-1.140971	-1.461294
44	1	0	-4.200890	0.517941	-1.423274
45	1	0	-3.684054	2.791244	-1.242572
46	1	0	-2.181506	3.457097	-1.929738
47	1	0	-2.885892	1.960362	-2.609464

TS3

0 2

Standard orientation:

Center Number	Atomic Number	Atomic Type	Coordinates (Angstroms) X	Y	Z
1	7	0	3.877017	-1.838162	0.000150
2	6	0	3.584452	-3.184640	0.000245
3	8	0	4.434645	-4.063322	0.000383
4	7	0	2.219972	-3.552958	0.000184
5	6	0	1.127651	-2.708836	-0.000015
6	8	0	-0.029361	-3.114321	-0.000127
7	6	0	1.499896	-1.280757	-0.000070
8	7	0	0.540920	-0.350781	-0.000114
9	6	0	0.878518	0.984846	-0.000079
10	6	0	-0.126678	1.972978	-0.000009
11	6	0	0.184981	3.326273	0.000062
12	6	0	-0.918353	4.356727	0.000075
13	6	0	1.550398	3.717232	0.000063
14	6	0	1.933232	5.176036	0.000168
15	6	0	2.550454	2.742888	-0.000023
16	6	0	2.245160	1.373430	-0.000111
17	7	0	3.233792	0.393339	-0.000217
18	6	0	2.904992	-0.956327	-0.000022

19	6	0	4.647427	0.782670	-0.000365
20	1	0	2.027328	-4.548363	0.000229
21	1	0	-0.638731	-0.654840	-0.000259
22	1	0	-1.163168	1.647214	-0.000006
23	1	0	-0.862802	5.008244	0.880552
24	1	0	-1.899012	3.875633	0.000782
25	1	0	-0.863637	5.007318	-0.881151
26	1	0	1.532969	5.694632	-0.879306
27	1	0	1.533997	5.694216	0.880363
28	1	0	3.018664	5.302435	-0.000423
29	1	0	3.584229	3.064514	-0.000020
30	1	0	4.871347	1.373090	0.893237
31	1	0	5.242778	-0.126641	-0.000823
32	6	0	-5.830672	-1.998049	-0.000191
33	6	0	-4.469443	-1.722340	-0.000221
34	6	0	-4.035081	-0.374912	-0.000002
35	6	0	-4.983816	0.671869	0.000244
36	6	0	-6.343158	0.379037	0.000270
37	6	0	-6.771221	-0.955405	0.000053
38	1	0	-6.170573	-3.029718	-0.000345
39	1	0	-3.726621	-2.511541	-0.000392
40	1	0	-4.626916	1.697176	0.000414
41	1	0	-7.071879	1.184100	0.000459
42	1	0	-7.832868	-1.184217	0.000083
43	7	0	-2.700219	0.009714	-0.000002
44	8	0	-1.880137	-0.986285	-0.000286
45	1	0	4.870919	1.373789	-0.893601

TS$_4$

0 1

Standard orientation:

Center Number	Atomic Number	Atomic Type	Coordinates (Angstroms) X	Y	Z
1	1	0	-1.091236	-2.977769	-0.888260
2	6	0	3.012004	-3.089631	2.030470
3	6	0	1.821609	-3.045502	1.315827
4	6	0	1.798978	-2.471419	0.016469
5	6	0	3.007510	-1.962979	-0.529581
6	6	0	4.186130	-2.013563	0.203830
7	6	0	4.198575	-2.572788	1.488955

8	1	0	3.020689	-3.530099	3.023568
9	1	0	0.904932	-3.443428	1.732222
10	1	0	3.000744	-1.578619	-1.543865
11	1	0	5.105653	-1.632549	-0.231201
12	1	0	5.122606	-2.616018	2.057161
13	7	0	0.678995	-2.383588	-0.768619
14	8	0	-0.382932	-3.047936	-0.196635
15	7	0	-3.623460	0.726461	0.757970
16	6	0	-4.472561	-0.266628	0.334423
17	8	0	-5.638205	-0.374093	0.687690
18	7	0	-3.949960	-1.216288	-0.580326
19	6	0	-2.661191	-1.286933	-1.041312
20	8	0	-2.290887	-2.199362	-1.807231
21	6	0	-1.798988	-0.219039	-0.551972
22	7	0	-0.523223	-0.113723	-1.001689
23	6	0	0.236639	0.991683	-0.660527
24	6	0	1.513437	1.170612	-1.218284
25	6	0	2.303435	2.271522	-0.907109
26	6	0	3.668344	2.430701	-1.531126
27	6	0	1.794678	3.236262	-0.001173
28	6	0	2.611531	4.449273	0.369792
29	6	0	0.519636	3.069555	0.545489
30	6	0	-0.281796	1.961225	0.232203
31	7	0	-1.561650	1.791785	0.761548
32	6	0	-2.378823	0.750958	0.334873
33	6	0	-2.092210	2.775154	1.709031
34	1	0	-4.587249	-1.945207	-0.881611
35	1	0	0.033087	-1.038906	-1.200023
36	1	0	1.868121	0.430135	-1.926523
37	1	0	3.747232	3.365433	-2.098834
38	1	0	3.885936	1.606724	-2.215218
39	1	0	4.458310	2.451800	-0.770631
40	1	0	3.568142	4.164394	0.823989
41	1	0	2.848672	5.057758	-0.511271
42	1	0	2.078323	5.084553	1.081290
43	1	0	0.149568	3.824268	1.227920
44	1	0	-2.186334	3.754969	1.228927
45	1	0	-3.074417	2.435745	2.027183
46	1	0	-1.423117	2.854642	2.570706

TS_5

0 2

Standard orientation:

Center Number	Atomic Number	Atomic Type	Coordinates (Angstroms) X	Y	Z
1	1	0	-0.730566	-2.703706	-0.762680
2	6	0	-5.814358	-1.590624	-0.637719
3	6	0	-4.592834	-2.233311	-0.476684
4	6	0	-3.626727	-1.710755	0.412676
5	6	0	-3.915956	-0.523933	1.125743
6	6	0	-5.153419	0.096638	0.980349
7	6	0	-6.101848	-0.429091	0.093591
8	1	0	-6.552868	-1.996220	-1.322523
9	1	0	-4.354448	-3.141644	-1.020099
10	1	0	-3.171877	-0.113215	1.804112
11	1	0	-5.379789	0.993057	1.550057
12	1	0	-7.062509	0.062928	-0.025404
13	7	0	-2.451054	-2.423066	0.581435
14	8	0	-1.345649	-1.922572	-0.780473
15	1	0	-1.877617	-1.989654	1.309708
16	7	0	4.203158	-0.850493	0.176899
17	6	0	4.323901	-2.215317	0.116503
18	8	0	5.383796	-2.821617	0.210807
19	7	0	3.132835	-2.959422	-0.072852
20	6	0	1.858263	-2.461808	-0.184010
21	8	0	0.893321	-3.232294	-0.366576
22	6	0	1.774156	-1.007547	-0.083265
23	7	0	0.571769	-0.398035	-0.162973
24	6	0	0.528482	0.973213	-0.145714
25	6	0	-0.707228	1.639189	-0.283237
26	6	0	-0.810761	3.024379	-0.268897
27	6	0	-2.156248	3.690523	-0.432196
28	6	0	0.370511	3.793762	-0.103058
29	6	0	0.312443	5.301521	-0.078030
30	6	0	1.601568	3.146333	0.032943
31	6	0	1.710028	1.747554	0.010318
32	7	0	2.936099	1.093287	0.136235
33	6	0	3.013662	-0.294300	0.074488
34	6	0	4.162143	1.874042	0.313166
35	1	0	3.240033	-3.965405	-0.136992
36	1	0	-0.604559	-1.179651	-0.478965
37	1	0	-1.598437	1.032978	-0.417760

38	1	0	-2.409864	4.310288	0.436647
39	1	0	-2.948282	2.947830	-0.557220
40	1	0	-2.176335	4.350257	-1.308029
41	1	0	-0.096654	5.704322	-1.012806
42	1	0	-0.331447	5.667013	0.731294
43	1	0	1.306034	5.733995	0.064928
44	1	0	2.491049	3.752531	0.152196
45	1	0	4.088154	2.486422	1.217353
46	1	0	4.990494	1.176443	0.405757
47	1	0	4.323441	2.524580	-0.552654

TS_6

0 1

Standard orientation:

Center Number	Atomic Number	Atomic Type	Coordinates (Angstroms) X	Y	Z
1	6	0	0.165710	1.643084	2.649654
2	6	0	0.484425	2.118292	1.390584
3	6	0	-0.542291	2.394969	0.418837
4	6	0	-1.897439	2.105603	0.810218
5	6	0	-2.195917	1.632424	2.085110
6	6	0	-1.174229	1.393741	3.009817
7	1	0	0.956875	1.468365	3.374224
8	1	0	1.509670	2.344298	1.115991
9	1	0	-2.695228	2.318638	0.104159
10	1	0	-3.229454	1.439587	2.358507
11	1	0	-1.412459	1.024289	4.002521
12	7	0	-0.188223	2.828950	-0.797129
13	1	0	-1.035129	3.050186	-1.331111
14	7	0	-2.251351	-1.915876	0.250994
15	6	0	-3.465488	-1.529810	-0.273740
16	8	0	-4.535531	-2.025064	0.050822
17	7	0	-3.465520	-0.502900	-1.254498
18	6	0	-2.365715	0.187947	-1.707379
19	8	0	-2.410066	1.098102	-2.541037
20	6	0	-1.120001	-0.255765	-1.095890
21	7	0	0.033262	0.291253	-1.528160
22	6	0	1.241086	-0.072656	-0.967548
23	6	0	2.430818	0.562704	-1.352175
24	6	0	3.659933	0.177945	-0.832868

25	6	0	4.923007	0.880679	-1.267253
26	6	0	3.695922	-0.881882	0.112945
27	6	0	5.007083	-1.339220	0.702832
28	6	0	2.509675	-1.501878	0.506535
29	6	0	1.261089	-1.115339	-0.010142
30	7	0	0.071944	-1.736377	0.374595
31	6	0	-1.149401	-1.315984	-0.139393
32	6	0	0.098638	-2.837101	1.341759
33	1	0	-4.370987	-0.245932	-1.630621
34	1	0	-0.029466	1.191725	-2.020053
35	1	0	2.366739	1.383196	-2.060243
36	1	0	5.441649	1.341112	-0.417888
37	1	0	4.702798	1.669793	-1.990145
38	1	0	5.631082	0.185523	-1.734116
39	1	0	5.703380	-1.669156	-0.077223
40	1	0	5.505326	-0.528835	1.248607
41	1	0	4.861983	-2.170720	1.396827
42	1	0	2.562258	-2.302887	1.232877
43	1	0	0.529753	-2.496669	2.287629
44	1	0	-0.927120	-3.159824	1.502269
45	1	0	0.690905	-3.668856	0.947467

FMN

0 1

Standard orientation:

Center Number	Atomic Number	Atomic Type	Coordinates (Angstroms) X	Y	Z
1	6	0	-1.440151	-0.624000	-0.000007
2	6	0	-1.340311	0.835742	0.000223
3	6	0	-2.629791	1.602219	0.000178
4	7	0	-3.739037	0.780021	0.000503
5	6	0	-3.766153	-0.634970	-0.000243
6	7	0	-2.557888	-1.304285	-0.000448
7	7	0	-0.237592	-1.309228	0.000183
8	6	0	0.972121	-0.628734	0.000050
9	6	0	0.951308	0.790937	-0.000059
10	7	0	-0.221326	1.496519	0.000038
11	6	0	2.214635	-1.286566	0.000060
12	6	0	3.410962	-0.570565	-0.000055
13	6	0	3.395729	0.855024	-0.000071

14	6	0	2.170194	1.500649	-0.000091
15	6	0	-0.253786	-2.778489	0.000492
16	6	0	4.681288	1.646378	-0.000078
17	6	0	4.721237	-1.317462	-0.000162
18	8	0	-2.707999	2.818943	-0.000057
19	8	0	-4.847113	-1.202058	-0.000560
20	1	0	-4.645341	1.235640	0.000737
21	1	0	2.258072	-2.368710	0.000096
22	1	0	2.105267	2.584398	-0.000128
23	1	0	-1.292332	-3.098998	0.000925
24	1	0	0.251447	-3.154514	-0.893366
25	1	0	0.252190	-3.154058	0.894137
26	1	0	4.476655	2.719608	0.000364
27	1	0	5.294685	1.422155	0.880800
28	1	0	5.294369	1.422757	-0.881310
29	1	0	5.324348	-1.063676	0.879672
30	1	0	4.564276	-2.398733	-0.000576
31	1	0	5.324465	-1.063097	-0.879775

FMNH

0 2

Standard orientation:

Center Number	Atomic Number	Atomic Type	Coordinates (Angstroms) X	Y	Z
1	6	0	-1.461962	-0.631775	0.000006
2	6	0	-1.407873	0.787317	0.000047
3	6	0	-2.632063	1.560313	-0.000028
4	7	0	-3.760786	0.771588	0.000311
5	6	0	-3.791006	-0.649049	0.000164
6	7	0	-2.585644	-1.317507	0.000086
7	7	0	-0.246543	-1.314074	-0.000212
8	6	0	0.975204	-0.621344	-0.000213
9	6	0	0.988982	0.795451	-0.000140
10	7	0	-0.222491	1.457520	-0.000094
11	6	0	2.211128	-1.283310	-0.000107
12	6	0	3.427710	-0.591930	-0.000042
13	6	0	3.427562	0.823734	-0.000030
14	6	0	2.204234	1.490700	-0.000055
15	6	0	-0.251704	-2.777492	-0.000139
16	6	0	4.718438	1.606050	0.000291

17	6	0	4.724724	-1.364360	0.000112
18	8	0	-2.643495	2.797679	-0.000304
19	8	0	-4.879228	-1.208199	0.000150
20	1	0	-4.661487	1.236171	0.000269
21	1	0	-0.283605	2.472246	-0.000160
22	1	0	2.235607	-2.365797	0.000001
23	1	0	2.179931	2.577638	0.000027
24	1	0	-1.288874	-3.104189	-0.000095
25	1	0	0.254402	-3.157574	-0.893498
26	1	0	0.254386	-3.157483	0.893285
27	1	0	4.527130	2.682156	0.000745
28	1	0	5.329806	1.374969	0.880837
29	1	0	5.329833	1.375683	-0.880420
30	1	0	5.334522	-1.128103	0.880585
31	1	0	4.543370	-2.442111	-0.000018
32	1	0	5.334754	-1.127961	-0.880153

H_2O

0 1

Standard orientation:

Center Number	Atomic Number	Atomic Type	Coordinates (Angstroms) X	Y	Z
1	8	0	0.000000	0.000000	0.116492
2	1	0	0.000000	0.769501	-0.465966
3	1	0	0.000000	-0.769501	-0.465966

The reduction of nitrobenzene mediated by FMNH calculated at the M062X/6-31+G(d,p) theoretical level

RC

0 1

Standard orientation:

Center Number	Atomic Number	Atomic Type	Coordinates (Angstroms) X	Y	Z
1	6	0	0.000000	1.210640	-1.815237
2	6	0	0.000000	0.000000	-2.508148
3	6	0	0.000000	-1.210640	-1.815237

4	6	0	0.000000	-1.220271	-0.423578
5	6	0	0.000000	0.000000	0.240360
6	6	0	0.000000	1.220271	-0.423578
7	7	0	0.000000	0.000000	1.715780
8	8	0	0.000000	1.079380	2.279949
9	8	0	0.000000	-1.079380	2.279949
10	1	0	0.000000	2.149837	-2.357877
11	1	0	0.000000	0.000000	-3.593393
12	1	0	0.000000	-2.149837	-2.357877
13	1	0	0.000000	-2.141587	0.146002
14	1	0	0.000000	2.141587	0.146002

IC_1

0 2

Standard orientation:

Center Number	Atomic Number	Atomic Type	Coordinates (Angstroms) X	Y	Z
1	6	0	1.905048	-1.179491	0.000333
2	6	0	2.569184	0.049029	0.000410
3	6	0	1.832740	1.233809	0.000049
4	6	0	0.442696	1.208266	-0.000463
5	6	0	-0.203769	-0.033945	-0.000530
6	6	0	0.518034	-1.235002	-0.000164
7	7	0	-1.596261	-0.112396	-0.001569
8	8	0	-2.282070	-1.153212	0.000427
9	8	0	-2.259364	1.101743	0.000601
10	1	0	2.472914	-2.104231	0.000686
11	1	0	3.653444	0.081918	0.000783
12	1	0	2.343844	2.191178	0.000158
13	1	0	-0.136926	2.122400	-0.000720
14	1	0	-0.021028	-2.174885	-0.000182
15	1	0	-3.190548	0.826149	0.004225

IC_2

0 1

Standard orientation:

Center Number	Atomic Number	Atomic Type	Coordinates (Angstroms) X	Y	Z

1	6	0	-0.761151	-1.677843	0.000000
2	6	0	0.570810	-2.108098	0.000000
3	6	0	1.615472	-1.185679	0.000000
4	6	0	1.328642	0.177848	0.000000
5	6	0	0.000000	0.594021	0.000000
6	6	0	-1.055454	-0.320984	0.000000
7	7	0	-0.191833	2.027791	0.000000
8	8	0	-1.344255	2.382495	0.000000
9	1	0	-1.564944	-2.407101	0.000000
10	1	0	0.790792	-3.171203	0.000000
11	1	0	2.645104	-1.527406	0.000000
12	1	0	2.112424	0.929616	0.000000
13	1	0	-2.076423	0.046014	0.000000

IC_3

0 2

Standard orientation:

Center Number	Atomic Number	Atomic Type	Coordinates (Angstroms) X	Y	Z
1	6	0	1.278343	1.345355	-0.022381
2	6	0	2.212871	0.312419	0.056078
3	6	0	1.778424	-1.017288	0.052853
4	6	0	0.426530	-1.310565	-0.015106
5	6	0	-0.527234	-0.271646	-0.064437
6	6	0	-0.082077	1.067840	-0.086850
7	1	0	1.613927	2.377335	-0.051115
8	1	0	3.272407	0.540502	0.105864
9	1	0	2.501694	-1.824922	0.104040
10	1	0	0.064254	-2.333485	-0.016083
11	1	0	-0.776057	1.894170	-0.212750
12	8	0	-2.746758	0.290332	0.107981
13	1	0	-2.321376	1.071209	0.502748
14	7	0	-1.843132	-0.686308	-0.116785

IC_4

0 1

Standard orientation:

Center Number	Atomic Number	Atomic Type	Coordinates (Angstroms) X	Y	Z
1	6	0	-1.327858	1.349601	0.006662
2	6	0	-2.273885	0.329244	-0.029030
3	6	0	-1.842333	-0.998268	-0.028268
4	6	0	-0.486325	-1.298799	0.011917
5	6	0	0.458712	-0.266387	0.048903
6	6	0	0.036689	1.063026	0.044167
7	1	0	-1.649440	2.386773	0.000473
8	1	0	-3.333146	0.561218	-0.059526
9	1	0	-2.565967	-1.807188	-0.058584
10	1	0	-0.153280	-2.333647	0.024963
11	1	0	0.772932	1.857112	0.055526
12	8	0	2.688082	0.430071	-0.195937
13	1	0	3.211912	0.581164	0.599862
14	7	0	1.824460	-0.622821	0.162143
15	1	0	2.051115	-1.396760	-0.456323

IC_5

0 2

Standard orientation:

Center Number	Atomic Number	Atomic Type	Coordinates (Angstroms) X	Y	Z
1	6	0	1.078381	1.233237	-0.000099
2	6	0	1.804120	0.032823	0.000460
3	6	0	1.131650	-1.198030	0.000391
4	6	0	-0.248800	-1.236389	-0.000772
5	6	0	-1.015875	-0.029910	-0.000419
6	6	0	-0.302586	1.210952	-0.000272
7	1	0	1.605383	2.182185	-0.000045
8	1	0	2.889027	0.057779	-0.000091
9	1	0	1.699920	-2.122740	0.000424
10	1	0	-0.795610	-2.173681	-0.000509
11	1	0	-0.870322	2.138160	-0.000552
12	7	0	-2.349605	-0.136940	0.000508
13	1	0	-2.762502	0.800785	0.001488

PC

0 1

Standard orientation:

Center Number	Atomic Number	Atomic Type	Coordinates (Angstroms) X	Y	Z
1	6	0	1.169348	1.200690	0.003421
2	6	0	1.877991	-0.000015	0.008542
3	6	0	1.169325	-1.200686	0.003468
4	6	0	-0.221775	-1.205984	-0.005509
5	6	0	-0.935348	-0.000001	-0.008276
6	6	0	-0.221729	1.205983	-0.005441
7	1	0	1.703101	2.146472	0.006650
8	1	0	2.962514	-0.000031	0.016118
9	1	0	1.703060	-2.146480	0.006693
10	1	0	-0.764780	-2.147611	-0.016422
11	1	0	-0.764758	2.147606	-0.016156
12	7	0	-2.329945	-0.000028	-0.073008
13	1	0	-2.778209	-0.837944	0.268903
14	1	0	-2.778187	0.838261	0.268039

TS_1

0 2

Standard orientation:

Center Number	Atomic Number	Atomic Type	Coordinates (Angstroms) X	Y	Z
1	7	0	1.351957	3.359268	0.112303
2	6	0	0.123895	3.956392	-0.094133
3	8	0	-0.072431	5.145355	0.047980
4	7	0	-0.944275	3.134071	-0.502588
5	6	0	-0.909456	1.776442	-0.718256
6	8	0	-1.854373	1.147425	-1.170147
7	6	0	0.395652	1.174416	-0.392732
8	7	0	0.551926	-0.131575	-0.485907
9	6	0	1.781300	-0.699651	-0.268262
10	6	0	1.931954	-2.093048	-0.353349
11	6	0	3.157724	-2.698400	-0.144060
12	6	0	3.301182	-4.194490	-0.236287
13	6	0	4.275553	-1.885055	0.165061
14	6	0	5.622570	-2.513881	0.400485

15	6	0	4.130269	-0.504828	0.258912
16	6	0	2.889418	0.112165	0.050402
17	7	0	2.711357	1.487160	0.160109
18	6	0	1.477273	2.070484	-0.037633
19	6	0	3.850449	2.330388	0.512104
20	1	0	-1.816088	3.609207	-0.704386
21	1	0	-0.475119	-0.873816	-0.579427
22	1	0	1.051515	-2.686980	-0.584500
23	1	0	2.343465	-4.662542	-0.471539
24	1	0	3.661955	-4.618006	0.707111
25	1	0	4.018043	-4.479050	-1.013984
26	1	0	5.587942	-3.216230	1.240303
27	1	0	5.952786	-3.079712	-0.477312
28	1	0	6.378548	-1.757798	0.621196
29	1	0	5.000434	0.092711	0.503078
30	1	0	4.631409	2.228146	-0.246325
31	1	0	3.505306	3.359652	0.554591
32	6	0	-5.773937	-0.725683	1.235597
33	6	0	-4.396881	-0.578562	1.327511
34	6	0	-3.600028	-1.093609	0.299792
35	6	0	-4.150361	-1.740054	-0.810981
36	6	0	-5.531346	-1.874489	-0.880552
37	6	0	-6.344836	-1.372494	0.136715
38	1	0	-6.407096	-0.333241	2.024467
39	1	0	-3.925821	-0.079329	2.165469
40	1	0	-3.497140	-2.107273	-1.591882
41	1	0	-5.976312	-2.368808	-1.737804
42	1	0	-7.422598	-1.481618	0.072242
43	7	0	-2.197277	-0.949780	0.401660
44	8	0	-1.669941	-0.314649	1.316241
45	8	0	-1.472342	-1.546136	-0.502733
46	1	0	4.243289	2.028476	1.486946

TS$_2$

0 1

Standard orientation:

Center Number	Atomic Number	Atomic Type	Coordinates (Angstroms) X	Y	Z
1	1	0	0.836130	-2.394775	2.229766
2	6	0	3.625507	-3.155764	-1.866751

3	6	0	2.381710	-3.007483	-1.255145
4	6	0	2.228829	-2.050489	-0.253655
5	6	0	3.294262	-1.238959	0.141341
6	6	0	4.530406	-1.404739	-0.474612
7	6	0	4.700645	-2.357455	-1.481878
8	1	0	3.753745	-3.902955	-2.644064
9	1	0	1.527482	-3.616347	-1.526994
10	1	0	3.152522	-0.515759	0.938310
11	1	0	5.368523	-0.788743	-0.163352
12	1	0	5.667920	-2.477246	-1.959667
13	7	0	0.903059	-1.821169	0.277602
14	8	0	0.066491	-2.766624	0.146152
15	8	0	1.150416	-1.553310	1.859924
16	7	0	0.034993	3.528956	-0.631639
17	6	0	1.242911	3.970244	-0.130619
18	8	0	1.744512	5.030893	-0.427449
19	7	0	1.926271	3.132988	0.785667
20	6	0	1.513327	1.916099	1.256080
21	8	0	2.123896	1.248951	2.069690
22	6	0	0.221401	1.491096	0.677732
23	7	0	-0.267764	0.315928	1.003769
24	6	0	-1.503709	-0.085677	0.560737
25	6	0	-2.066588	-1.290810	1.011120
26	6	0	-3.306560	-1.706492	0.560955
27	6	0	-3.886192	-3.013362	1.029663
28	6	0	-3.999682	-0.898918	-0.369373
29	6	0	-5.341724	-1.337193	-0.887948
30	6	0	-3.451244	0.302379	-0.811886
31	6	0	-2.200727	0.732987	-0.357802
32	7	0	-1.630017	1.926169	-0.776321
33	6	0	-0.437029	2.373152	-0.258296
34	6	0	-2.328334	2.749586	-1.762433
35	1	0	2.803262	3.495814	1.142540
36	1	0	0.328382	-0.406600	1.636763
37	1	0	-1.510393	-1.889330	1.719814
38	1	0	-4.865164	-2.875251	1.500401
39	1	0	-3.221613	-3.492828	1.750026
40	1	0	-4.019917	-3.703786	0.190144
41	1	0	-5.263983	-2.306243	-1.392261
42	1	0	-6.058673	-1.458951	-0.069266
43	1	0	-5.750306	-0.614609	-1.596600
44	1	0	-4.012828	0.904910	-1.514900

45	1	0	-3.273772	3.105125	-1.343375
46	1	0	-1.691831	3.596153	-2.004035
47	1	0	-2.521064	2.152811	-2.656725

TS_3

0 2

Standard orientation:

Center Number	Atomic Number	Atomic Type	Coordinates (Angstroms) X	Y	Z
1	7	0	3.813975	-1.906106	0.008317
2	6	0	3.475188	-3.242800	0.005112
3	8	0	4.290054	-4.142055	0.009654
4	7	0	2.103089	-3.570046	-0.003815
5	6	0	1.043628	-2.694541	-0.009898
6	8	0	-0.123798	-3.045819	-0.017688
7	6	0	1.460737	-1.284679	-0.005752
8	7	0	0.527520	-0.340760	-0.010841
9	6	0	0.895573	0.984943	-0.007165
10	6	0	-0.085343	1.990402	-0.013571
11	6	0	0.260432	3.331103	-0.009738
12	6	0	-0.808049	4.392013	-0.015711
13	6	0	1.629124	3.685966	0.001827
14	6	0	2.033939	5.135242	0.005809
15	6	0	2.605438	2.693973	0.007680
16	6	0	2.265037	1.335785	0.002574
17	7	0	3.228116	0.333514	0.007148
18	6	0	2.873368	-1.000023	0.003409
19	6	0	4.642113	0.695952	0.015868
20	1	0	1.883738	-4.559355	-0.006063
21	1	0	-0.650881	-0.631873	-0.014545
22	1	0	-1.129108	1.687559	-0.020786
23	1	0	-0.754502	5.017207	0.882202
24	1	0	-1.800606	3.938705	-0.053233
25	1	0	-0.705627	5.057333	-0.879724
26	1	0	1.677183	5.645753	-0.895447
27	1	0	1.603281	5.662104	0.863978
28	1	0	3.119418	5.243211	0.049611
29	1	0	3.647094	2.991615	0.016386
30	1	0	4.867131	1.280215	0.912483
31	1	0	5.223226	-0.221943	0.016329

32	6	0	-5.804719	-1.936061	0.003480
33	6	0	-4.445146	-1.665413	-0.004022
34	6	0	-4.016157	-0.323627	0.000427
35	6	0	-4.950924	0.725323	0.012400
36	6	0	-6.308686	0.437305	0.019919
37	6	0	-6.737504	-0.892447	0.015444
38	1	0	-6.148402	-2.965586	0.000098
39	1	0	-3.701630	-2.454878	-0.013412
40	1	0	-4.585842	1.747944	0.015919
41	1	0	-7.034971	1.243547	0.029570
42	1	0	-7.799302	-1.117761	0.021297
43	7	0	-2.674688	0.049585	-0.005887
44	8	0	-1.883899	-0.940020	-0.014827
45	1	0	4.876568	1.285538	-0.874783

TS$_4$

0 1

Standard orientation:

Center Number	Atomic Number	Atomic Type	Coordinates (Angstroms) X	Y	Z
1	1	0	-1.962767	-2.763266	0.421660
2	6	0	2.265406	-0.717042	2.174088
3	6	0	1.037120	-1.191404	1.728952
4	6	0	0.993137	-2.133050	0.672802
5	6	0	2.210237	-2.593144	0.110348
6	6	0	3.422283	-2.124877	0.585235
7	6	0	3.459558	-1.166022	1.605814
8	1	0	2.293165	0.017447	2.974219
9	1	0	0.108694	-0.850186	2.174671
10	1	0	2.159416	-3.334125	-0.682625
11	1	0	4.348108	-2.500339	0.158768
12	1	0	4.411291	-0.793542	1.972370
13	7	0	-0.152579	-2.582278	0.081262
14	8	0	-1.244549	-2.279880	0.861306
15	7	0	-3.248240	1.442680	0.494545
16	6	0	-4.354834	0.657099	0.272754
17	8	0	-5.474310	0.940191	0.645366
18	7	0	-4.168291	-0.559045	-0.431165
19	6	0	-2.987571	-1.041017	-0.926162
20	8	0	-2.875310	-2.133204	-1.477421

21	6	0	-1.856762	-0.148914	-0.691609
22	7	0	-0.662493	-0.480593	-1.201942
23	6	0	0.426853	0.337273	-0.983195
24	6	0	1.685892	-0.016963	-1.482379
25	6	0	2.811771	0.736753	-1.201981
26	6	0	4.158479	0.321726	-1.729179
27	6	0	2.669376	1.896719	-0.406815
28	6	0	3.877474	2.725641	-0.062424
29	6	0	1.414130	2.277358	0.056301
30	6	0	0.273166	1.510228	-0.212050
31	7	0	-0.983458	1.867558	0.257732
32	6	0	-2.081181	1.060267	0.042673
33	6	0	-1.141168	3.099598	1.024136
34	1	0	-4.997801	-1.127061	-0.560212
35	1	0	-0.471610	-1.533047	-1.247738
36	1	0	1.761297	-0.914772	-2.089134
37	1	0	4.560895	1.063954	-2.427576
38	1	0	4.090571	-0.634745	-2.252072
39	1	0	4.883199	0.209887	-0.915763
40	1	0	4.597340	2.137643	0.517564
41	1	0	4.393934	3.067958	-0.965408
42	1	0	3.601465	3.602553	0.526146
43	1	0	1.332732	3.174263	0.658020
44	1	0	-0.789497	3.947982	0.431253
45	1	0	-2.196852	3.217306	1.252704
46	1	0	-0.564986	3.035610	1.952278

TS$_5$

0 2

Standard orientation:

Center Number	Atomic Number	Atomic Type	Coordinates (Angstroms) X	Y	Z
1	1	0	0.583951	-2.850602	-2.276231
2	6	0	-3.557312	-2.295799	0.887005
3	6	0	-2.678194	-3.065496	0.146591
4	6	0	-1.286588	-2.951023	0.360415
5	6	0	-0.801988	-2.067981	1.351160
6	6	0	-1.694415	-1.316093	2.104525
7	6	0	-3.064512	-1.411593	1.858889
8	1	0	-4.626603	-2.372523	0.717536

9	1	0	-3.021662	-3.751647	-0.621334
10	1	0	0.270046	-1.991901	1.511998
11	1	0	-1.323880	-0.634503	2.863456
12	1	0	-3.756532	-0.801340	2.431464
13	7	0	-0.453146	-3.662902	-0.468477
14	8	0	-0.241767	-2.498691	-1.903649
15	1	0	0.525388	-3.508484	-0.189994
16	7	0	3.514221	1.308383	0.384103
17	6	0	4.411922	0.273522	0.309685
18	8	0	5.596123	0.373437	0.564220
19	7	0	3.917200	-0.994908	-0.083712
20	6	0	2.624669	-1.301219	-0.404541
21	8	0	2.276786	-2.451181	-0.707761
22	6	0	1.714228	-0.165904	-0.349030
23	7	0	0.431769	-0.364411	-0.669492
24	6	0	-0.433763	0.691289	-0.549785
25	6	0	-1.800895	0.500330	-0.817088
26	6	0	-2.722322	1.524141	-0.685845
27	6	0	-4.183483	1.274857	-0.951811
28	6	0	-2.269522	2.799367	-0.275845
29	6	0	-3.243833	3.937320	-0.125899
30	6	0	-0.918712	3.000363	-0.001031
31	6	0	0.014307	1.963177	-0.122930
32	7	0	1.367472	2.146316	0.159586
33	6	0	2.256699	1.094205	0.079177
34	6	0	1.843067	3.461691	0.571584
35	1	0	4.597775	-1.745222	-0.110571
36	1	0	0.047795	-1.607604	-1.407436
37	1	0	-2.122454	-0.493432	-1.122548
38	1	0	-4.786189	1.460210	-0.055015
39	1	0	-4.348797	0.240649	-1.261926
40	1	0	-4.567502	1.932172	-1.739365
41	1	0	-3.756603	4.146787	-1.071052
42	1	0	-4.018426	3.702711	0.612871
43	1	0	-2.737564	4.850526	0.193654
44	1	0	-0.595899	3.984947	0.316650
45	1	0	1.352666	3.760089	1.503167
46	1	0	2.916784	3.397110	0.725183
47	1	0	1.619837	4.195263	-0.208398

TS_6

0 1

Standard orientation:

Center Number	Atomic Number	Atomic Type	Coordinates (Angstroms) X	Y	Z
1	6	0	-0.098888	0.600247	2.543289
2	6	0	-0.475058	-0.625442	2.034255
3	6	0	0.505971	-1.570726	1.571522
4	6	0	1.878894	-1.154844	1.657587
5	6	0	2.238010	0.076375	2.197743
6	6	0	1.256726	0.972041	2.615580
7	1	0	-0.863582	1.288555	2.895106
8	1	0	-1.517616	-0.929678	2.004513
9	1	0	2.645266	-1.856312	1.334778
10	1	0	3.286900	0.353997	2.255031
11	1	0	1.537923	1.943112	3.010105
12	7	0	0.101794	-2.692371	0.972854
13	1	0	0.920131	-3.276560	0.775467
14	7	0	2.255951	1.586066	-0.716158
15	6	0	3.463351	0.935367	-0.862297
16	8	0	4.538886	1.489704	-0.769123
17	7	0	3.444435	-0.451704	-1.144203
18	6	0	2.332188	-1.250689	-1.184924
19	8	0	2.350616	-2.463125	-1.344940
20	6	0	1.084193	-0.509771	-0.990576
21	7	0	-0.061751	-1.184447	-1.110990
22	6	0	-1.255110	-0.540521	-0.847516
23	6	0	-2.446390	-1.274089	-0.786851
24	6	0	-3.653463	-0.659460	-0.510751
25	6	0	-4.925446	-1.461415	-0.436966
26	6	0	-3.668464	0.741121	-0.291541
27	6	0	-4.966679	1.440923	0.009083
28	6	0	-2.486036	1.467113	-0.341220
29	6	0	-1.254350	0.846364	-0.604991
30	7	0	-0.060875	1.556030	-0.610100
31	6	0	1.143360	0.905754	-0.771742
32	6	0	-0.070747	2.993430	-0.352596
33	1	0	4.349939	-0.895998	-1.241317
34	1	0	-0.019267	-2.190515	-0.798225
35	1	0	-2.392406	-2.348942	-0.936611
36	1	0	-5.656438	-1.115387	-1.175548
37	1	0	-5.394110	-1.371903	0.548751

38	1	0	-4.729578	-2.518862	-0.622758
39	1	0	-5.689258	1.297132	-0.801053
40	1	0	-5.424213	1.043807	0.921565
41	1	0	-4.815488	2.513592	0.143859
42	1	0	-2.521697	2.534194	-0.157554
43	1	0	0.958038	3.342353	-0.383031
44	1	0	-0.662582	3.501935	-1.118656
45	1	0	-0.497230	3.187164	0.635737

FMN

0 1

Standard orientation:

Center Number	Atomic Number	Atomic Type	Coordinates (Angstroms) X	Y	Z
1	6	0	1.435789	-0.630593	-0.000091
2	6	0	1.334994	0.832561	-0.000097
3	6	0	2.625783	1.597738	-0.000092
4	7	0	3.729886	0.777452	-0.000105
5	6	0	3.755191	-0.633920	0.000190
6	7	0	2.547634	-1.306868	0.000079
7	7	0	0.238675	-1.303719	-0.000192
8	6	0	-0.966998	-0.622104	-0.000091
9	6	0	-0.947861	0.788133	-0.000004
10	7	0	0.228487	1.494201	-0.000035
11	6	0	-2.205534	-1.283417	-0.000042
12	6	0	-3.394809	-0.567048	0.000076
13	6	0	-3.380657	0.853233	0.000051
14	6	0	-2.160789	1.499531	0.000039
15	6	0	0.241256	-2.766976	-0.000377
16	6	0	-4.669780	1.631699	0.000000
17	6	0	-4.707786	-1.302248	0.000277
18	8	0	2.696813	2.805666	-0.000085
19	8	0	4.827536	-1.196770	0.000431
20	1	0	4.637569	1.229403	0.000224
21	1	0	-2.248005	-2.366153	-0.000064
22	1	0	-2.094095	2.583710	0.000066
23	1	0	1.275195	-3.100051	-0.000681
24	1	0	-0.270116	-3.132211	0.893683
25	1	0	-0.270622	-3.131945	-0.894246
26	1	0	-4.472685	2.705202	-0.000230

27	1	0	-5.276384	1.398368	-0.881470
28	1	0	-5.276314	1.398700	0.881604
29	1	0	-5.304072	-1.040090	-0.880029
30	1	0	-4.556998	-2.383234	0.000422
31	1	0	-5.303843	-1.039870	0.880683

FMNH

0 2

Standard orientation:

Center Number	Atomic Number	Atomic Type	Coordinates (Angstroms) X	Y	Z
1	6	0	1.458403	-0.635903	-0.000041
2	6	0	1.401768	0.783105	-0.000105
3	6	0	2.626000	1.555100	0.000020
4	7	0	3.749180	0.768600	0.000043
5	6	0	3.779002	-0.648048	0.000033
6	7	0	2.575471	-1.319460	-0.000017
7	7	0	0.247642	-1.308403	-0.000078
8	6	0	-0.969042	-0.615599	-0.000046
9	6	0	-0.984645	0.794458	-0.000062
10	7	0	0.226722	1.456307	-0.000087
11	6	0	-2.200349	-1.279949	0.000025
12	6	0	-3.410569	-0.588707	0.000025
13	6	0	-3.412116	0.821073	-0.000049
14	6	0	-2.195218	1.489604	-0.000067
15	6	0	0.240632	-2.765985	-0.000075
16	6	0	-4.706977	1.589670	-0.000029
17	6	0	-4.710277	-1.349437	0.000189
18	8	0	2.632002	2.783493	0.000136
19	8	0	4.859869	-1.201538	0.000050
20	1	0	4.650865	1.230485	0.000186
21	1	0	0.291804	2.471190	0.000008
22	1	0	-2.223731	-2.363166	0.000057
23	1	0	-2.170692	2.576702	-0.000081
24	1	0	1.273819	-3.103244	-0.000257
25	1	0	-0.270474	-3.136175	0.893706
26	1	0	-0.270808	-3.136138	-0.893687
27	1	0	-4.524121	2.666051	-0.000068
28	1	0	-5.311191	1.349048	-0.881184
29	1	0	-5.311178	1.349086	0.881143

30	1	0	-5.313145	-1.104312	-0.880818
31	1	0	-4.535682	-2.427098	0.000598
32	1	0	-5.313222	-1.103676	0.880979

H_2O

0 1

Standard orientation:

Center Number	Atomic Number	Atomic Type	Coordinates (Angstroms) X	Y	Z
1	8	0	0.000000	0.000000	0.115559
2	1	0	0.000000	0.768774	-0.462236
3	1	0	0.000000	-0.768774	-0.462236

The reduction of 3-NBG mediated by FMNH calculated at the B3LYP/6-31+G(d,p) theoretical level

RC

0 1

Standard orientation:

Center Number	Atomic Number	Atomic Type	Coordinates (Angstroms) X	Y	Z
1	6	0	-0.426320	0.599263	0.000520
2	1	0	-0.342240	1.240722	0.885012
3	1	0	-0.350894	1.247081	-0.880118
4	8	0	0.652009	-0.344202	-0.007731
5	6	0	1.907577	0.128744	-0.000705
6	6	0	2.979207	-0.782911	-0.002045
7	6	0	4.249889	-0.184091	-0.002684
8	6	0	3.418821	1.874760	0.010479
9	7	0	2.131380	1.436040	0.004951
10	7	0	4.528146	1.122563	0.000391
11	7	0	3.581774	3.232654	0.062616
12	1	0	4.500015	3.597153	-0.138041
13	1	0	2.782279	3.807279	-0.151922
14	7	0	3.077100	-2.166336	-0.008841
15	7	0	5.130000	-1.239320	-0.010470
16	1	0	6.137133	-1.169680	-0.009177
17	6	0	4.364991	-2.396554	-0.013077
18	1	0	4.824138	-3.375678	-0.018071

19	6	0	-1.734679	-0.159313	0.003252
20	6	0	-1.796026	-1.558760	0.014479
21	6	0	-2.924625	0.575953	-0.004364
22	6	0	-3.031687	-2.215727	0.018711
23	1	0	-0.875012	-2.129969	0.019677
24	6	0	-4.142110	-0.100311	0.000270
25	1	0	-2.922687	1.660405	-0.013399
26	6	0	-4.222512	-1.493353	0.011740
27	1	0	-3.062611	-3.300660	0.027400
28	1	0	-5.190222	-1.978780	0.014850
29	7	0	-5.388185	0.687512	-0.007929
30	8	0	-5.292988	1.916602	-0.019668
31	8	0	-6.456092	0.073118	-0.002688

IC_1

0 2

Standard orientation:

Center Number	Atomic Number	Atomic Type	Coordinates (Angstroms) X	Y	Z
1	6	0	-0.390223	0.575381	0.229008
2	1	0	-0.229427	1.086146	1.185790
3	1	0	-0.376948	1.338144	-0.555839
4	8	0	0.685426	-0.354382	0.014914
5	6	0	1.938269	0.119531	0.021610
6	6	0	3.010576	-0.776808	-0.151820
7	6	0	4.279350	-0.174716	-0.139627
8	6	0	3.450768	1.858792	0.183565
9	7	0	2.165249	1.417279	0.185732
10	7	0	4.558329	1.122687	0.018878
11	7	0	3.616889	3.200613	0.406624
12	1	0	4.521560	3.592834	0.196060
13	1	0	2.807197	3.790805	0.297877
14	7	0	3.109086	-2.147587	-0.338535
15	7	0	5.158755	-1.213995	-0.326488
16	1	0	6.164519	-1.137589	-0.366269
17	6	0	4.394938	-2.366892	-0.437226
18	1	0	4.853481	-3.334358	-0.589964
19	6	0	-1.696743	-0.183597	0.229552
20	6	0	-1.763120	-1.546564	0.551244
21	6	0	-2.867966	0.521138	-0.055527

22	6	0	-3.003704	-2.195035	0.591918
23	1	0	-0.849927	-2.094355	0.752452
24	6	0	-4.106794	-0.142216	-0.001039
25	1	0	-2.833209	1.571305	-0.319466
26	6	0	-4.182816	-1.510225	0.323418
27	1	0	-3.048240	-3.252065	0.836281
28	1	0	-5.149294	-1.998033	0.351251
29	7	0	-5.293922	0.533180	-0.275182
30	8	0	-5.150176	1.899060	-0.596543
31	8	0	-6.455183	0.057928	-0.269058
32	1	0	-6.076155	2.158351	-0.750339

IC$_2$

0 1

Standard orientation:

Center Number	Atomic Number	Atomic Type	Coordinates (Angstroms) X	Y	Z
1	6	0	0.699422	0.734536	0.002524
2	1	0	0.583683	1.372963	-0.880649
3	1	0	0.589581	1.375912	0.884355
4	8	0	-0.331950	-0.261032	0.007329
5	6	0	-1.608758	0.148967	0.000614
6	6	0	-2.634224	-0.814713	-0.000731
7	6	0	-3.933044	-0.279930	0.000774
8	6	0	-3.205302	1.817584	-0.007234
9	7	0	-1.897743	1.443696	-0.002086
10	7	0	-4.275989	1.011387	0.000658
11	7	0	-3.436042	3.166119	-0.056616
12	1	0	-4.371257	3.482882	0.146823
13	1	0	-2.666717	3.778869	0.162723
14	7	0	-2.663372	-2.201352	0.002972
15	7	0	-4.759743	-1.377511	0.005986
16	1	0	-5.769072	-1.357796	0.004186
17	6	0	-3.938193	-2.495327	0.006248
18	1	0	-4.348138	-3.496064	0.008891
19	6	0	2.043833	0.042957	0.000067
20	6	0	2.178196	-1.350279	-0.007695
21	6	0	3.202263	0.827821	0.004796
22	6	0	3.448558	-1.950953	-0.011027
23	1	0	1.287305	-1.968045	-0.011056

24	6	0	4.462729	0.223151	0.001445
25	1	0	3.154716	1.914313	0.010933
26	6	0	4.599471	-1.174717	-0.006538
27	1	0	3.522791	-3.034417	-0.017083
28	1	0	5.592062	-1.611711	-0.008876
29	7	0	5.570012	1.139518	0.007218
30	8	0	6.678883	0.622972	0.004341

IC$_3$

0 2

Standard orientation:

Center Number	Atomic Number	Atomic Type	Coordinates (Angstroms) X	Y	Z
1	6	0	-0.631101	0.801554	0.306518
2	1	0	-0.396828	1.276238	1.266323
3	1	0	-0.571844	1.571815	-0.467541
4	8	0	0.356858	-0.219758	0.049671
5	6	0	1.644788	0.144735	0.027454
6	6	0	2.633244	-0.837680	-0.180800
7	6	0	3.948379	-0.345462	-0.198074
8	6	0	3.304620	1.747768	0.160822
9	7	0	1.986944	1.417129	0.194944
10	7	0	4.341061	0.922233	-0.038525
11	7	0	3.590336	3.069147	0.388296
12	1	0	4.518449	3.384427	0.151842
13	1	0	2.831146	3.726261	0.300565
14	7	0	2.610650	-2.210195	-0.379331
15	7	0	4.731624	-1.453418	-0.415611
16	1	0	5.738874	-1.461636	-0.481010
17	6	0	3.870165	-2.536634	-0.513797
18	1	0	4.240911	-3.538007	-0.685234
19	6	0	-1.991105	0.149957	0.329171
20	6	0	-2.200165	-1.067865	1.004343
21	6	0	-3.066777	0.780736	-0.282486
22	6	0	-3.480792	-1.632144	1.059311
23	1	0	-1.359434	-1.575949	1.464918
24	6	0	-4.371474	0.221820	-0.222097
25	1	0	-2.936911	1.714019	-0.823091
26	6	0	-4.568309	-1.005715	0.461788
27	1	0	-3.625559	-2.575873	1.577438

28	1	0	-5.558616	-1.441273	0.505427
29	7	0	-5.342307	0.942763	-0.859067
30	8	0	-6.567962	0.305667	-0.741487
31	1	0	-7.168357	0.892680	-1.222975

IC4

0 1

Standard orientation:

Center Number	Atomic Number	Atomic Type	Coordinates (Angstroms) X	Y	Z
1	6	0	-0.494168	-1.417124	0.008792
2	1	0	-0.202918	-2.063767	-0.825528
3	1	0	-0.223737	-1.936936	0.935386
4	8	0	0.246189	-0.186233	-0.063730
5	6	0	1.581744	-0.244371	-0.024926
6	6	0	2.319708	0.954782	-0.077493
7	6	0	3.712094	0.784779	-0.021801
8	6	0	3.563293	-1.426012	0.102475
9	7	0	2.204880	-1.413583	0.063956
10	7	0	4.384064	-0.367008	0.070453
11	7	0	4.143164	-2.667161	0.147681
12	1	0	5.118662	-2.708960	0.399205
13	1	0	3.553321	-3.442013	0.407600
14	7	0	1.982598	2.297600	-0.163415
15	7	0	4.220535	2.060549	-0.076852
16	1	0	5.199004	2.308169	-0.063149
17	6	0	3.134013	2.918864	-0.161341
18	1	0	3.265535	3.990789	-0.218368
19	6	0	-1.973206	-1.109839	-0.030574
20	6	0	-2.871618	-2.167423	-0.242750
21	6	0	-2.461852	0.181606	0.160278
22	6	0	-4.242906	-1.917356	-0.255611
23	1	0	-2.499504	-3.175813	-0.405278
24	6	0	-3.845717	0.426042	0.150129
25	1	0	-1.765123	0.999639	0.313929
26	6	0	-4.742202	-0.626859	-0.059404
27	1	0	-4.937508	-2.734358	-0.429794
28	1	0	-5.806814	-0.430689	-0.089163
29	7	0	-4.281863	1.742673	0.436681
30	8	0	-5.572479	2.030051	-0.107200

31	1	0	-6.087929	2.294542	0.667774
32	1	0	-3.651404	2.434829	0.038659

IC_5

0 2

Standard orientation:

Center Number	Atomic Number	Atomic Type	Coordinates (Angstroms) X	Y	Z
1	6	0	-1.027089	0.833960	0.238342
2	1	0	-0.829599	1.307941	1.207129
3	1	0	-0.921746	1.601905	-0.533143
4	8	0	-0.048070	-0.203625	0.028066
5	6	0	1.245539	0.145587	0.023307
6	6	0	2.224580	-0.852208	-0.148091
7	6	0	3.546506	-0.377894	-0.134664
8	6	0	2.921099	1.730816	0.163404
9	7	0	1.598727	1.416177	0.177920
10	7	0	3.951450	0.886590	0.017823
11	7	0	3.214825	3.064225	0.280017
12	1	0	4.170505	3.307324	0.490172
13	1	0	2.485487	3.670447	0.620890
14	7	0	2.188547	-2.227754	-0.321564
15	7	0	4.320165	-1.500392	-0.306374
16	1	0	5.328464	-1.522601	-0.349397
17	6	0	3.446888	-2.573374	-0.412180
18	1	0	3.808554	-3.582484	-0.555222
19	6	0	-2.398351	0.205078	0.204186
20	6	0	-2.640499	-1.036180	0.832200
21	6	0	-3.447821	0.871189	-0.408393
22	6	0	-3.931224	-1.588953	0.841347
23	1	0	-1.814907	-1.569774	1.291154
24	6	0	-4.782048	0.337798	-0.405273
25	1	0	-3.274472	1.820969	-0.911030
26	6	0	-4.988272	-0.923882	0.243565
27	1	0	-4.096400	-2.548436	1.322675
28	1	0	-5.993653	-1.332055	0.240543
29	7	0	-5.838232	0.936048	-0.972639
30	1	0	-5.541907	1.819871	-1.401076

PC

0 1

Standard orientation:

Center Number	Atomic Number	Atomic Type	Coordinates (Angstroms) X	Y	Z
1	6	0	-0.950288	-1.123817	-0.045999
2	1	0	-0.673539	-1.857096	-0.807923
3	1	0	-0.793295	-1.579166	0.936614
4	8	0	-0.069848	0.026736	-0.161857
5	6	0	1.244989	-0.180890	-0.041540
6	6	0	2.129437	0.912937	-0.146893
7	6	0	3.486089	0.582581	0.001611
8	6	0	3.060410	-1.574826	0.293828
9	7	0	1.718188	-1.404163	0.177506
10	7	0	4.006593	-0.628909	0.224151
11	7	0	3.480590	-2.869273	0.469593
12	1	0	4.428765	-3.002015	0.786090
13	1	0	2.787643	-3.539940	0.762919
14	7	0	1.968031	2.273675	-0.361986
15	7	0	4.152273	1.777530	-0.128317
16	1	0	5.152438	1.904509	-0.080638
17	6	0	3.186068	2.749636	-0.345153
18	1	0	3.452630	3.788749	-0.482388
19	6	0	-2.366664	-0.646371	-0.224781
20	6	0	-3.030859	-0.839254	-1.442023
21	6	0	-3.025316	-0.009631	0.832045
22	6	0	-4.346589	-0.390651	-1.586683
23	1	0	-2.524167	-1.332554	-2.266208
24	6	0	-4.344574	0.449138	0.692056
25	1	0	-2.502111	0.144616	1.773436
26	6	0	-5.001351	0.248039	-0.535957
27	1	0	-4.868671	-0.538578	-2.527819
28	1	0	-6.022789	0.598687	-0.662319
29	7	0	-4.974743	1.138959	1.731139
30	1	0	-5.985135	1.133942	1.718893
31	1	0	-4.597616	0.988876	2.656524

TS_1

0 2

Standard orientation:

Center Number	Atomic Number	Atomic Type	Coordinates (Angstroms) X	Y	Z
1	6	0	-5.549510	-0.959303	0.872107
2	6	0	-4.505382	-1.220052	-0.089725
3	6	0	-4.168097	-2.630986	-0.372309
4	7	0	-4.904379	-3.521734	0.380011
5	6	0	-5.922240	-3.214363	1.314885
6	7	0	-6.212246	-1.884378	1.526386
7	7	0	-5.852555	0.374340	1.111327
8	6	0	-5.190113	1.394206	0.433976
9	6	0	-4.163741	1.057415	-0.488375
10	7	0	-3.835526	-0.256907	-0.714041
11	6	0	-5.495288	2.749648	0.630778
12	6	0	-4.817132	3.758582	-0.056039
13	6	0	-3.795061	3.420924	-0.984641
14	6	0	-3.491084	2.082550	-1.183873
15	6	0	-6.896701	0.703935	2.087034
16	6	0	-3.048050	4.490458	-1.744126
17	6	0	-5.180713	5.200930	0.192541
18	8	0	-3.322938	-2.981282	-1.189448
19	8	0	-6.493466	-4.132634	1.884943
20	6	0	2.027192	-2.326271	-2.516205
21	6	0	3.117174	-1.929694	-1.736312
22	6	0	2.951151	-0.977103	-0.716175
23	6	0	1.692172	-0.426646	-0.490267
24	6	0	0.603430	-0.839714	-1.278519
25	6	0	0.759499	-1.792168	-2.298340
26	7	0	-0.662884	-0.268635	-1.028107
27	8	0	-1.637957	-0.593033	-1.856163
28	8	0	-0.873833	0.516349	-0.076756
29	1	0	-4.723074	-4.508112	0.230693
30	1	0	-2.702429	-0.481293	-1.313063
31	1	0	-6.272742	3.031967	1.329436
32	1	0	-2.718015	1.796010	-1.887482
33	1	0	-7.265152	-0.228215	2.507437
34	1	0	-6.478657	1.331516	2.879491
35	1	0	-7.715272	1.236266	1.593045
36	1	0	-2.311207	4.046505	-2.417464
37	1	0	-3.724521	5.108952	-2.346046
38	1	0	-2.514707	5.167104	-1.065613
39	1	0	-5.976902	5.288524	0.935887

40	1	0	-4.317605	5.773144	0.553280
41	1	0	-5.522445	5.691724	-0.726704
42	1	0	2.165745	-3.063737	-3.300766
43	1	0	4.102251	-2.343995	-1.923809
44	1	0	1.529812	0.316934	0.281416
45	1	0	-0.098098	-2.096882	-2.883728
46	6	0	4.106276	-0.576308	0.167481
47	1	0	4.332607	-1.357500	0.901316
48	8	0	5.263277	-0.376433	-0.674680
49	6	0	6.442810	-0.048265	-0.121983
50	6	0	6.684062	0.188993	1.249492
51	7	0	7.439037	0.056965	-0.995160
52	6	0	8.016772	0.533035	1.555978
53	7	0	5.905298	0.176565	2.405767
54	6	0	8.671775	0.390515	-0.555060
55	7	0	9.039870	0.651293	0.713127
56	7	0	8.024575	0.725294	2.917206
57	6	0	6.734140	0.496558	3.364437
58	7	0	9.653519	0.436128	-1.507721
59	1	0	8.829986	0.979707	3.470025
60	1	0	6.473470	0.582739	4.410410
61	1	0	10.521758	0.884256	-1.260258
62	1	0	9.359708	0.457036	-2.471679
63	1	0	3.881806	0.339853	0.718875

TS_2

0 1

Standard orientation:

Center Number	Atomic Number	Atomic Type	Coordinates (Angstroms) X	Y	Z
1	6	0	4.006104	1.814597	0.812849
2	6	0	3.607254	1.564256	-0.565939
3	6	0	3.197299	2.732073	-1.393400
4	7	0	2.953110	3.850507	-0.631894
5	6	0	3.172910	3.991483	0.757456
6	7	0	3.770537	2.939904	1.431394
7	7	0	4.631971	0.780873	1.477318
8	6	0	4.931935	-0.428906	0.835466
9	6	0	4.513676	-0.625761	-0.495207
10	7	0	3.845100	0.398541	-1.145866

11	6	0	5.623363	-1.471013	1.472116
12	6	0	5.895185	-2.676334	0.821237
13	6	0	5.467775	-2.864348	-0.518443
14	6	0	4.784761	-1.831232	-1.152849
15	6	0	5.011702	0.968583	2.882174
16	6	0	5.751756	-4.151647	-1.253860
17	6	0	6.646165	-3.764688	1.549368
18	8	0	3.121492	2.698167	-2.609766
19	8	0	2.869931	5.041739	1.299289
20	6	0	-2.314371	-0.661117	-0.341653
21	6	0	-2.308297	-2.059914	-0.256006
22	6	0	-1.100679	-2.764046	-0.317650
23	6	0	0.107460	-2.084931	-0.466604
24	6	0	0.092329	-0.689560	-0.557915
25	6	0	-1.104061	0.024308	-0.488778
26	7	0	1.374804	-0.024921	-0.674380
27	8	0	1.353248	1.199189	-0.373779
28	8	0	1.657997	-0.089206	-2.480102
29	1	0	2.610748	4.673927	-1.113875
30	1	0	5.958926	-1.348792	2.494375
31	1	0	4.450808	-1.944006	-2.180194
32	1	0	4.657437	1.946986	3.196298
33	1	0	4.549193	0.190194	3.495249
34	1	0	6.099807	0.919869	2.986814
35	1	0	5.314144	-5.015403	-0.739334
36	1	0	5.343123	-4.123495	-2.266979
37	1	0	6.828943	-4.341207	-1.333034
38	1	0	6.890784	-3.462343	2.570674
39	1	0	6.060054	-4.689728	1.604777
40	1	0	7.584618	-4.014545	1.039843
41	1	0	-3.245229	-2.590754	-0.133351
42	1	0	-1.106777	-3.847228	-0.239344
43	1	0	1.055739	-2.612946	-0.499074
44	1	0	-1.068067	1.107181	-0.554387
45	1	0	3.249668	0.184041	-2.004425
46	1	0	1.377600	-0.981335	-2.739968
47	6	0	-3.588886	0.150973	-0.300477
48	1	0	-3.777531	0.635511	-1.265273
49	8	0	-4.688980	-0.718400	0.010880
50	1	0	-3.525630	0.941702	0.454635
51	6	0	-5.913969	-0.183001	0.089766
52	6	0	-7.009471	-1.019058	0.380715

53	7	0	-6.092162	1.118137	-0.100501
54	6	0	-8.244822	-0.354884	0.450369
55	7	0	-7.156542	-2.376495	0.625537
56	6	0	-7.350476	1.623947	-0.008857
57	7	0	-8.474657	0.949174	0.270124
58	7	0	-9.153821	-1.342564	0.745836
59	6	0	-8.438834	-2.527902	0.836363
60	7	0	-7.469310	2.964522	-0.261622
61	1	0	-10.147978	-1.214335	0.864647
62	1	0	-8.928356	-3.465730	1.060922
63	1	0	-8.334000	3.409854	0.003508
64	1	0	-6.625591	3.515632	-0.247750

TS$_3$

0 2

Standard orientation:

Center Number	Atomic Number	Atomic Type	Coordinates (Angstroms) X	Y	Z
1	6	0	-4.746362	1.581869	-0.420271
2	6	0	-3.580693	1.360424	0.398151
3	6	0	-3.023879	2.516582	1.126706
4	7	0	-3.772511	3.661873	0.974734
5	6	0	-4.937047	3.821878	0.183384
6	7	0	-5.382443	2.726047	-0.521497
7	7	0	-5.211032	0.494299	-1.147324
8	6	0	-4.551213	-0.731745	-1.104034
9	6	0	-3.423218	-0.883444	-0.255487
10	7	0	-2.969099	0.177385	0.497182
11	6	0	-4.967270	-1.833820	-1.867546
12	6	0	-4.306030	-3.061434	-1.803922
13	6	0	-3.178516	-3.213198	-0.952345
14	6	0	-2.760000	-2.124568	-0.200247
15	6	0	-6.401779	0.654431	-1.988034
16	6	0	-2.441722	-4.527430	-0.861591
17	6	0	-4.793404	-4.215063	-2.644345
18	8	0	-1.993965	2.476738	1.796087
19	8	0	-5.481493	4.915790	0.155855
20	6	0	2.056116	-2.162696	2.084164
21	6	0	2.472938	-0.976314	1.452949
22	6	0	1.568437	0.093672	1.337946

23	6	0	0.270326	-0.010545	1.822576
24	6	0	-0.151099	-1.212016	2.443916
25	6	0	0.769332	-2.279041	2.586169
26	7	0	-1.406419	-1.446304	2.988406
27	8	0	-2.321314	-0.560192	2.739701
28	1	0	-3.446792	4.491784	1.457891
29	1	0	-2.419958	-0.095181	1.597305
30	1	0	-5.824003	-1.741645	-2.523065
31	1	0	-1.897406	-2.216913	0.449613
32	1	0	-6.770988	1.667643	-1.851928
33	1	0	-6.143197	0.494913	-3.039455
34	1	0	-7.167663	-0.066194	-1.686967
35	1	0	-1.606658	-4.458394	-0.160719
36	1	0	-3.100234	-5.334532	-0.519078
37	1	0	-2.039960	-4.834249	-1.834763
38	1	0	-4.013259	-4.567781	-3.329496
39	1	0	-5.077113	-5.071699	-2.021257
40	1	0	-5.663039	-3.932256	-3.242643
41	1	0	2.750683	-2.993204	2.184828
42	1	0	-0.407537	0.835113	1.759705
43	1	0	0.436844	-3.183386	3.086322
44	8	0	4.010798	0.224020	0.060515
45	6	0	5.206881	0.543030	-0.463095
46	6	0	6.419390	-0.149757	-0.253746
47	7	0	5.185080	1.618084	-1.242216
48	6	0	7.530155	0.407947	-0.920135
49	7	0	6.800933	-1.278187	0.470891
50	6	0	6.330433	2.035912	-1.824640
51	7	0	7.551707	1.482552	-1.704368
52	7	0	8.585902	-0.404498	-0.580411
53	6	0	8.084466	-1.391931	0.250471
54	7	0	6.219985	3.124502	-2.645660
55	1	0	9.539362	-0.289607	-0.891588
56	1	0	8.720496	-2.165211	0.659030
57	1	0	7.071093	3.590029	-2.919241
58	1	0	5.374467	3.669206	-2.578715
59	1	0	1.894711	1.012873	0.864641
60	6	0	3.899363	-0.879822	0.972931
61	1	0	4.207594	-1.806137	0.477642
62	1	0	4.582013	-0.723838	1.816973

TS_4

0 1

Standard orientation:

Center Number	Atomic Number	Atomic Type	Coordinates (Angstroms) X	Y	Z
1	1	0	2.462899	-2.358404	2.322164
2	6	0	-0.682281	-0.576762	1.790435
3	6	0	0.478036	-0.017493	2.387916
4	6	0	0.418175	1.306189	2.895751
5	6	0	-0.757856	2.037727	2.792012
6	6	0	-1.896971	1.478882	2.201529
7	1	0	-0.633621	-1.586711	1.400565
8	1	0	1.288953	1.714781	3.396903
9	1	0	-0.798804	3.048576	3.187409
10	1	0	-2.810927	2.056187	2.115016
11	7	0	1.674429	-0.673531	2.510131
12	8	0	1.562224	-1.991972	2.127552
13	7	0	4.390298	-2.204832	-2.036541
14	6	0	4.721167	-3.390442	-1.427786
15	8	0	5.078403	-4.396173	-2.024947
16	7	0	4.645925	-3.432949	-0.011849
17	6	0	4.220913	-2.426957	0.816638
18	8	0	4.138263	-2.587336	2.051108
19	6	0	3.880060	-1.193829	0.120617
20	7	0	3.541225	-0.085064	0.826666
21	6	0	3.364094	1.120937	0.170980
22	6	0	3.129422	2.296412	0.903172
23	6	0	2.948020	3.526052	0.280089
24	6	0	2.700846	4.767139	1.102502
25	6	0	3.005724	3.583877	-1.135172
26	6	0	2.808340	4.892183	-1.860145
27	6	0	3.256574	2.420092	-1.866721
28	6	0	3.443014	1.177831	-1.241999
29	7	0	3.707584	0.012233	-1.962378
30	6	0	4.005535	-1.178421	-1.309748
31	6	0	3.760237	0.058352	-3.425696
32	1	0	4.885402	-4.317245	0.422680
33	1	0	2.969927	-0.219787	1.747248
34	1	0	3.111476	2.227904	1.985182
35	1	0	2.710545	4.538049	2.170995
36	1	0	1.730327	5.219709	0.865850

37	1	0	3.463404	5.533171	0.918177
38	1	0	1.827367	5.328372	-1.636709
39	1	0	3.559727	5.633565	-1.562566
40	1	0	2.877883	4.760920	-2.942784
41	1	0	3.304865	2.490402	-2.945951
42	1	0	4.581558	0.702278	-3.757757
43	1	0	3.929628	-0.953092	-3.785506
44	1	0	2.814089	0.443938	-3.816335
45	6	0	-3.091523	-0.484517	1.124976
46	1	0	-3.732963	-0.886531	1.916916
47	1	0	-2.827750	-1.312601	0.462592
48	8	0	-3.826548	0.518725	0.390938
49	6	0	-4.998935	0.203287	-0.182415
50	6	0	-5.605273	-1.072375	-0.194409
51	7	0	-5.601003	1.225847	-0.782083
52	6	0	-6.843924	-1.123370	-0.866421
53	7	0	-5.272747	-2.328160	0.311223
54	6	0	-6.787007	1.026247	-1.396798
55	7	0	-7.479223	-0.125020	-1.475824
56	7	0	-7.250193	-2.431645	-0.753224
57	6	0	-6.267778	-3.098913	-0.040800
58	7	0	-7.322031	2.115709	-2.031274
59	1	0	-8.102729	-2.817159	-1.132169
60	1	0	-6.348539	-4.153613	0.183959
61	1	0	-8.291926	2.064738	-2.301114
62	1	0	-6.936479	3.017684	-1.799435
63	6	0	-1.857427	0.163007	1.705415

TS$_5$

0 2

Standard orientation:

Center Number	Atomic Number	Atomic Type	Coordinates (Angstroms) X	Y	Z
1	1	0	3.905103	2.529289	1.816283
2	6	0	-0.928041	4.619716	1.734300
3	6	0	0.459087	4.568845	1.665707
4	6	0	1.092115	3.754069	0.698371
5	6	0	0.290999	2.983722	-0.183797
6	6	0	-1.703210	3.865608	0.845409
7	1	0	-1.414584	5.248055	2.474112

8	1	0	1.085036	5.145078	2.339228
9	1	0	0.781242	2.343125	-0.912104
10	1	0	-2.787065	3.905226	0.905971
11	7	0	2.466202	3.747354	0.658938
12	8	0	2.966868	2.290848	1.761355
13	1	0	2.819378	3.219745	-0.150825
14	7	0	3.980855	-2.064082	-2.025825
15	6	0	4.674965	-1.168791	-2.799087
16	8	0	5.322603	-1.470377	-3.794205
17	7	0	4.623437	0.195721	-2.415074
18	6	0	3.957484	0.721530	-1.333846
19	8	0	3.952799	1.943664	-1.104537
20	6	0	3.280392	-0.276998	-0.514417
21	7	0	2.624360	0.117163	0.597013
22	6	0	1.922998	-0.819694	1.321305
23	6	0	1.202710	-0.430417	2.468650
24	6	0	0.446605	-1.331581	3.205511
25	6	0	-0.330911	-0.864661	4.412201
26	6	0	0.409757	-2.689919	2.792836
27	6	0	-0.405502	-3.704277	3.555983
28	6	0	1.133227	-3.089236	1.666971
29	6	0	1.894820	-2.180463	0.916378
30	7	0	2.623607	-2.572534	-0.209517
31	6	0	3.327147	-1.640495	-0.962335
32	6	0	2.631888	-3.978374	-0.618513
33	1	0	5.119506	0.846205	-3.013600
34	1	0	2.841669	1.294448	1.148945
35	1	0	1.247559	0.612820	2.764315
36	1	0	-1.408902	-1.010598	4.273039
37	1	0	-0.161149	0.198642	4.598162
38	1	0	-0.045095	-1.414731	5.316886
39	1	0	-0.080842	-3.778626	4.601048
40	1	0	-1.467299	-3.429905	3.573306
41	1	0	-0.322453	-4.697970	3.108024
42	1	0	1.094254	-4.130560	1.372143
43	1	0	1.618500	-4.302745	-0.877001
44	1	0	3.277131	-4.067574	-1.488729
45	1	0	3.015109	-4.601606	0.195368
46	6	0	-1.944731	2.236772	-1.072225
47	1	0	-1.330557	1.696136	-1.797133
48	1	0	-2.645444	2.867908	-1.628657
49	8	0	-2.704215	1.290162	-0.282127

50	6	0	-3.559152	0.483971	-0.926355
51	6	0	-4.332749	-0.432162	-0.185674
52	7	0	-3.678535	0.551758	-2.246732
53	6	0	-5.204730	-1.214634	-0.960830
54	7	0	-4.450709	-0.739824	1.162237
55	6	0	-4.558816	-0.278300	-2.866624
56	7	0	-5.361644	-1.181960	-2.287276
57	7	0	-5.859565	-2.010428	-0.051668
58	6	0	-5.363008	-1.676957	1.199647
59	7	0	-4.596634	-0.189613	-4.231466
60	1	0	-6.564312	-2.699521	-0.269686
61	1	0	-5.720882	-2.164985	2.096003
62	1	0	-5.366357	-0.634566	-4.706291
63	1	0	-4.166579	0.613808	-4.661210
64	6	0	-1.097851	3.043334	-0.122213

TS$_6$

0 1

Standard orientation:

Center Number	Atomic Number	Atomic Type	Coordinates (Angstroms) X	Y	Z
1	6	0	-0.182116	-1.900892	-0.056196
2	6	0	-0.973415	-1.549616	-1.134241
3	6	0	-0.760213	-0.308624	-1.831524
4	6	0	0.272741	0.551226	-1.318892
5	6	0	1.074080	0.179519	-0.238204
6	6	0	0.841066	-1.048133	0.399869
7	1	0	-0.341294	-2.854324	0.440656
8	1	0	-1.739073	-2.217135	-1.515532
9	1	0	0.462490	1.489417	-1.834725
10	1	0	1.471113	-1.342752	1.232576
11	7	0	-1.561807	0.009779	-2.855756
12	1	0	-1.225430	0.878115	-3.284664
13	7	0	-1.548739	2.364243	1.851706
14	6	0	-0.895077	3.457734	1.327266
15	8	0	-0.182995	4.204258	1.984933
16	7	0	-1.063388	3.728810	-0.056087
17	6	0	-1.788012	2.972973	-0.949647
18	8	0	-1.887790	3.226134	-2.153805
19	6	0	-2.432275	1.821090	-0.333178

20	7	0	-3.236710	1.055850	-1.097997
21	6	0	-3.851235	-0.063528	-0.573443
22	6	0	-4.608804	-0.914745	-1.391240
23	6	0	-5.267069	-2.022449	-0.873503
24	6	0	-6.074603	-2.920272	-1.778824
25	6	0	-5.157140	-2.289201	0.517967
26	6	0	-5.857159	-3.478416	1.127990
27	6	0	-4.391351	-1.449939	1.327567
28	6	0	-3.718487	-0.329397	0.810815
29	7	0	-2.955739	0.517365	1.616542
30	6	0	-2.274895	1.598055	1.067908
31	6	0	-2.847539	0.260012	3.055068
32	1	0	-0.585476	4.548988	-0.411831
33	1	0	-3.104572	1.110524	-2.116921
34	1	0	-4.655303	-0.695591	-2.453504
35	1	0	-5.706212	-3.952758	-1.756058
36	1	0	-6.031087	-2.573773	-2.814094
37	1	0	-7.129038	-2.951638	-1.479080
38	1	0	-5.520394	-4.418095	0.673955
39	1	0	-5.672014	-3.540066	2.203182
40	1	0	-6.941785	-3.425350	0.975724
41	1	0	-4.315173	-1.678884	2.382930
42	1	0	-2.398189	-0.722069	3.229818
43	1	0	-2.213361	1.033177	3.481827
44	1	0	-3.838522	0.296698	3.518136
45	6	0	2.185649	1.092414	0.226395
46	1	0	2.370355	0.991299	1.295739
47	8	0	3.417169	0.831204	-0.502786
48	1	0	1.959532	2.133575	-0.008869
49	6	0	4.325426	-0.006698	0.008168
50	6	0	5.491493	-0.279202	-0.735886
51	7	0	4.150272	-0.569809	1.200160
52	6	0	6.394183	-1.154779	-0.111366
53	7	0	5.968929	0.131623	-1.971707
54	6	0	5.112726	-1.395814	1.682253
55	7	0	6.262744	-1.735692	1.085880
56	7	0	7.437271	-1.271120	-0.997963
57	6	0	7.120444	-0.476516	-2.090712
58	7	0	4.847666	-1.968701	2.904317
59	1	0	8.265967	-1.831915	-0.865484
60	1	0	7.785962	-0.393538	-2.939158
61	1	0	5.636949	-2.378090	3.381629

62	1	0	4.159469	-1.503832	3.476877

The reduction of 3-NBG mediated by FMNH calculated at the M062X/6-31+G(d,p) theoretical level

RC

0 1

Standard orientation:

Center Number	Atomic Number	Atomic Type	Coordinates (Angstroms) X	Y	Z
1	6	0	-0.415536	0.590813	0.000971
2	1	0	-0.330944	1.230267	0.886715
3	1	0	-0.338362	1.236723	-0.880740
4	8	0	0.647306	-0.354164	-0.006507
5	6	0	1.893318	0.123164	-0.000220
6	6	0	2.967503	-0.781979	-0.000495
7	6	0	4.227457	-0.177262	-0.001669
8	6	0	3.389595	1.869536	0.008550
9	7	0	2.106493	1.425593	0.003915
10	7	0	4.497797	1.129631	-0.000193
11	7	0	3.540107	3.225034	0.057879
12	1	0	4.455025	3.593267	-0.146863
13	1	0	2.734759	3.788204	-0.161557
14	7	0	3.071088	-2.160327	-0.005680
15	7	0	5.110381	-1.222861	-0.008156
16	1	0	6.116155	-1.146229	-0.006910
17	6	0	4.354565	-2.379921	-0.009499
18	1	0	4.820365	-3.355799	-0.013592
19	6	0	-1.721895	-0.161974	0.002646
20	6	0	-1.782439	-1.556781	0.010051
21	6	0	-2.905625	0.576274	-0.002616
22	6	0	-3.015650	-2.210604	0.012770
23	1	0	-0.860064	-2.127064	0.013440
24	6	0	-4.115890	-0.100613	0.000351
25	1	0	-2.901886	1.661566	-0.008684
26	6	0	-4.202895	-1.487818	0.007959
27	1	0	-3.048537	-3.294851	0.018654
28	1	0	-5.173874	-1.967570	0.009985
29	7	0	-5.361982	0.686789	-0.005627
30	8	0	-5.264165	1.901428	-0.011357

31	8	0	-6.415001	0.074917	-0.004410

IC₁

0 2

Standard orientation:

Center Number	Atomic Number	Atomic Type	Coordinates (Angstroms) X	Y	Z
1	6	0	0.384473	0.549744	0.015478
2	1	0	0.308393	1.197597	-0.864826
3	1	0	0.310905	1.188907	0.902494
4	8	0	-0.694700	-0.378978	0.012627
5	6	0	-1.930901	0.116959	0.003359
6	6	0	-3.020572	-0.771584	-0.005042
7	6	0	-4.270882	-0.148525	-0.006259
8	6	0	-3.403232	1.884678	-0.003455
9	7	0	-2.126764	1.422827	0.004457
10	7	0	-4.522484	1.162815	-0.002595
11	7	0	-3.532875	3.244053	-0.048751
12	1	0	-4.441607	3.622118	0.166150
13	1	0	-2.720523	3.789290	0.190075
14	7	0	-3.144715	-2.148074	-0.006629
15	7	0	-5.169815	-1.180455	-0.008364
16	1	0	-6.174180	-1.088253	-0.013808
17	6	0	-4.431315	-2.348842	-0.009209
18	1	0	-4.911542	-3.317646	-0.011930
19	6	0	1.683065	-0.217007	0.010859
20	6	0	1.727566	-1.611281	0.013762
21	6	0	2.866522	0.520561	0.004026
22	6	0	2.962866	-2.265648	0.010050
23	1	0	0.803114	-2.176883	0.018252
24	6	0	4.089825	-0.154232	0.000323
25	1	0	2.847641	1.604965	0.000691
26	6	0	4.152546	-1.554565	0.003469
27	1	0	2.993628	-3.350411	0.012088
28	1	0	5.119267	-2.043365	0.000128
29	7	0	5.292145	0.553037	-0.006416
30	8	0	5.171148	1.932072	-0.009898
31	8	0	6.440763	0.068751	-0.017349
32	1	0	6.098999	2.217957	-0.028433

IC_2

0 1

Standard orientation:

Center Number	Atomic Number	Atomic Type	Coordinates (Angstroms) X	Y	Z
1	6	0	-0.687575	0.732147	0.000598
2	1	0	-0.571515	1.368459	0.885313
3	1	0	-0.576148	1.372125	-0.882097
4	8	0	0.328019	-0.263730	-0.003839
5	6	0	1.596073	0.147828	0.001421
6	6	0	2.621044	-0.813744	0.001764
7	6	0	3.911448	-0.278057	-0.001320
8	6	0	3.184792	1.810548	0.006599
9	7	0	1.879132	1.436904	0.003649
10	7	0	4.251653	1.012922	-0.002048
11	7	0	3.407667	3.156671	0.054322
12	1	0	4.340107	3.474579	-0.155776
13	1	0	2.633275	3.760367	-0.168716
14	7	0	2.649761	-2.195697	-0.001856
15	7	0	4.736929	-1.369736	-0.007178
16	1	0	5.745310	-1.347253	-0.006831
17	6	0	3.919430	-2.484426	-0.006538
18	1	0	4.331715	-3.484031	-0.009780
19	6	0	-2.028816	0.044252	0.001567
20	6	0	-2.158945	-1.344170	0.007031
21	6	0	-3.183475	0.829821	-0.002847
22	6	0	-3.425406	-1.943073	0.008391
23	1	0	-1.265492	-1.959142	0.010024
24	6	0	-4.434194	0.223439	-0.001487
25	1	0	-3.134150	1.916886	-0.007295
26	6	0	-4.574490	-1.167411	0.004129
27	1	0	-3.501371	-3.025721	0.012728
28	1	0	-5.567664	-1.603776	0.004917
29	7	0	-5.553795	1.136871	-0.007010
30	8	0	-6.637120	0.606109	-0.005846

IC_3

0 2

Standard orientation:

Center Number	Atomic Number	Atomic Type	Coordinates (Angstroms) X	Y	Z
1	6	0	0.504725	0.358944	0.051111
2	1	0	0.342980	1.022165	-0.804931
3	1	0	0.312785	0.941569	0.958880
4	8	0	-0.405952	-0.737728	-0.009877
5	6	0	-1.716497	-0.487059	-0.012640
6	6	0	-2.307211	0.794299	0.025265
7	6	0	-3.710305	0.779393	0.002497
8	6	0	-3.819728	-1.422357	-0.070897
9	7	0	-2.479434	-1.565575	-0.059599
10	7	0	-4.513141	-0.278451	-0.047493
11	7	0	-4.538532	-2.582984	-0.077006
12	1	0	-5.515381	-2.517095	-0.313251
13	1	0	-4.041226	-3.425005	-0.318257
14	7	0	-1.834376	2.101195	0.074300
15	7	0	-4.074076	2.099054	0.039069
16	1	0	-5.021158	2.445960	0.038597
17	6	0	-2.908156	2.835061	0.081551
18	1	0	-2.922240	3.915531	0.117269
19	6	0	1.912136	-0.182099	0.049754
20	6	0	2.187764	-1.548294	0.165345
21	6	0	2.962227	0.720971	-0.055071
22	6	0	3.514076	-1.994467	0.178065
23	1	0	1.367797	-2.253223	0.240665
24	6	0	4.298612	0.274887	-0.039474
25	1	0	2.779076	1.788616	-0.151517
26	6	0	4.572365	-1.106178	0.078589
27	1	0	3.715406	-3.057664	0.266869
28	1	0	5.599000	-1.450524	0.087139
29	7	0	5.247521	1.259929	-0.149834
30	8	0	6.493636	0.706976	-0.126698
31	1	0	7.085719	1.464910	-0.209486

IC_4

0 1

Standard orientation:

Center Number	Atomic Number	Atomic Type	Coordinates (Angstroms) X	Y	Z

1	6	0	-0.380180	-1.033531	0.003918
2	1	0	-0.039218	-1.594796	-0.872618
3	1	0	-0.035431	-1.572893	0.892972
4	8	0	0.192180	0.273863	-0.011832
5	6	0	1.519161	0.401290	-0.004129
6	6	0	2.447144	-0.662261	0.006522
7	6	0	3.789416	-0.252164	0.000866
8	6	0	3.274459	1.892314	-0.009046
9	7	0	1.948047	1.652125	-0.011967
10	7	0	4.262005	0.990017	-0.009229
11	7	0	3.636900	3.208245	0.027933
12	1	0	4.594014	3.427790	-0.196119
13	1	0	2.923372	3.883431	-0.194242
14	7	0	2.361417	-2.049796	0.013080
15	7	0	4.510070	-1.416474	0.004254
16	1	0	5.516541	-1.482481	0.005859
17	6	0	3.598467	-2.451856	0.012407
18	1	0	3.916024	-3.485345	0.017370
19	6	0	-1.882184	-0.906151	0.005669
20	6	0	-2.648821	-2.077088	-0.045921
21	6	0	-2.511151	0.329934	0.062593
22	6	0	-4.034859	-1.991316	-0.041549
23	1	0	-2.158253	-3.045878	-0.094000
24	6	0	-3.910523	0.406000	0.067289
25	1	0	-1.914082	1.235557	0.110248
26	6	0	-4.678981	-0.754355	0.013263
27	1	0	-4.630825	-2.897825	-0.087478
28	1	0	-5.759256	-0.683556	-0.001496
29	7	0	-4.491947	1.688709	0.199239
30	8	0	-5.838835	1.715315	-0.209981
31	1	0	-6.312672	2.016920	0.574239
32	1	0	-4.004106	2.370986	-0.374703

IC$_5$

0 2

Standard orientation:

Center Number	Atomic Number	Atomic Type	Coordinates (Angstroms) X	Y	Z
1	6	0	0.886187	0.439150	0.009435

2	1	0	0.685553	1.060076	-0.870080
3	1	0	0.687543	1.050975	0.895846
4	8	0	0.018658	-0.692144	0.005142
5	6	0	-1.301687	-0.489755	0.006193
6	6	0	-1.937506	0.770037	0.008830
7	6	0	-3.339530	0.702900	-0.002289
8	6	0	-3.368885	-1.502919	-0.003410
9	7	0	-2.023588	-1.596302	0.000300
10	7	0	-4.103140	-0.384437	-0.011489
11	7	0	-4.043972	-2.687751	0.034323
12	1	0	-5.025932	-2.666929	-0.188400
13	1	0	-3.518394	-3.520994	-0.175124
14	7	0	-1.513098	2.094540	0.013276
15	7	0	-3.751358	2.008738	-0.004541
16	1	0	-4.710638	2.320758	-0.007900
17	6	0	-2.613531	2.788133	0.005858
18	1	0	-2.667570	3.867936	0.007435
19	6	0	2.314713	-0.046197	0.005159
20	6	0	2.639188	-1.410997	0.015856
21	6	0	3.328938	0.896471	-0.008221
22	6	0	3.982808	-1.817864	0.013071
23	1	0	1.841021	-2.144474	0.026431
24	6	0	4.703905	0.511000	-0.010815
25	1	0	3.088047	1.958926	-0.016852
26	6	0	5.000336	-0.887639	0.000119
27	1	0	4.216321	-2.877842	0.021565
28	1	0	6.046007	-1.177242	-0.002181
29	7	0	5.730776	1.368687	-0.023377
30	1	0	5.369810	2.327723	-0.030042

PC

0 1

Standard orientation:

Center Number	Atomic Number	Atomic Type	Coordinates (Angstroms) X	Y	Z
1	6	0	0.849775	0.391119	0.022034
2	1	0	0.660352	1.025750	-0.850357
3	1	0	0.656152	0.995345	0.915144
4	8	0	-0.040952	-0.723776	0.001785
5	6	0	-1.354150	-0.497502	0.002638

6	6	0	-1.970482	0.773518	0.011893
7	6	0	-3.373106	0.730679	-0.001255
8	6	0	-3.440712	-1.473356	-0.015661
9	7	0	-2.098354	-1.590738	-0.010866
10	7	0	-4.156401	-0.342957	-0.017897
11	7	0	-4.137159	-2.647598	0.015001
12	1	0	-5.116011	-2.606877	-0.218676
13	1	0	-3.624324	-3.485563	-0.207332
14	7	0	-1.524028	2.090930	0.023979
15	7	0	-3.763240	2.043371	0.003220
16	1	0	-4.717170	2.370879	0.001124
17	6	0	-2.612214	2.803258	0.019239
18	1	0	-2.647955	3.883801	0.027054
19	6	0	2.269677	-0.114865	0.016509
20	6	0	2.580111	-1.473110	0.038574
21	6	0	3.289727	0.837161	-0.010221
22	6	0	3.921821	-1.858566	0.035702
23	1	0	1.785752	-2.209447	0.057485
24	6	0	4.632566	0.451332	-0.013920
25	1	0	3.039350	1.897104	-0.022653
26	6	0	4.941470	-0.916643	0.011130
27	1	0	4.175361	-2.914432	0.052775
28	1	0	5.981640	-1.231704	0.016223
29	7	0	5.649495	1.408940	0.015637
30	1	0	6.540735	1.096453	-0.342769
31	1	0	5.394650	2.317117	-0.345782

TS1

0 2

Standard orientation:

Center Number	Atomic Number	Atomic Type	Coordinates (Angstroms) X	Y	Z
1	6	0	-1.914630	0.451199	1.308643
2	6	0	-2.492885	-0.866706	1.209366
3	6	0	-1.673500	-2.016821	1.632957
4	7	0	-0.415922	-1.656381	2.051690
5	6	0	0.139962	-0.362441	2.019722
6	7	0	-0.682333	0.689675	1.677770
7	7	0	-2.724247	1.497553	0.935901
8	6	0	-4.018740	1.279552	0.475721

9	6	0	-4.473220	-0.042472	0.302570
10	7	0	-3.673232	-1.111775	0.654276
11	6	0	-4.876237	2.332027	0.128370
12	6	0	-6.139174	2.087372	-0.399084
13	6	0	-6.581452	0.756843	-0.599925
14	6	0	-5.740489	-0.282555	-0.245044
15	6	0	-2.201791	2.860572	0.987383
16	6	0	-7.941121	0.482297	-1.185316
17	6	0	-7.034957	3.240373	-0.764590
18	8	0	-2.063809	-3.170365	1.588279
19	8	0	1.315676	-0.226504	2.300658
20	6	0	0.946754	-3.574001	-0.591321
21	6	0	1.955317	-2.610635	-0.516413
22	6	0	1.675684	-1.270063	-0.801382
23	6	0	0.384471	-0.901934	-1.166288
24	6	0	-0.615592	-1.878512	-1.203132
25	6	0	-0.354657	-3.223554	-0.927193
26	7	0	-1.950694	-1.462283	-1.471558
27	8	0	-2.909963	-2.321763	-1.295441
28	8	0	-2.212340	-0.307443	-1.820237
29	1	0	0.225019	-2.405950	2.287651
30	1	0	-3.493385	-1.950305	-0.271206
31	1	0	-4.556008	3.359075	0.257134
32	1	0	-6.046589	-1.316772	-0.376493
33	1	0	-1.165858	2.811022	1.310533
34	1	0	-2.263675	3.310064	-0.006860
35	1	0	-2.784882	3.453130	1.697666
36	1	0	-8.116633	-0.591440	-1.272947
37	1	0	-8.736330	0.906505	-0.563117
38	1	0	-8.039672	0.923703	-2.182657
39	1	0	-6.554441	4.197301	-0.552549
40	1	0	-7.291957	3.216386	-1.828918
41	1	0	-7.976263	3.200259	-0.206171
42	1	0	1.176047	-4.611469	-0.371258
43	1	0	2.965459	-2.889865	-0.239154
44	1	0	0.131170	0.130986	-1.383151
45	1	0	-1.160590	-3.945870	-0.948832
46	6	0	2.716448	-0.190148	-0.662970
47	1	0	2.546874	0.368327	0.265491
48	8	0	3.998731	-0.817212	-0.619527
49	6	0	5.081487	-0.059981	-0.437253
50	6	0	5.099586	1.350747	-0.404623

51	7	0	6.205997	-0.739460	-0.288229
52	6	0	6.371354	1.902710	-0.186917
53	7	0	4.148094	2.354882	-0.537876
54	6	0	7.358471	-0.067414	-0.096439
55	7	0	7.522400	1.257945	-0.023635
56	7	0	6.168316	3.256706	-0.184850
57	6	0	4.820618	3.459334	-0.401016
58	7	0	8.482620	-0.838053	0.005010
59	1	0	6.882950	3.956061	-0.052687
60	1	0	4.398857	4.453710	-0.445578
61	1	0	9.305043	-0.395009	0.382149
62	1	0	8.341686	-1.818243	0.190431
63	1	0	2.669591	0.508330	-1.503810

TS_2

0 1

Standard orientation:

Center Number	Atomic Number	Atomic Type	Coordinates (Angstroms) X	Y	Z
1	6	0	-3.890508	1.865170	-0.661738
2	6	0	-3.233140	1.485357	0.609381
3	6	0	-2.535511	2.592956	1.360857
4	7	0	-2.291229	3.683354	0.574130
5	6	0	-2.736418	3.882198	-0.752251
6	7	0	-3.613511	2.960826	-1.298503
7	7	0	-4.780456	0.970833	-1.170692
8	6	0	-5.107336	-0.215799	-0.494430
9	6	0	-4.511601	-0.478229	0.744881
10	7	0	-3.627792	0.437911	1.304206
11	6	0	-6.010435	-1.147879	-1.018616
12	6	0	-6.313647	-2.323337	-0.337282
13	6	0	-5.707916	-2.585112	0.909512
14	6	0	-4.820516	-1.653183	1.431519
15	6	0	-5.415575	1.254820	-2.455767
16	6	0	-6.025573	-3.848563	1.665333
17	6	0	-7.286654	-3.308381	-0.931136
18	8	0	-2.228923	2.499042	2.525874
19	8	0	-2.374186	4.875581	-1.340747
20	6	0	2.243524	-0.737518	-0.256288
21	6	0	2.279369	-2.052838	-0.731382

22	6	0	1.096964	-2.768355	-0.913211
23	6	0	-0.132005	-2.182330	-0.619481
24	6	0	-0.151437	-0.871824	-0.154993
25	6	0	1.017447	-0.142276	0.030263
26	7	0	-1.457235	-0.314136	0.130362
27	8	0	-1.476147	0.944432	-0.090172
28	8	0	-1.424025	-0.409588	1.922785
29	1	0	-1.747797	4.439379	0.974048
30	1	0	-6.487111	-0.966003	-1.974885
31	1	0	-4.352859	-1.819492	2.398770
32	1	0	-5.032420	2.203873	-2.819969
33	1	0	-5.173950	0.459052	-3.164876
34	1	0	-6.499090	1.315063	-2.324436
35	1	0	-5.772928	-4.737472	1.077415
36	1	0	-5.468456	-3.893554	2.603221
37	1	0	-7.092991	-3.912002	1.902836
38	1	0	-7.676000	-2.949991	-1.886346
39	1	0	-6.811907	-4.280592	-1.102489
40	1	0	-8.136447	-3.479576	-0.261718
41	1	0	3.237466	-2.512307	-0.948079
42	1	0	1.137032	-3.787446	-1.284043
43	1	0	-1.069797	-2.713104	-0.752734
44	1	0	0.946987	0.872244	0.412538
45	1	0	-2.723362	0.022778	1.913820
46	1	0	-1.340403	-1.357274	2.106431
47	6	0	3.496411	0.073673	-0.044406
48	1	0	3.527224	0.488738	0.968570
49	8	0	4.624800	-0.772259	-0.247135
50	1	0	3.544145	0.913649	-0.746820
51	6	0	5.833108	-0.239050	-0.074330
52	6	0	6.966726	-1.047099	-0.272218
53	7	0	5.961205	1.027347	0.274609
54	6	0	8.183057	-0.389699	-0.073891
55	7	0	7.161369	-2.367728	-0.630941
56	6	0	7.212185	1.529321	0.438035
57	7	0	8.367125	0.885634	0.275284
58	7	0	9.133972	-1.343231	-0.320175
59	6	0	8.456458	-2.502701	-0.647373
60	7	0	7.270473	2.831584	0.843700
61	1	0	10.132027	-1.209537	-0.263086
62	1	0	8.985701	-3.414118	-0.888579
63	1	0	8.161144	3.295885	0.767803

64	1	0	6.432341	3.381460	0.747987

TS₃

0 2

Standard orientation:

Center Number	Atomic Number	Atomic Type	Coordinates (Angstroms) X	Y	Z
1	6	0	-4.658612	1.477519	-0.556362
2	6	0	-3.544106	1.338298	0.344567
3	6	0	-3.117613	2.521998	1.107411
4	7	0	-3.897131	3.623288	0.867148
5	6	0	-4.994214	3.709861	-0.022141
6	7	0	-5.336787	2.580276	-0.733064
7	7	0	-5.009368	0.350194	-1.268867
8	6	0	-4.288515	-0.832704	-1.147359
9	6	0	-3.206990	-0.896087	-0.240335
10	7	0	-2.869596	0.207736	0.508675
11	6	0	-4.599016	-1.975627	-1.894937
12	6	0	-3.870127	-3.152922	-1.753539
13	6	0	-2.783269	-3.211587	-0.850653
14	6	0	-2.467592	-2.082706	-0.114200
15	6	0	-6.142106	0.410397	-2.187971
16	6	0	-1.979453	-4.474763	-0.694449
17	6	0	-4.236070	-4.362038	-2.571719
18	8	0	-2.164132	2.520935	1.870972
19	8	0	-5.576336	4.769463	-0.120848
20	6	0	1.886441	-2.099479	2.056680
21	6	0	2.305708	-0.902543	1.458016
22	6	0	1.428462	0.186770	1.400666
23	6	0	0.143369	0.091470	1.916721
24	6	0	-0.277049	-1.119295	2.503008
25	6	0	0.613135	-2.207755	2.587009
26	7	0	-1.539767	-1.341903	3.046651
27	8	0	-2.404293	-0.438504	2.800658
28	1	0	-3.662826	4.475975	1.362438
29	1	0	-2.416859	0.006398	1.650119
30	1	0	-5.422935	-1.957246	-2.598190
31	1	0	-1.623969	-2.094520	0.568542
32	1	0	-6.580508	1.401760	-2.113738
33	1	0	-5.798685	0.231618	-3.210988

34	1	0	-6.879066	-0.347803	-1.909942
35	1	0	-1.177676	-4.334668	0.033520
36	1	0	-2.607142	-5.304260	-0.351452
37	1	0	-1.528098	-4.781029	-1.644240
38	1	0	-3.399372	-4.676490	-3.204794
39	1	0	-4.487502	-5.211308	-1.927491
40	1	0	-5.093029	-4.158401	-3.216578
41	1	0	2.569874	-2.943498	2.108934
42	1	0	-0.523688	0.950021	1.898238
43	1	0	0.268784	-3.121247	3.062766
44	8	0	3.844099	0.361288	0.152516
45	6	0	5.034665	0.653602	-0.380286
46	6	0	6.196497	-0.139406	-0.272942
47	7	0	5.067587	1.790789	-1.050884
48	6	0	7.319564	0.401688	-0.917759
49	7	0	6.515952	-1.352123	0.328286
50	6	0	6.223537	2.180446	-1.626835
51	7	0	7.398277	1.540036	-1.597809
52	7	0	8.320813	-0.504953	-0.693025
53	6	0	7.775828	-1.527473	0.055033
54	7	0	6.167231	3.342805	-2.336673
55	1	0	9.270078	-0.423182	-1.024288
56	1	0	8.366556	-2.377826	0.366178
57	1	0	7.040421	3.769711	-2.599736
58	1	0	5.361108	3.929714	-2.195569
59	1	0	1.764888	1.113996	0.950304
60	6	0	3.714001	-0.822276	0.933864
61	1	0	3.953028	-1.701272	0.325471
62	1	0	4.432581	-0.797238	1.762122

TS4

0 1

Standard orientation:

Center Number	Atomic Number	Atomic Type	Coordinates (Angstroms) X	Y	Z
1	1	0	-4.057875	-0.341084	-2.322784
2	6	0	-0.780636	1.103491	-1.786025
3	6	0	-2.006085	1.809476	-1.730661
4	6	0	-1.973788	3.215645	-1.552650
5	6	0	-0.767581	3.878706	-1.424478

6	6	0	0.438578	3.168029	-1.449741
7	1	0	-0.792640	0.029604	-1.948557
8	1	0	-2.917989	3.752876	-1.543011
9	1	0	-0.757503	4.958379	-1.305850
10	1	0	1.387254	3.689105	-1.349871
11	7	0	-3.240337	1.229754	-1.769326
12	8	0	-3.132710	-0.084224	-2.184345
13	7	0	-2.267764	-3.179454	0.539878
14	6	0	-3.395978	-3.745245	-0.001163
15	8	0	-3.537984	-4.936141	-0.188861
16	7	0	-4.461639	-2.881249	-0.363921
17	6	0	-4.485557	-1.520333	-0.239314
18	8	0	-5.416207	-0.815740	-0.620133
19	6	0	-3.268635	-0.976888	0.359878
20	7	0	-3.201760	0.338547	0.584412
21	6	0	-2.072425	0.885357	1.154142
22	6	0	-1.981316	2.267982	1.360923
23	6	0	-0.815486	2.846535	1.827991
24	6	0	-0.717623	4.337719	1.999014
25	6	0	0.290506	2.010461	2.111085
26	6	0	1.603514	2.604314	2.542284
27	6	0	0.186391	0.634100	1.962048
28	6	0	-0.981990	0.045985	1.467440
29	7	0	-1.077884	-1.323034	1.252105
30	6	0	-2.208903	-1.881247	0.706484
31	6	0	0.065495	-2.175221	1.575649
32	1	0	-5.274041	-3.329921	-0.771097
33	1	0	-3.726770	0.943599	-0.124381
34	1	0	-2.845243	2.880628	1.118906
35	1	0	0.082421	4.751054	1.374734
36	1	0	-0.494442	4.607660	3.037024
37	1	0	-1.652715	4.824389	1.712639
38	1	0	1.959714	3.325048	1.797874
39	1	0	1.509922	3.138688	3.494429
40	1	0	2.362029	1.825571	2.645050
41	1	0	1.066635	0.029527	2.143285
42	1	0	-0.190346	-3.195586	1.305352
43	1	0	0.944546	-1.835615	1.021370
44	1	0	0.275334	-2.113362	2.647466
45	6	0	1.723161	1.020912	-1.695260
46	1	0	2.544341	1.645932	-2.057477
47	1	0	1.648047	0.143985	-2.344303

48	8	0	2.025281	0.585586	-0.352763
49	6	0	3.105911	-0.163921	-0.144358
50	6	0	4.043054	-0.555708	-1.121356
51	7	0	3.261756	-0.556333	1.114989
52	6	0	5.088853	-1.353715	-0.630585
53	7	0	4.193700	-0.338441	-2.485677
54	6	0	4.320965	-1.329009	1.437220
55	7	0	5.282297	-1.765051	0.618964
56	7	0	5.870498	-1.613576	-1.722459
57	6	0	5.280892	-0.980233	-2.797608
58	7	0	4.385436	-1.732219	2.740964
59	1	0	6.709386	-2.174159	-1.723417
60	1	0	5.707913	-1.035300	-3.789391
61	1	0	5.257573	-2.129189	3.052690
62	1	0	3.828597	-1.224451	3.409035
63	6	0	0.426798	1.783503	-1.644359

TS_5

0 2

Standard orientation:

Center Number	Atomic Number	Atomic Type	Coordinates (Angstroms) X	Y	Z
1	1	0	-4.854666	-0.510652	1.282397
2	6	0	-1.983406	-3.975016	-1.400034
3	6	0	-3.231269	-3.454663	-1.102284
4	6	0	-3.432561	-2.058714	-1.157485
5	6	0	-2.368079	-1.216982	-1.534465
6	6	0	-1.106001	-1.739575	-1.783948
7	6	0	-0.914351	-3.120513	-1.712718
8	1	0	-1.815657	-5.046407	-1.355340
9	1	0	-4.061203	-4.084496	-0.798166
10	1	0	-2.535667	-0.143075	-1.563056
11	1	0	0.081226	-3.525641	-1.867700
12	7	0	-4.619236	-1.541250	-0.691677
13	8	0	-4.198174	-1.196418	1.075273
14	1	0	-4.639443	-0.530455	-0.881697
15	7	0	-0.757196	3.500934	0.721533
16	6	0	-1.860408	4.121089	0.198209
17	8	0	-1.904986	5.300973	-0.101259
18	7	0	-3.019094	3.334196	-0.011016

19	6	0	-3.139921	1.988737	0.204830
20	8	0	-4.187558	1.373697	-0.044363
21	6	0	-1.938270	1.363095	0.733458
22	7	0	-1.958423	0.043175	0.968818
23	6	0	-0.781336	-0.554335	1.342708
24	6	0	-0.705810	-1.956501	1.424867
25	6	0	0.483732	-2.608870	1.696606
26	6	0	0.547412	-4.112534	1.684494
27	6	0	1.648379	-1.840862	1.927834
28	6	0	2.972877	-2.516136	2.154350
29	6	0	1.577919	-0.451267	1.893348
30	6	0	0.384789	0.210065	1.582045
31	7	0	0.314052	1.598944	1.489027
32	6	0	-0.799363	2.208667	0.958256
33	6	0	1.465392	2.398772	1.880668
34	1	0	-3.820371	3.820739	-0.395504
35	1	0	-3.268189	-0.673078	1.025095
36	1	0	-1.612146	-2.523368	1.219843
37	1	0	1.232758	-4.464059	0.904126
38	1	0	-0.438907	-4.540325	1.489915
39	1	0	0.910891	-4.510859	2.637859
40	1	0	3.258143	-3.091463	1.265735
41	1	0	3.761996	-1.783434	2.337640
42	1	0	2.933775	-3.207704	3.002974
43	1	0	2.486557	0.116273	2.065344
44	1	0	2.325020	2.170412	1.243205
45	1	0	1.198064	3.447331	1.776904
46	1	0	1.723610	2.171072	2.919089
47	6	0	0.017937	-0.764349	-1.999882
48	1	0	-0.213140	0.165471	-1.475490
49	1	0	0.164866	-0.526207	-3.061392
50	8	0	1.223795	-1.338561	-1.487762
51	6	0	2.256458	-0.529213	-1.237095
52	6	0	2.227177	0.882915	-1.281073
53	7	0	3.373366	-1.156663	-0.908219
54	6	0	3.453540	1.494051	-0.977166
55	7	0	1.256959	1.846888	-1.532077
56	6	0	4.470433	-0.431250	-0.611870
57	7	0	4.593597	0.899667	-0.632670
58	7	0	3.208388	2.838272	-1.072515
59	6	0	1.873122	2.984306	-1.392280
60	7	0	5.565760	-1.150670	-0.213414

61	1	0	3.878255	3.572493	-0.897757
62	1	0	1.407393	3.956399	-1.485628
63	1	0	6.450132	-0.667130	-0.240459
64	1	0	5.564527	-2.129306	-0.455811

TS$_6$

0 1

Standard orientation:

Center Number	Atomic Number	Atomic Type	Coordinates (Angstroms) X	Y	Z
1	6	0	-0.396314	-1.536545	0.132565
2	6	0	-1.105078	-1.227317	-1.010228
3	6	0	-0.813032	-0.034773	-1.759026
4	6	0	0.236653	0.797481	-1.243363
5	6	0	0.968113	0.455742	-0.107197
6	6	0	0.631680	-0.699973	0.601275
7	1	0	-0.629368	-2.449681	0.674667
8	1	0	-1.870522	-1.892711	-1.399410
9	1	0	0.497194	1.696864	-1.800359
10	1	0	1.197959	-0.962706	1.488899
11	7	0	-1.594728	0.302792	-2.786174
12	1	0	-1.202559	1.132339	-3.242249
13	7	0	-1.079454	2.023914	1.952590
14	6	0	-0.380922	3.121374	1.497690
15	8	0	0.474425	3.688759	2.147598
16	7	0	-0.673393	3.610379	0.202752
17	6	0	-1.520681	3.028210	-0.705962
18	8	0	-1.691117	3.424303	-1.849972
19	6	0	-2.207129	1.848561	-0.177307
20	7	0	-3.126617	1.267139	-0.954090
21	6	0	-3.730848	0.101597	-0.524563
22	6	0	-4.549040	-0.623198	-1.399685
23	6	0	-5.161020	-1.797243	-1.002273
24	6	0	-6.029130	-2.573299	-1.956189
25	6	0	-4.947425	-2.260628	0.320192
26	6	0	-5.605387	-3.531333	0.787077
27	6	0	-4.126002	-1.548973	1.184372
28	6	0	-3.490105	-0.363335	0.782510
29	7	0	-2.636063	0.335274	1.625978
30	6	0	-1.936779	1.429646	1.164964

31	6	0	-2.393345	-0.153835	2.980003
32	1	0	-0.146207	4.422586	-0.096654
33	1	0	-2.922059	1.306895	-1.987273
34	1	0	-4.669352	-0.248949	-2.412555
35	1	0	-5.630492	-3.579337	-2.124828
36	1	0	-6.092578	-2.069576	-2.922335
37	1	0	-7.045311	-2.688613	-1.564653
38	1	0	-5.304492	-4.382485	0.167120
39	1	0	-5.342842	-3.755004	1.822794
40	1	0	-6.696010	-3.455873	0.720554
41	1	0	-3.968072	-1.932312	2.185177
42	1	0	-1.950870	-1.153526	2.938437
43	1	0	-1.701924	0.532502	3.461624
44	1	0	-3.335650	-0.188413	3.533388
45	6	0	2.102023	1.344802	0.343961
46	1	0	2.289473	1.245817	1.414481
47	8	0	3.300278	1.057424	-0.400409
48	1	0	1.896214	2.390796	0.110251
49	6	0	4.059981	0.037874	-0.010850
50	6	0	5.160949	-0.332620	-0.805977
51	7	0	3.799012	-0.615813	1.108967
52	6	0	5.898439	-1.409469	-0.309014
53	7	0	5.689906	0.130220	-1.995971
54	6	0	4.608564	-1.640068	1.468515
55	7	0	5.674750	-2.095707	0.815317
56	7	0	6.896642	-1.598312	-1.226063
57	6	0	6.714251	-0.643970	-2.209766
58	7	0	4.249779	-2.293796	2.621531
59	1	0	7.615902	-2.303646	-1.179035
60	1	0	7.378434	-0.579177	-3.060510
61	1	0	4.965037	-2.874538	3.031685
62	1	0	3.670080	-1.770010	3.258824

The reduction of 2-NBP mediated by FMNH calculated at the B3LYP/6-31+G(d,p) theoretical level

RC

0 1

Standard orientation:

Center Number	Atomic Number	Atomic Type	Coordinates (Angstroms) X	Y	Z

1	6	0	-1.169036	1.282750	-0.032434
2	6	0	-2.580736	1.273250	-0.022963
3	6	0	-2.650364	-0.902499	0.003311
4	6	0	-0.556835	0.006480	-0.016603
5	6	0	-1.778695	3.329083	-0.067558
6	1	0	-1.789170	4.410377	-0.087173
7	7	0	-3.451615	-2.170942	0.013684
8	7	0	-3.376503	0.202196	0.006475
9	7	0	-1.341568	-1.080002	-0.010770
10	7	0	-2.946856	2.595495	-0.046854
11	1	0	-3.894686	2.945634	-0.042574
12	7	0	-0.697918	2.588955	-0.060079
13	8	0	0.746036	-0.258026	-0.007067
14	6	0	1.718581	0.834636	-0.003399
15	1	0	1.559072	1.443920	-0.895981
16	1	0	1.538704	1.456258	0.876906
17	6	0	3.090241	0.221317	0.016584
18	6	0	3.745472	-0.089984	-1.182218
19	6	0	3.727105	-0.055104	1.233606
20	6	0	5.014691	-0.671497	-1.166044
21	1	0	3.258029	0.121109	-2.130623
22	6	0	4.996521	-0.636388	1.253490
23	1	0	3.225634	0.183577	2.168086
24	6	0	5.642054	-0.945018	0.052786
25	1	0	5.513258	-0.909041	-2.101152
26	1	0	5.480988	-0.846278	2.202536
27	1	0	6.630563	-1.394862	0.066773
28	8	0	-3.150468	-3.022631	-0.813752
29	8	0	-4.347463	-2.249687	0.847653

IC_1

0 2

Standard orientation:

Center Number	Atomic Number	Atomic Type	Coordinates (Angstroms) X	Y	Z
1	6	0	-1.134526	1.328097	-0.000136
2	6	0	-2.547316	1.331836	0.000181
3	6	0	-2.659178	-0.857432	-0.000033
4	6	0	-0.544986	0.039983	-0.000377
5	6	0	-1.719026	3.382999	0.000079

6	1	0	-1.716565	4.464462	0.000137
7	7	0	-3.441085	-2.027815	-0.000060
8	7	0	-3.361672	0.282120	0.000236
9	7	0	-1.333076	-1.033438	-0.000358
10	7	0	-2.896189	2.661739	0.000372
11	1	0	-3.840011	3.021365	0.000302
12	7	0	-0.647488	2.629483	-0.000210
13	8	0	0.761924	-0.238313	-0.000672
14	6	0	1.737705	0.845296	-0.000584
15	1	0	1.576293	1.464468	-0.886426
16	1	0	1.575863	1.464800	0.884925
17	6	0	3.106947	0.223381	-0.000172
18	6	0	3.752385	-0.074653	-1.207529
19	6	0	3.751818	-0.074356	1.207564
20	6	0	5.018834	-0.662915	-1.209312
21	1	0	3.259075	0.153031	-2.149065
22	6	0	5.018266	-0.662617	1.210082
23	1	0	3.258071	0.153564	2.148813
24	6	0	5.653912	-0.957361	0.000570
25	1	0	5.509454	-0.889010	-2.151569
26	1	0	5.508445	-0.888476	2.152625
27	1	0	6.640350	-1.412129	0.000861
28	8	0	-2.720419	-3.229207	-0.000130
29	8	0	-4.688726	-2.083744	0.000497
30	1	0	-3.445629	-3.880141	-0.000034

IC_2

0 1

Standard orientation:

Center Number	Atomic Number	Atomic Type	Coordinates (Angstroms) X	Y	Z
1	6	0	-1.424400	0.992262	0.000029
2	6	0	-2.834995	0.876280	0.000002
3	6	0	-2.758848	-1.314563	-0.000815
4	6	0	-0.723280	-0.237148	-0.000520
5	6	0	-2.184305	2.987890	0.000294
6	1	0	-2.271228	4.066078	0.000497
7	7	0	-3.368271	-2.663540	-0.001810
8	7	0	-3.549923	-0.241992	-0.000250
9	7	0	-1.425776	-1.374061	-0.001240

10	7	0	-3.294647	2.173602	0.000280
11	1	0	-4.265304	2.453889	0.000475
12	7	0	-1.050633	2.328376	0.000283
13	8	0	0.600030	-0.404064	-0.000506
14	6	0	1.484959	0.757197	-0.000099
15	1	0	1.271317	1.360048	-0.886128
16	1	0	1.271013	1.359682	0.886092
17	6	0	2.899876	0.249256	0.000045
18	6	0	3.566440	0.003799	-1.207661
19	6	0	3.565892	0.003120	1.207940
20	6	0	4.874852	-0.483656	-1.209501
21	1	0	3.056853	0.192065	-2.149232
22	6	0	4.874289	-0.484333	1.210109
23	1	0	3.055865	0.190871	2.149374
24	6	0	5.530689	-0.728418	0.000379
25	1	0	5.381563	-0.670591	-2.151754
26	1	0	5.380591	-0.671806	2.152476
27	1	0	6.549309	-1.105472	0.000518
28	8	0	-4.579060	-2.653633	0.002460

TS_1

0 2

Standard orientation:

Center Number	Atomic Number	Atomic Type	Coordinates (Angstroms) X	Y	Z
1	6	0	3.660206	0.529764	1.873889
2	6	0	2.410914	0.625079	2.526385
3	6	0	1.382686	-0.493996	0.951614
4	6	0	3.636052	-0.187410	0.651023
5	6	0	4.011812	1.638489	3.665354
6	1	0	4.473292	2.203460	4.463812
7	7	0	0.183250	-1.052440	0.391046
8	7	0	1.246231	0.132382	2.121430
9	7	0	2.481425	-0.685738	0.218927
10	7	0	2.666267	1.339137	3.674589
11	1	0	1.980677	1.600770	4.368502
12	7	0	4.642100	1.173990	2.613343
13	8	0	4.679675	-0.438628	-0.145430
14	6	0	5.996051	0.089024	0.190316
15	1	0	5.932840	1.179582	0.237729

16	1	0	6.288539	-0.280515	1.175850
17	6	0	6.953413	-0.365733	-0.876883
18	6	0	7.060753	0.340404	-2.082845
19	6	0	7.744011	-1.505533	-0.683358
20	6	0	7.939436	-0.087616	-3.079396
21	1	0	6.448985	1.224659	-2.241750
22	6	0	8.626764	-1.935389	-1.677064
23	1	0	7.666149	-2.059320	0.248859
24	6	0	8.724968	-1.226644	-2.877230
25	1	0	8.013276	0.466756	-4.010468
26	1	0	9.235425	-2.820226	-1.514782
27	1	0	9.411232	-1.558691	-3.650993
28	8	0	0.171299	-1.595890	-0.721144
29	8	0	-0.910455	-0.960932	1.119831
30	7	0	-4.530908	3.059914	-0.989776
31	6	0	-3.471767	3.940422	-1.021375
32	8	0	-3.586727	5.132084	-1.269902
33	7	0	-2.182605	3.423593	-0.748042
34	6	0	-1.856680	2.119456	-0.440027
35	8	0	-0.710278	1.755530	-0.196577
36	6	0	-3.028037	1.221474	-0.419254
37	7	0	-2.850680	-0.067312	-0.146361
38	6	0	-3.929789	-0.916980	-0.131983
39	6	0	-3.740999	-2.287049	0.141753
40	6	0	-4.804011	-3.178252	0.160228
41	6	0	-4.566087	-4.639303	0.455819
42	6	0	-6.111274	-2.688851	-0.112014
43	6	0	-7.295244	-3.623105	-0.108237
44	6	0	-6.304918	-1.334394	-0.391199
45	6	0	-5.234230	-0.427201	-0.406842
46	7	0	-5.404728	0.927417	-0.680330
47	6	0	-4.320206	1.795338	-0.707478
48	6	0	-6.745118	1.452880	-0.959017
49	1	0	-1.421252	4.092300	-0.779737
50	1	0	-1.779280	-0.510987	0.435050
51	1	0	-2.730141	-2.629004	0.336518
52	1	0	-5.119959	-4.967003	1.343835
53	1	0	-3.505659	-4.833193	0.632580
54	1	0	-4.889446	-5.276970	-0.375564
55	1	0	-7.178264	-4.419133	-0.853383
56	1	0	-7.411700	-4.116435	0.864162
57	1	0	-8.223497	-3.090296	-0.328749

58	1	0	-7.309611	-0.987752	-0.598004
59	1	0	-7.403025	1.264173	-0.105603
60	1	0	-6.654288	2.522839	-1.127580
61	1	0	-7.157112	0.971826	-1.851358

TS$_2$

0 1

Standard orientation:

Center Number	Atomic Number	Atomic Type	Coordinates (Angstroms) X	Y	Z
1	7	0	-6.488295	-0.056986	0.828884
2	6	0	-6.808964	-1.388121	0.972735
3	8	0	-7.887352	-1.789779	1.382948
4	7	0	-5.820503	-2.344979	0.618737
5	6	0	-4.559419	-2.090861	0.133686
6	8	0	-3.757654	-2.976282	-0.167421
7	6	0	-4.282282	-0.654615	0.004757
8	7	0	-3.088096	-0.252195	-0.439502
9	6	0	-2.784575	1.069346	-0.612663
10	6	0	-1.532820	1.462574	-1.127827
11	6	0	-1.209141	2.805540	-1.272392
12	6	0	0.145423	3.200720	-1.806892
13	6	0	-2.168493	3.784342	-0.904490
14	6	0	-1.856680	5.252087	-1.050188
15	6	0	-3.419295	3.395237	-0.409491
16	6	0	-3.755118	2.046223	-0.251921
17	7	0	-4.987615	1.630078	0.249929
18	6	0	-5.303046	0.282168	0.376070
19	6	0	-5.981679	2.631254	0.649828
20	1	0	-6.085263	-3.317266	0.732281
21	1	0	-2.314515	-0.983453	-0.618549
22	1	0	-0.825724	0.693299	-1.406953
23	1	0	0.062488	3.786295	-2.730515
24	1	0	0.752911	2.317943	-2.016311
25	1	0	0.695519	3.814746	-1.084327
26	1	0	-0.984856	5.533414	-0.447471
27	1	0	-1.616621	5.507979	-2.089031
28	1	0	-2.699156	5.872893	-0.735678
29	1	0	-4.132120	4.164915	-0.142996
30	1	0	-6.259181	3.246288	-0.211731

31	1	0	-6.855867	2.103216	1.021733
32	6	0	3.707844	-2.503393	-0.303192
33	6	0	2.613880	-3.385929	-0.453663
34	6	0	1.150553	-1.849090	0.128542
35	6	0	3.356588	-1.200291	0.111306
36	6	0	4.538566	-4.369659	-0.929938
37	1	0	5.215767	-5.158962	-1.227043
38	7	0	-0.210627	-1.518168	0.494474
39	7	0	1.331139	-3.122031	-0.242871
40	7	0	2.072336	-0.902647	0.314390
41	7	0	3.175566	-4.576178	-0.857128
42	1	0	2.670580	-5.428225	-1.053762
43	7	0	4.899926	-3.152759	-0.609529
44	8	0	4.195388	-0.176374	0.319020
45	6	0	5.621711	-0.374541	0.195641
46	1	0	5.924952	-1.171764	0.883830
47	1	0	5.863668	-0.705008	-0.817962
48	6	0	6.315221	0.924427	0.524791
49	6	0	5.828933	1.781670	1.521279
50	6	0	7.500655	1.258170	-0.142188
51	6	0	6.521396	2.950845	1.843508
52	1	0	4.901375	1.538166	2.029236
53	6	0	8.198823	2.423150	0.186861
54	1	0	7.880499	0.605648	-0.924808
55	6	0	7.709805	3.273631	1.181285
56	1	0	6.131853	3.610455	2.613821
57	1	0	9.116722	2.669000	-0.339479
58	1	0	8.246960	4.183036	1.434887
59	8	0	-0.483129	-0.338871	0.828771
60	8	0	-1.086872	-1.922721	-0.815911
61	1	0	-1.439413	-2.798180	-0.578710
62	1	0	-5.567746	3.269543	1.435934

The reduction of 2-NBP mediated by FMNH calculated at the M062X/6-31+G(d,p) theoretical level

RC

0 1

Standard orientation:

Center Number	Atomic Number	Atomic Type	Coordinates (Angstroms) X	Y	Z

1	6	0	-1.113572	1.280315	-0.046019
2	6	0	-2.518186	1.330701	-0.065130
3	6	0	-2.687163	-0.827634	0.032631
4	6	0	-0.566322	-0.020121	0.021298
5	6	0	-1.629841	3.338760	-0.155856
6	1	0	-1.592502	4.418113	-0.209512
7	7	0	-3.545111	-2.060961	0.071694
8	7	0	-3.359829	0.299159	-0.019519
9	7	0	-1.391126	-1.065111	0.052194
10	7	0	-2.824780	2.660774	-0.137384
11	1	0	-3.756421	3.049639	-0.164296
12	7	0	-0.585905	2.558841	-0.103012
13	8	0	0.719181	-0.329697	0.059733
14	6	0	1.697069	0.716378	0.010176
15	1	0	1.543491	1.302858	-0.902524
16	1	0	1.556770	1.386939	0.864252
17	6	0	3.067182	0.089959	0.028615
18	6	0	3.263123	-1.266003	-0.231813
19	6	0	4.170468	0.909089	0.282062
20	6	0	4.554217	-1.793760	-0.241557
21	1	0	2.407670	-1.905960	-0.418439
22	6	0	5.458101	0.381159	0.267049
23	1	0	4.020469	1.965525	0.493700
24	6	0	5.652974	-0.974776	0.004259
25	1	0	4.698486	-2.850994	-0.441210
26	1	0	6.308019	1.026194	0.467255
27	1	0	6.656064	-1.389383	-0.003537
28	8	0	-3.112607	-3.045668	-0.486474
29	8	0	-4.604460	-1.966666	0.655767

IC$_1$

0 2

Standard orientation:

Center Number	Atomic Number	Atomic Type	Coordinates (Angstroms) X	Y	Z
1	6	0	-1.073620	1.326252	0.000003
2	6	0	-2.477770	1.396746	-0.000074
3	6	0	-2.697442	-0.773348	0.000037
4	6	0	-0.554188	0.012381	0.000068

5	6	0	-1.555764	3.397018	-0.000109
6	1	0	-1.502328	4.476910	-0.000174
7	7	0	-3.530761	-1.905826	0.000021
8	7	0	-3.341342	0.388350	-0.000058
9	7	0	-1.386390	-1.018455	0.000084
10	7	0	-2.762065	2.735582	-0.000193
11	1	0	-3.687778	3.137928	-0.000015
12	7	0	-0.525124	2.599329	-0.000026
13	8	0	0.732710	-0.317843	0.000109
14	6	0	1.718774	0.716069	0.000047
15	1	0	1.581750	1.349380	-0.883393
16	1	0	1.581804	1.349439	0.883450
17	6	0	3.084874	0.078640	0.000029
18	6	0	3.262365	-1.304016	-0.000301
19	6	0	4.204911	0.915568	0.000318
20	6	0	4.550189	-1.841790	-0.000362
21	1	0	2.395098	-1.954493	-0.000506
22	6	0	5.487652	0.377304	0.000261
23	1	0	4.070171	1.995182	0.000582
24	6	0	5.663889	-1.006894	-0.000078
25	1	0	4.679434	-2.919724	-0.000630
26	1	0	6.349966	1.036761	0.000470
27	1	0	6.664007	-1.428851	-0.000117
28	8	0	-2.881938	-3.121164	0.000099
29	8	0	-4.771730	-1.892949	0.000055
30	1	0	-3.627903	-3.743323	0.000416

IC_2

0 1

Standard orientation:

Center Number	Atomic Number	Atomic Type	Coordinates (Angstroms) X	Y	Z
1	6	0	-1.400027	0.984211	0.000135
2	6	0	-2.804034	0.876926	-0.000010
3	6	0	-2.744358	-1.302703	-0.000696
4	6	0	-0.712200	-0.249532	-0.000302
5	6	0	-2.143972	2.976126	0.000751
6	1	0	-2.226438	4.054436	0.001084
7	7	0	-3.370831	-2.650001	-0.001324
8	7	0	-3.526820	-0.236346	-0.000364

9	7	0	-1.417748	-1.377608	-0.000806
10	7	0	-3.255523	2.170385	0.000280
11	1	0	-4.224395	2.454396	0.001001
12	7	0	-1.018921	2.315811	0.000605
13	8	0	0.603993	-0.415393	-0.000275
14	6	0	1.457597	0.748156	0.000016
15	1	0	1.238731	1.350111	-0.886700
16	1	0	1.238510	1.349792	0.886894
17	6	0	2.875266	0.254204	0.000086
18	6	0	3.535208	0.007157	-1.204994
19	6	0	3.534198	0.004676	1.205199
20	6	0	4.837519	-0.486211	-1.207000
21	1	0	3.022857	0.197793	-2.144621
22	6	0	4.836499	-0.488715	1.207269
23	1	0	3.021072	0.193369	2.144796
24	6	0	5.489392	-0.734414	0.000153
25	1	0	5.343240	-0.676070	-2.148479
26	1	0	5.341434	-0.680523	2.148776
27	1	0	6.505235	-1.117110	0.000190
28	8	0	-4.566228	-2.612255	0.000862

TS_1

0 2

Standard orientation:

Center Number	Atomic Number	Atomic Type	Coordinates (Angstroms) X	Y	Z
1	6	0	3.616216	-2.112338	0.742144
2	6	0	2.488990	-2.871046	1.104858
3	6	0	1.129110	-1.572160	0.011725
4	6	0	3.332148	-1.002777	-0.088677
5	6	0	4.340388	-3.692279	1.966662
6	1	0	4.972698	-4.358029	2.537826
7	7	0	-0.203765	-1.208375	-0.392760
8	7	0	1.224019	-2.654010	0.767234
9	7	0	2.079743	-0.758474	-0.438043
10	7	0	2.981666	-3.880687	1.888289
11	1	0	2.431095	-4.602232	2.329867
12	7	0	4.762312	-2.654267	1.298448
13	8	0	4.223935	-0.147082	-0.578388
14	6	0	5.601990	-0.312189	-0.239498

15	1	0	5.714548	-0.275722	0.849700
16	1	0	5.944286	-1.297606	-0.573529
17	6	0	6.399277	0.784225	-0.898031
18	6	0	5.803857	1.795134	-1.650900
19	6	0	7.787423	0.777066	-0.731834
20	6	0	6.593989	2.789271	-2.230020
21	1	0	4.727454	1.802107	-1.781450
22	6	0	8.572729	1.769232	-1.309072
23	1	0	8.255396	-0.011538	-0.146152
24	6	0	7.975738	2.781079	-2.062112
25	1	0	6.122016	3.573750	-2.813622
26	1	0	9.649611	1.753385	-1.172451
27	1	0	8.585841	3.556913	-2.513997
28	8	0	-0.417837	-0.192179	-1.042594
29	8	0	-1.166776	-1.983109	-0.003281
30	7	0	-2.997353	3.258272	1.220173
31	6	0	-1.752387	3.431349	1.794326
32	8	0	-1.345947	4.502442	2.193298
33	7	0	-0.932101	2.295680	1.930543
34	6	0	-1.234582	1.007182	1.559544
35	8	0	-0.522442	0.050661	1.828637
36	6	0	-2.523899	0.894922	0.855380
37	7	0	-2.904564	-0.266315	0.359238
38	6	0	-4.144352	-0.408560	-0.211375
39	6	0	-4.532777	-1.650152	-0.739182
40	6	0	-5.779947	-1.828966	-1.310102
41	6	0	-6.182711	-3.167187	-1.870236
42	6	0	-6.669713	-0.728290	-1.362046
43	6	0	-8.033921	-0.889807	-1.977436
44	6	0	-6.285093	0.506563	-0.849989
45	6	0	-5.022938	0.692021	-0.270081
46	7	0	-4.605748	1.919367	0.233839
47	6	0	-3.357302	2.079969	0.796879
48	6	0	-5.501700	3.069457	0.147626
49	1	0	-0.049108	2.441473	2.405537
50	1	0	-2.072432	-1.190834	0.244622
51	1	0	-3.821911	-2.470888	-0.688310
52	1	0	-7.081140	-3.552149	-1.376097
53	1	0	-5.384367	-3.899807	-1.738442
54	1	0	-6.405807	-3.098883	-2.940334
55	1	0	-7.960003	-1.201693	-3.024896
56	1	0	-8.614143	-1.657771	-1.454613

57	1	0	-8.596683	0.044801	-1.940581
58	1	0	-6.983452	1.332951	-0.907800
59	1	0	-6.426126	2.856068	0.691005
60	1	0	-4.996964	3.922187	0.593062
61	1	0	-5.730942	3.279010	-0.900791

TS_2

0 1

Standard orientation:

Center Number	Atomic Number	Atomic Type	Coordinates (Angstroms) X	Y	Z
1	7	0	0.156359	-1.236721	1.274791
2	6	0	-0.785551	-0.334163	1.697381
3	8	0	-1.958881	-0.604698	1.870547
4	7	0	-0.370512	0.991035	1.981955
5	6	0	0.854354	1.533890	1.704969
6	8	0	1.156441	2.683752	1.954519
7	6	0	1.762188	0.586842	0.982138
8	7	0	3.011749	0.985649	0.761721
9	6	0	3.943582	0.051251	0.324048
10	6	0	5.237539	0.465913	-0.000305
11	6	0	6.203212	-0.435544	-0.424612
12	6	0	7.588306	0.036530	-0.779852
13	6	0	5.866834	-1.803011	-0.515075
14	6	0	6.893518	-2.809914	-0.963134
15	6	0	4.579452	-2.219247	-0.189994
16	6	0	3.604363	-1.305764	0.222872
17	7	0	2.290389	-1.705003	0.519526
18	6	0	1.358626	-0.828697	0.964084
19	6	0	1.911962	-3.102898	0.318439
20	1	0	-1.070875	1.597058	2.394540
21	1	0	2.817854	1.953001	0.065260
22	1	0	5.468642	1.523932	0.088981
23	1	0	8.347138	-0.452355	-0.159377
24	1	0	7.680819	1.115495	-0.640678
25	1	0	7.828957	-0.191654	-1.823894
26	1	0	7.268731	-2.573413	-1.964723
27	1	0	7.758383	-2.822860	-0.290848
28	1	0	6.471725	-3.816861	-0.990211
29	1	0	4.340822	-3.273292	-0.270030

30	1	0	2.471146	-3.741820	1.007236
31	1	0	0.846676	-3.196293	0.507507
32	6	0	-2.854624	2.179704	-0.529186
33	6	0	-1.811721	3.117545	-0.482437
34	6	0	-0.311622	1.617340	-0.998950
35	6	0	-2.464549	0.865249	-0.877668
36	6	0	-3.759143	4.040703	-0.042502
37	1	0	-4.466320	4.820678	0.204142
38	7	0	1.059053	1.103164	-0.912793
39	7	0	-0.512624	2.887134	-0.689081
40	7	0	-1.179808	0.625176	-1.130107
41	7	0	-2.413509	4.304002	-0.170227
42	1	0	-1.944366	5.188720	-0.043025
43	7	0	-4.065224	2.791021	-0.247965
44	8	0	-3.258603	-0.181697	-0.979896
45	6	0	-4.559887	-0.127335	-0.355421
46	1	0	-4.433613	0.294399	0.647089
47	1	0	-5.215967	0.526414	-0.935954
48	6	0	-5.084460	-1.532987	-0.290349
49	6	0	-4.383466	-2.495572	0.443730
50	6	0	-6.267054	-1.881778	-0.939949
51	6	0	-4.871804	-3.796161	0.521485
52	1	0	-3.460868	-2.210596	0.944977
53	6	0	-6.758582	-3.184840	-0.855729
54	1	0	-6.806268	-1.133026	-1.514962
55	6	0	-6.059982	-4.142374	-0.125384
56	1	0	-4.327380	-4.543005	1.091293
57	1	0	-6.439052	-5.157749	-0.059039
58	8	0	1.472668	0.305733	-1.732741
59	8	0	2.094046	2.488989	-0.890924
60	1	0	1.543458	3.202051	-0.519128
61	1	0	2.135368	-3.381571	-0.713555
62	1	0	-7.680438	-3.450168	-1.363981

The reduction of ANBP mediated by FMNH calculated at the B3LYP/6-31+G(d,p) theoretical level

RC

0 1

Standard orientation:

Center Atomic Atomic Coordinates (Angstroms)

Number	Number	Type	X	Y	Z
1	6	0	-1.532932	1.281377	-0.128830
2	6	0	-2.940605	1.199897	-0.194997
3	6	0	-2.912201	-0.962256	0.053975
4	6	0	-0.864259	0.046634	0.054673
5	6	0	-2.233050	3.279702	-0.408213
6	1	0	-2.292094	4.351582	-0.539688
7	7	0	-3.654141	-2.262653	0.150816
8	7	0	-3.687374	0.097842	-0.097740
9	7	0	-1.598436	-1.072809	0.130440
10	7	0	-3.365389	2.492265	-0.375325
11	1	0	-4.327092	2.791306	-0.456492
12	7	0	-1.121822	2.601056	-0.265900
13	8	0	0.445109	-0.147417	0.166083
14	6	0	1.366249	0.986682	0.085640
15	1	0	1.234407	1.463858	-0.888866
16	1	0	1.103658	1.707581	0.862499
17	6	0	2.761136	0.457523	0.270160
18	6	0	3.421923	-0.148497	-0.803069
19	6	0	3.399171	0.568185	1.511207
20	6	0	4.720899	-0.661123	-0.655321
21	1	0	2.917730	-0.236717	-1.763080
22	6	0	4.694692	0.066526	1.663110
23	1	0	2.889704	1.038843	2.346764
24	6	0	5.352211	-0.542728	0.597150
25	1	0	5.198577	0.149599	2.621769
26	1	0	6.357546	-0.934744	0.729860
27	8	0	-3.272117	-3.173587	-0.573783
28	8	0	-4.588028	-2.306153	0.944705
29	7	0	5.352143	-1.320404	-1.709858
30	1	0	5.003132	-1.124495	-2.637373
31	1	0	6.360449	-1.368170	-1.671366

IC$_1$

0 2

Standard orientation:

Center Number	Atomic Number	Atomic Type	Coordinates (Angstroms) X	Y	Z
1	6	0	1.494200	1.331710	-0.058629

2	6	0	2.902922	1.273836	-0.146637
3	6	0	2.929881	-0.912134	0.010765
4	6	0	0.855809	0.073677	0.075735
5	6	0	2.157271	3.353929	-0.249092
6	1	0	2.197042	4.431669	-0.329629
7	7	0	3.664459	-2.112353	0.049743
8	7	0	3.674692	0.192703	-0.118607
9	7	0	1.601065	-1.030064	0.106924
10	7	0	3.302924	2.583768	-0.268138
11	1	0	4.258197	2.900595	-0.352475
12	7	0	1.059517	2.649915	-0.126266
13	8	0	-0.456928	-0.146651	0.179421
14	6	0	-1.388138	0.976066	0.156668
15	1	0	-1.143314	1.654558	0.976954
16	1	0	-1.260091	1.515582	-0.785253
17	6	0	-2.778802	0.419893	0.297347
18	6	0	-3.393223	0.360058	1.554122
19	6	0	-3.459531	-0.043650	-0.832494
20	6	0	-4.682616	-0.166575	1.663853
21	1	0	-2.868602	0.719313	2.434450
22	6	0	-4.754283	-0.577811	-0.729203
23	1	0	-2.974795	-0.000661	-1.805603
24	6	0	-5.360481	-0.632401	0.539057
25	1	0	-5.167132	-0.214582	2.634919
26	1	0	-6.361907	-1.044108	0.637841
27	8	0	2.899415	-3.278440	0.182232
28	8	0	4.906611	-2.222089	-0.021944
29	1	0	3.597814	-3.957947	0.191304
30	7	0	-5.404863	-1.093233	-1.852473
31	1	0	-6.412598	-1.144077	-1.798923
32	1	0	-5.080025	-0.757643	-2.748487

IC$_2$

0 1

Standard orientation:

Center Number	Atomic Number	Atomic Type	Coordinates (Angstroms) X	Y	Z
1	6	0	-1.778019	0.987874	-0.097152
2	6	0	-3.180356	0.810879	-0.169926
3	6	0	-3.026240	-1.356524	0.118255

4	6	0	-1.034301	-0.199465	0.106263
5	6	0	-2.607230	2.932244	-0.401525
6	1	0	-2.732593	3.996971	-0.545438
7	7	0	-3.585564	-2.720234	0.253257
8	7	0	-3.853650	-0.328665	-0.069761
9	7	0	-1.695127	-1.357131	0.208875
10	7	0	-3.685229	2.076207	-0.365764
11	1	0	-4.663216	2.311041	-0.460845
12	7	0	-1.453338	2.328671	-0.246317
13	8	0	0.289827	-0.306544	0.212531
14	6	0	1.132370	0.880776	0.099336
15	1	0	0.953665	1.338049	-0.877546
16	1	0	0.841723	1.595307	0.872461
17	6	0	2.562812	0.445290	0.260782
18	6	0	3.238398	-0.133643	-0.818196
19	6	0	3.219724	0.615673	1.485103
20	6	0	4.570504	-0.559313	-0.692977
21	1	0	2.720181	-0.268831	-1.765220
22	6	0	4.547803	0.200299	1.614787
23	1	0	2.699301	1.065468	2.325395
24	6	0	5.220130	-0.381948	0.542876
25	1	0	5.065794	0.330177	2.560691
26	1	0	6.251146	-0.707114	0.658313
27	8	0	-4.793750	-2.763241	0.183549
28	7	0	5.220335	-1.192677	-1.753165
29	1	0	4.839591	-1.029364	-2.674593
30	1	0	6.230220	-1.169480	-1.737500

TS_1

0 2

Standard orientation:

Center Number	Atomic Number	Atomic Type	Coordinates (Angstroms) X	Y	Z
1	6	0	3.414901	0.551020	2.175412
2	6	0	2.147165	0.663485	2.788759
3	6	0	1.149343	-0.424929	1.172685
4	6	0	3.415853	-0.154184	0.946178
5	6	0	3.730602	1.632321	3.989331
6	1	0	4.177187	2.179161	4.808597
7	7	0	-0.039596	-0.960719	0.570607

8	7	0	0.987301	0.195033	2.342755
9	7	0	2.267505	-0.628376	0.472660
10	7	0	2.379470	1.360323	3.952169
11	1	0	1.677645	1.626752	4.627794
12	7	0	4.385091	1.166704	2.952802
13	8	0	4.478674	-0.420819	0.182523
14	6	0	5.780510	0.107435	0.528802
15	1	0	5.710146	1.199960	0.579114
16	1	0	6.073629	-0.253043	1.518001
17	6	0	6.772037	-0.327606	-0.522533
18	6	0	8.111670	-0.514825	-0.156685
19	6	0	6.382936	-0.493918	-1.853219
20	6	0	9.048562	-0.857991	-1.134662
21	1	0	8.418928	-0.399312	0.878989
22	6	0	7.320390	-0.852888	-2.836455
23	1	0	5.340133	-0.362497	-2.126124
24	6	0	8.665011	-1.027669	-2.463613
25	1	0	10.088431	-1.006209	-0.857028
26	1	0	9.402290	-1.298383	-3.215386
27	8	0	-0.023883	-1.495941	-0.546022
28	8	0	-1.153119	-0.860505	1.266666
29	7	0	-4.712514	3.007106	-1.241623
30	6	0	-3.661573	3.898053	-1.261103
31	8	0	-3.770865	5.069145	-1.594656
32	7	0	-2.389096	3.417685	-0.869075
33	6	0	-2.071887	2.140294	-0.457559
34	8	0	-0.941445	1.808583	-0.114337
35	6	0	-3.233319	1.228742	-0.458086
36	7	0	-3.063204	-0.037705	-0.093211
37	6	0	-4.133647	-0.898086	-0.089765
38	6	0	-3.951435	-2.244936	0.285098
39	6	0	-5.006217	-3.145874	0.296119
40	6	0	-4.775868	-4.581766	0.700583
41	6	0	-6.297593	-2.691500	-0.089425
42	6	0	-7.471581	-3.638233	-0.097574
43	6	0	-6.484156	-1.360800	-0.469147
44	6	0	-5.421890	-0.443613	-0.478141
45	7	0	-5.585349	0.887320	-0.852915
46	6	0	-4.509419	1.766341	-0.865005
47	6	0	-6.908401	1.374850	-1.255978
48	1	0	-1.633900	4.093788	-0.891774
49	1	0	-2.009769	-0.443285	0.542694

50	1	0	-2.952005	-2.561293	0.564093
51	1	0	-5.393176	-4.862837	1.562209
52	1	0	-3.730153	-4.747886	0.969428
53	1	0	-5.027091	-5.274505	-0.111601
54	1	0	-7.298936	-4.482784	-0.775479
55	1	0	-7.648152	-4.064086	0.897426
56	1	0	-8.387604	-3.134137	-0.415230
57	1	0	-7.476648	-1.040505	-0.760212
58	1	0	-7.619863	1.241740	-0.435625
59	1	0	-6.814424	2.430593	-1.496908
60	1	0	-7.257159	0.824165	-2.134833
61	7	0	6.928212	-0.976798	-4.171675
62	1	0	7.523028	-1.547294	-4.756487
63	1	0	5.945867	-1.158162	-4.326616

TS_2

0 1

Standard orientation:

Center Number	Atomic Number	Atomic Type	Coordinates (Angstroms) X	Y	Z
1	7	0	-6.579231	0.094021	1.076352
2	6	0	-6.912851	-1.217201	1.331811
3	8	0	-7.960369	-1.564760	1.855680
4	7	0	-5.978026	-2.219005	0.957836
5	6	0	-4.757047	-2.027881	0.354863
6	8	0	-4.003096	-2.949916	0.042334
7	6	0	-4.461075	-0.609068	0.115348
8	7	0	-3.299544	-0.266090	-0.447677
9	6	0	-2.988161	1.034085	-0.734497
10	6	0	-1.777568	1.362187	-1.377394
11	6	0	-1.445988	2.683733	-1.646633
12	6	0	-0.136315	3.010266	-2.321103
13	6	0	-2.354672	3.707374	-1.271629
14	6	0	-2.033880	5.153849	-1.550630
15	6	0	-3.564139	3.382232	-0.645836
16	6	0	-3.907486	2.055212	-0.364091
17	7	0	-5.099402	1.703226	0.268080
18	6	0	-5.429443	0.374379	0.507122
19	6	0	-6.036012	2.752437	0.683036
20	1	0	-6.252761	-3.175387	1.153208

21	1	0	-2.550940	-1.026856	-0.632688
22	1	0	-1.109637	0.558730	-1.657592
23	1	0	-0.288825	3.537109	-3.270811
24	1	0	0.433408	2.101134	-2.524235
25	1	0	0.486983	3.655680	-1.691092
26	1	0	-1.106087	5.457649	-1.051263
27	1	0	-1.886546	5.330504	-2.622791
28	1	0	-2.834038	5.813958	-1.206991
29	1	0	-4.239295	4.184550	-0.377451
30	1	0	-6.383679	3.310409	-0.191894
31	1	0	-6.879947	2.272332	1.171538
32	6	0	3.437916	-2.722700	-0.411060
33	6	0	2.298527	-3.552939	-0.514248
34	6	0	0.924514	-1.923109	0.031343
35	6	0	3.160720	-1.389699	-0.036289
36	6	0	4.163173	-4.649325	-0.983521
37	1	0	4.794734	-5.481916	-1.262439
38	7	0	-0.410087	-1.509536	0.409615
39	7	0	1.034027	-3.216573	-0.295226
40	7	0	1.895514	-1.019577	0.172195
41	7	0	2.792603	-4.783554	-0.883318
42	1	0	2.242236	-5.615103	-1.042084
43	7	0	4.590377	-3.442133	-0.711805
44	8	0	4.052565	-0.405654	0.129739
45	6	0	5.467048	-0.675387	-0.015467
46	1	0	5.739410	-1.489958	0.664430
47	1	0	5.674681	-1.010929	-1.034915
48	6	0	6.231506	0.584260	0.308453
49	6	0	7.405640	0.875335	-0.397703
50	6	0	5.811079	1.428172	1.339416
51	6	0	8.151267	2.006421	-0.054188
52	1	0	7.732222	0.229266	-1.207640
53	6	0	6.550991	2.572575	1.678962
54	1	0	4.891498	1.203505	1.872210
55	6	0	7.734745	2.851608	0.972132
56	1	0	9.062346	2.238247	-0.598761
57	1	0	8.321840	3.729872	1.229302
58	8	0	-0.630152	-0.300748	0.661002
59	8	0	-1.350698	-1.975059	-0.845667
60	1	0	-1.678282	-2.844631	-0.557490
61	1	0	-5.539649	3.435799	1.378155
62	1	0	5.167189	3.334803	2.986604

63	1	0	6.493589	4.334613	2.729899
64	7	0	6.146430	3.385622	2.741220

The reduction of ANBP mediated by FMNH calculated at the M062X/6-31+G(d,p) theoretical level

RC

0 1

Standard orientation:

Center Number	Atomic Number	Atomic Type	Coordinates (Angstroms) X	Y	Z
1	6	0	1.509514	1.312484	-0.000553
2	6	0	2.912366	1.222130	-0.011353
3	6	0	2.864629	-0.943742	0.023241
4	6	0	0.834337	0.071467	0.022080
5	6	0	2.231193	3.311336	-0.026407
6	1	0	2.303384	4.390193	-0.038809
7	7	0	3.595237	-2.257469	0.044771
8	7	0	3.646836	0.110475	-0.008232
9	7	0	1.551534	-1.051136	0.042213
10	7	0	3.351927	2.516052	-0.026887
11	1	0	4.318021	2.810062	-0.040880
12	7	0	1.113903	2.639149	-0.011057
13	8	0	-0.475131	-0.109715	0.027067
14	6	0	-1.343376	1.029846	-0.014018
15	1	0	-1.135255	1.673505	0.846328
16	1	0	-1.131461	1.605804	-0.921480
17	6	0	-2.773336	0.555567	0.004168
18	6	0	-3.100270	-0.789964	-0.112564
19	6	0	-3.779104	1.518581	0.130856
20	6	0	-4.443345	-1.196844	-0.103733
21	1	0	-2.312620	-1.530542	-0.209667
22	6	0	-5.110303	1.114965	0.135134
23	1	0	-3.522500	2.569961	0.228090
24	6	0	-5.448218	-0.229764	0.019150
25	1	0	-5.898134	1.855390	0.234990
26	1	0	-6.490940	-0.535415	0.019958
27	8	0	3.082179	-3.158280	0.672259
28	8	0	4.642441	-2.305558	-0.566094
29	7	0	-4.766011	-2.540717	-0.272212
30	1	0	-4.035818	-3.198636	-0.040484

31	1	0	-5.669103	-2.822752	0.079927

IC₁

0 2

Standard orientation:

Center Number	Atomic Number	Atomic Type	Coordinates (Angstroms) X	Y	Z
1	6	0	-1.367049	1.316785	0.049160
2	6	0	-2.765758	1.459043	0.039649
3	6	0	-3.096218	-0.694095	-0.058193
4	6	0	-0.914697	-0.021638	-0.003677
5	6	0	-1.744566	3.408009	0.135324
6	1	0	-1.636813	4.482825	0.183635
7	7	0	-3.987051	-1.780871	-0.116995
8	7	0	-3.680087	0.497815	-0.012532
9	7	0	-1.799762	-1.006730	-0.056982
10	7	0	-2.982525	2.809225	0.095477
11	1	0	-3.886952	3.257187	0.106047
12	7	0	-0.755598	2.559762	0.109386
13	8	0	0.353071	-0.416712	-0.009262
14	6	0	1.387644	0.567390	0.077689
15	1	0	1.305319	1.261808	-0.764550
16	1	0	1.252874	1.146549	0.998386
17	6	0	2.721788	-0.132717	0.074172
18	6	0	2.842410	-1.494691	0.345158
19	6	0	3.857981	0.636410	-0.176469
20	6	0	4.114139	-2.067901	0.366678
21	1	0	1.957266	-2.092685	0.526433
22	6	0	5.132715	0.062598	-0.151115
23	1	0	3.755562	1.698967	-0.391462
24	6	0	5.250588	-1.306504	0.126964
25	1	0	4.220169	-3.128654	0.573315
26	1	0	6.235444	-1.765239	0.153636
27	8	0	-3.402861	-3.028502	-0.137546
28	8	0	-5.225655	-1.703018	-0.139036
29	1	0	-4.179353	-3.607505	-0.210523
30	7	0	6.270446	0.847543	-0.347764
31	1	0	7.086748	0.347111	-0.669340
32	1	0	6.121472	1.702144	-0.865009

IC_2

0 1

Standard orientation:

Center Number	Atomic Number	Atomic Type	Coordinates (Angstroms) X	Y	Z
1	6	0	1.685039	0.996897	0.031638
2	6	0	3.092413	1.027878	0.048751
3	6	0	3.248114	-1.142809	-0.093723
4	6	0	1.123257	-0.295600	-0.055709
5	6	0	2.227763	3.047954	0.164127
6	1	0	2.202754	4.127034	0.231098
7	7	0	4.006954	-2.417801	-0.175737
8	7	0	3.920820	-0.007303	-0.010125
9	7	0	1.935300	-1.347042	-0.120050
10	7	0	3.413609	2.356935	0.135317
11	1	0	4.349592	2.733729	0.169412
12	7	0	1.173616	2.281853	0.104266
13	8	0	-0.169767	-0.588237	-0.083973
14	6	0	-1.128183	0.469287	0.025929
15	1	0	-0.935578	1.029984	0.947570
16	1	0	-1.007836	1.160730	-0.814442
17	6	0	-2.509852	-0.131637	0.042986
18	6	0	-3.592588	0.721197	-0.170611
19	6	0	-2.721597	-1.484604	0.301899
20	6	0	-4.904893	0.240483	-0.118315
21	1	0	-3.418658	1.776532	-0.374967
22	6	0	-4.030739	-1.964835	0.349262
23	1	0	-1.878811	-2.148131	0.454019
24	6	0	-5.114349	-1.120379	0.147209
25	1	0	-4.208247	-3.017669	0.546738
26	1	0	-6.128774	-1.507360	0.193561
27	8	0	5.191341	-2.261013	-0.122753
28	7	0	-5.986381	1.108597	-0.273996
29	1	0	-5.791997	1.955823	-0.788236
30	1	0	-6.848778	0.673660	-0.569315

TS_1

0 2

Standard orientation:

Center Number	Atomic Number	Atomic Type	Coordinates (Angstroms) X	Y	Z
1	6	0	3.197875	1.224817	1.788651
2	6	0	1.970892	1.509726	2.413251
3	6	0	0.983049	-0.192320	1.489068
4	6	0	3.184523	0.071752	0.968880
5	6	0	3.522583	2.975206	2.950532
6	1	0	3.966055	3.839712	3.425135
7	7	0	-0.199814	-0.979407	1.257410
8	7	0	0.833349	0.835175	2.308204
9	7	0	2.062200	-0.617913	0.839888
10	7	0	2.207411	2.636101	3.156189
11	1	0	1.525549	3.118502	3.722617
12	7	0	4.151281	2.162465	2.146400
13	8	0	4.219024	-0.417071	0.293747
14	6	0	5.469572	0.276084	0.332793
15	1	0	5.325169	1.297153	-0.038044
16	1	0	5.819237	0.345479	1.367690
17	6	0	6.464888	-0.467275	-0.520189
18	6	0	7.813473	-0.110902	-0.425804
19	6	0	6.064477	-1.465565	-1.400508
20	6	0	8.747436	-0.761325	-1.226066
21	1	0	8.127647	0.664579	0.267470
22	6	0	7.006943	-2.125157	-2.202900
23	1	0	5.015390	-1.738025	-1.462304
24	6	0	8.355505	-1.761602	-2.110463
25	1	0	9.797126	-0.491391	-1.159078
26	1	0	9.091652	-2.261934	-2.733822
27	8	0	-0.181668	-1.973080	0.538645
28	8	0	-1.296515	-0.580495	1.823653
29	7	0	-4.128103	2.734566	-1.903986
30	6	0	-2.988379	3.504810	-2.001226
31	8	0	-2.948701	4.577450	-2.566823
32	7	0	-1.804289	3.010105	-1.408565
33	6	0	-1.658650	1.832264	-0.719106
34	8	0	-0.607614	1.465049	-0.217102
35	6	0	-2.905474	1.059311	-0.631283
36	7	0	-2.884039	-0.101130	0.000211
37	6	0	-4.025395	-0.862839	0.067932
38	6	0	-3.989174	-2.107784	0.717193

39	6	0	-5.118037	-2.903146	0.797985
40	6	0	-5.067553	-4.237401	1.493660
41	6	0	-6.320574	-2.443876	0.206614
42	6	0	-7.562764	-3.290569	0.275271
43	6	0	-6.358375	-1.214757	-0.444703
44	6	0	-5.219508	-0.403202	-0.527810
45	7	0	-5.226975	0.828308	-1.173477
46	6	0	-4.084439	1.595918	-1.265616
47	6	0	-6.463258	1.311323	-1.782173
48	1	0	-0.983263	3.595193	-1.511010
49	1	0	-2.034463	-0.334179	0.893602
50	1	0	-3.043325	-2.427928	1.146340
51	1	0	-5.775269	-4.278022	2.328668
52	1	0	-4.068192	-4.431243	1.887130
53	1	0	-5.327164	-5.052260	0.809454
54	1	0	-7.403089	-4.262046	-0.204814
55	1	0	-7.845491	-3.490159	1.314408
56	1	0	-8.403952	-2.800858	-0.218872
57	1	0	-7.291351	-0.890111	-0.889899
58	1	0	-7.239214	1.398154	-1.016784
59	1	0	-6.263316	2.284822	-2.221324
60	1	0	-6.789465	0.613445	-2.558389
61	7	0	6.590742	-3.089869	-3.118909
62	1	0	7.292840	-3.761015	-3.394679
63	1	0	5.700518	-3.522470	-2.917899

TS$_2$

0 1

Standard orientation:

Center Number	Atomic Number	Atomic Type	Coordinates (Angstroms) X	Y	Z
1	7	0	0.357841	-1.124892	1.364842
2	6	0	-0.516629	-0.146834	1.766466
3	8	0	-1.699926	-0.334826	1.972869
4	7	0	-0.011124	1.159709	1.986673
5	6	0	1.239797	1.609941	1.663901
6	8	0	1.623427	2.744913	1.863927
7	6	0	2.065392	0.579541	0.956300
8	7	0	3.331058	0.888047	0.688068
9	6	0	4.189913	-0.120327	0.266300

10	6	0	5.494339	0.198859	-0.118171
11	6	0	6.390395	-0.779018	-0.525833
12	6	0	7.787736	-0.408484	-0.948238
13	6	0	5.971886	-2.126626	-0.535486
14	6	0	6.922890	-3.214588	-0.960558
15	6	0	4.673350	-2.447535	-0.152183
16	6	0	3.767398	-1.457356	0.241498
17	7	0	2.441177	-1.760965	0.590795
18	6	0	1.574606	-0.807921	1.010575
19	6	0	1.975141	-3.141666	0.473144
20	1	0	-0.661518	1.825485	2.388739
21	1	0	3.183104	1.837261	-0.039117
22	1	0	5.790833	1.243888	-0.089429
23	1	0	8.538359	-0.920137	-0.336199
24	1	0	7.950704	0.667145	-0.855801
25	1	0	7.974224	-0.690414	-1.990240
26	1	0	7.286970	-3.046343	-1.979823
27	1	0	7.801766	-3.252179	-0.307533
28	1	0	6.439544	-4.193457	-0.931872
29	1	0	4.368990	-3.487511	-0.172829
30	1	0	2.518397	-3.777619	1.177282
31	1	0	0.913042	-3.160734	0.698789
32	6	0	-2.474713	2.429856	-0.494048
33	6	0	-1.367615	3.292184	-0.502212
34	6	0	0.009209	1.674758	-1.009438
35	6	0	-2.187631	1.081288	-0.812128
36	6	0	-3.231789	4.362002	-0.033536
37	1	0	-3.873957	5.196178	0.213178
38	7	0	1.343687	1.069410	-0.937974
39	7	0	-0.094491	2.964709	-0.736190
40	7	0	-0.929317	0.743332	-1.087064
41	7	0	-1.875092	4.526124	-0.206357
42	1	0	-1.340405	5.377406	-0.114479
43	7	0	-3.630595	3.132652	-0.196276
44	8	0	-3.054524	0.091107	-0.864302
45	6	0	-4.338391	0.256735	-0.224652
46	1	0	-4.167464	0.694244	0.764429
47	1	0	-4.953598	0.941109	-0.814772
48	6	0	-4.962314	-1.105153	-0.110890
49	6	0	-4.301050	-2.106397	0.606072
50	6	0	-6.198295	-1.355057	-0.698645
51	6	0	-4.901173	-3.356531	0.717910

52	1	0	-3.336764	-1.894895	1.060273
53	6	0	-6.807553	-2.611648	-0.576547
54	1	0	-6.697024	-0.571840	-1.266345
55	6	0	-6.141233	-3.613326	0.136792
56	1	0	-4.401136	-4.146759	1.269907
57	1	0	-6.596511	-4.595932	0.231407
58	8	0	1.678587	0.214430	-1.735323
59	8	0	2.469731	2.383570	-1.000621
60	1	0	1.977050	3.146150	-0.646089
61	1	0	2.147227	-3.485321	-0.549068
62	1	0	-8.601757	-2.060977	-1.382525
63	1	0	-8.556402	-3.636292	-0.818398
64	7	0	-8.025149	-2.872724	-1.212266

The reduction of AMNBP mediated by FMNH calculated at the B3LYP/6-31+G(d,p) theoretical level

RC

0 1

Standard orientation:

Center Number	Atomic Number	Atomic Type	Coordinates (Angstroms) X	Y	Z
1	6	0	-1.795888	1.283643	0.066341
2	6	0	-3.193199	1.271423	-0.134549
3	6	0	-3.253938	-0.904040	-0.177182
4	6	0	-1.185022	0.008268	0.137756
5	6	0	-2.405378	3.329150	-0.004921
6	1	0	-2.418926	4.410559	0.002554
7	7	0	-4.041318	-2.173523	-0.318798
8	7	0	-3.978853	0.198937	-0.252477
9	7	0	-1.956754	-1.079186	-0.001835
10	7	0	-3.559440	2.593229	-0.177835
11	1	0	-4.499473	2.941140	-0.305064
12	7	0	-1.333628	2.590963	0.143846
13	8	0	0.102841	-0.254510	0.330798
14	6	0	1.057667	0.838197	0.523175
15	1	0	1.024550	1.489832	-0.352273
16	1	0	0.745970	1.414467	1.398117
17	6	0	2.417000	0.224793	0.709023
18	6	0	3.287484	0.103222	-0.378815

19	6	0	2.819401	-0.239908	1.969400
20	6	0	4.554213	-0.478379	-0.231476
21	1	0	2.993490	0.469842	-1.358194
22	6	0	4.077596	-0.818409	2.129949
23	1	0	2.148363	-0.147486	2.819400
24	6	0	4.940265	-0.932453	1.035191
25	1	0	4.392367	-1.172810	3.107171
26	1	0	5.924488	-1.375195	1.169378
27	8	0	-3.614408	-3.005191	-1.110462
28	8	0	-5.053164	-2.273331	0.367367
29	6	0	5.474809	-0.641368	-1.427551
30	1	0	6.482328	-0.899095	-1.062809
31	1	0	5.133392	-1.496549	-2.025800
32	7	0	5.441704	0.535470	-2.304380
33	1	0	5.863329	1.340881	-1.848556
34	1	0	5.955925	0.362600	-3.163241

IC$_1$

0 2

Standard orientation:

Center Number	Atomic Number	Atomic Type	Coordinates (Angstroms) X	Y	Z
1	6	0	-1.759865	1.327161	0.111723
2	6	0	-3.157512	1.335677	-0.094107
3	6	0	-3.265307	-0.851724	-0.183469
4	6	0	-1.174281	0.037655	0.154100
5	6	0	-2.341193	3.382947	0.094155
6	1	0	-2.340347	4.463825	0.129881
7	7	0	-4.037442	-2.018868	-0.336025
8	7	0	-3.962041	0.289528	-0.247908
9	7	0	-1.953307	-1.032397	0.003687
10	7	0	-3.504834	2.665997	-0.101184
11	1	0	-4.439105	3.028306	-0.227307
12	7	0	-1.280138	2.626271	0.225549
13	8	0	0.118413	-0.245109	0.332819
14	6	0	1.081931	0.833498	0.525067
15	1	0	1.049845	1.496946	-0.341937
16	1	0	0.789365	1.406967	1.408663
17	6	0	2.438798	0.205967	0.690994
18	6	0	3.309247	0.111286	-0.399436

19	6	0	2.839760	-0.297480	1.936940
20	6	0	4.573765	-0.479763	-0.268991
21	1	0	3.016311	0.507383	-1.367587
22	6	0	4.095194	-0.886219	2.081005
23	1	0	2.168612	-0.226720	2.788912
24	6	0	4.958076	-0.972087	0.983728
25	1	0	4.408059	-1.270149	3.047741
26	1	0	5.940572	-1.422490	1.105026
27	8	0	-3.323694	-3.222520	-0.265016
28	8	0	-5.271757	-2.070886	-0.519863
29	1	0	-4.040116	-3.870095	-0.395354
30	6	0	5.494612	-0.611411	-1.468584
31	1	0	6.498870	-0.891379	-1.110960
32	1	0	5.146301	-1.441921	-2.097021
33	7	0	5.475600	0.595200	-2.304858
34	1	0	5.892037	1.383328	-1.815262
35	1	0	6.001658	0.450985	-3.161897

IC$_2$

0 1

Standard orientation:

Center Number	Atomic Number	Atomic Type	Coordinates (Angstroms) X	Y	Z
1	6	0	-2.055838	0.988503	0.111742
2	6	0	-3.451068	0.893154	-0.106177
3	6	0	-3.383073	-1.292336	-0.259030
4	6	0	-1.365873	-0.247443	0.122843
5	6	0	-2.800522	2.989585	0.147138
6	1	0	-2.882821	4.066071	0.214021
7	7	0	-3.989802	-2.628566	-0.449927
8	7	0	-4.162102	-0.211542	-0.295742
9	7	0	-2.065081	-1.371017	-0.066733
10	7	0	-3.901301	2.193466	-0.078155
11	1	0	-4.860051	2.486842	-0.202155
12	7	0	-1.681897	2.315597	0.266622
13	8	0	-0.058674	-0.433455	0.304602
14	6	0	0.821670	0.709543	0.534357
15	1	0	0.745633	1.389421	-0.317174
16	1	0	0.479036	1.235507	1.429248
17	6	0	2.219093	0.179162	0.697502

18	6	0	3.087919	0.136091	-0.397569
19	6	0	2.658299	-0.289322	1.944050
20	6	0	4.388288	-0.371557	-0.271280
21	1	0	2.764780	0.506800	-1.366183
22	6	0	3.950285	-0.793912	2.084161
23	1	0	1.988551	-0.257789	2.799505
24	6	0	4.810310	-0.830753	0.982035
25	1	0	4.292777	-1.151020	3.051064
26	1	0	5.820226	-1.216720	1.099776
27	8	0	-5.188195	-2.602494	-0.622311
28	6	0	5.308007	-0.452165	-1.476208
29	1	0	5.008097	-1.306894	-2.096981
30	1	0	6.330170	-0.666400	-1.124220
31	7	0	5.207378	0.745532	-2.319374
32	1	0	5.583984	1.559952	-1.840670
33	1	0	5.729225	0.626235	-3.182778

TS_1

0 2

Standard orientation:

Center Number	Atomic Number	Atomic Type	Coordinates (Angstroms) X	Y	Z
1	6	0	-3.095799	-0.901961	2.108930
2	6	0	-1.831418	-0.885236	2.738403
3	6	0	-0.896169	0.178299	1.070242
4	6	0	-3.133932	-0.272371	0.838557
5	6	0	-3.352540	-1.903082	3.978220
6	1	0	-3.767415	-2.435790	4.823081
7	7	0	0.258852	0.767340	0.449936
8	7	0	-0.702947	-0.359769	2.275598
9	7	0	-2.016108	0.258075	0.349519
10	7	0	-2.025364	-1.532228	3.937244
11	1	0	-1.313765	-1.707437	4.632130
12	7	0	-4.026648	-1.546202	2.911579
13	8	0	-4.201436	-0.139588	0.047443
14	6	0	-5.488586	-0.689327	0.458607
15	1	0	-5.373843	-1.764628	0.617662
16	1	0	-5.783678	-0.230234	1.404940
17	6	0	-6.478325	-0.389642	-0.633537
18	6	0	-6.584366	-1.236449	-1.745236

19	6	0	-7.292801	0.745420	-0.554164
20	6	0	-7.488582	-0.939765	-2.764866
21	1	0	-5.952592	-2.118047	-1.815412
22	6	0	-8.207499	1.053399	-1.569999
23	1	0	-7.216153	1.413761	0.298646
24	6	0	-8.291419	0.201060	-2.678225
25	1	0	-7.564581	-1.591340	-3.630616
26	1	0	-8.988628	0.430905	-3.480845
27	8	0	0.221263	1.234615	-0.694991
28	8	0	1.364513	0.793768	1.165776
29	7	0	5.266898	-2.817505	-1.229205
30	6	0	4.289230	-3.788029	-1.254012
31	8	0	4.493739	-4.949097	-1.578559
32	7	0	2.979358	-3.406679	-0.877885
33	6	0	2.559184	-2.155929	-0.476002
34	8	0	1.404434	-1.915057	-0.137824
35	6	0	3.643560	-1.154136	-0.478401
36	7	0	3.369637	0.098818	-0.129044
37	6	0	4.370409	1.039187	-0.118339
38	6	0	4.080502	2.370543	0.243946
39	6	0	5.063086	3.349735	0.264950
40	6	0	4.717393	4.765670	0.657771
41	6	0	6.391067	2.993818	-0.098233
42	6	0	7.489160	4.027699	-0.095068
43	6	0	6.684278	1.679101	-0.466203
44	6	0	5.695670	0.683065	-0.484020
45	7	0	5.967060	-0.633795	-0.846006
46	6	0	4.962623	-1.593692	-0.864780
47	6	0	7.329622	-1.020082	-1.225839
48	1	0	2.280440	-4.140781	-0.899176
49	1	0	2.265994	0.432884	0.475332
50	1	0	3.055903	2.611470	0.506669
51	1	0	5.295367	5.095615	1.529370
52	1	0	3.657263	4.853082	0.906573
53	1	0	4.930898	5.472674	-0.152898
54	1	0	7.262453	4.853797	-0.779819
55	1	0	7.618647	4.470087	0.900020
56	1	0	8.445487	3.593708	-0.397244
57	1	0	7.702618	1.433697	-0.740474
58	1	0	8.015918	-0.827874	-0.395692
59	1	0	7.320744	-2.081206	-1.461182
60	1	0	7.648971	-0.448903	-2.102885

61	6	0	-9.114703	2.266473	-1.462607
62	1	0	-9.991523	2.005507	-0.854341
63	1	0	-9.495835	2.506368	-2.468820
64	7	0	-8.449587	3.390724	-0.794979
65	1	0	-7.680123	3.745040	-1.357868
66	1	0	-9.098577	4.156460	-0.639783

TS₂

0 1

Standard orientation:

Center Number	Atomic Number	Atomic Type	Coordinates (Angstroms) X	Y	Z
1	7	0	6.968725	-0.461696	0.587065
2	6	0	7.461998	0.823344	0.616544
3	8	0	8.610569	1.106569	0.922574
4	7	0	6.575217	1.876969	0.268024
5	6	0	5.258553	1.758345	-0.110562
6	8	0	4.549257	2.720008	-0.410951
7	6	0	4.799459	0.364884	-0.127381
8	7	0	3.534784	0.093321	-0.465611
9	6	0	3.056088	-1.185115	-0.526760
10	6	0	1.731079	-1.443474	-0.932776
11	6	0	1.230530	-2.739356	-0.955892
12	6	0	-0.198851	-2.987021	-1.370063
13	6	0	2.082545	-3.808646	-0.578476
14	6	0	1.577681	-5.229227	-0.594220
15	6	0	3.406120	-3.555991	-0.195431
16	6	0	3.920067	-2.255642	-0.159234
17	7	0	5.229416	-1.972585	0.228851
18	6	0	5.719618	-0.671549	0.238722
19	6	0	6.117168	-3.067910	0.631135
20	1	0	6.965339	2.812395	0.299777
21	1	0	2.850360	0.903441	-0.643158
22	1	0	1.109451	-0.609176	-1.227141
23	1	0	-0.260007	-3.643793	-2.246389
24	1	0	-0.701789	-2.048269	-1.610794
25	1	0	-0.768541	-3.467231	-0.565840
26	1	0	0.723180	-5.352940	0.081857
27	1	0	1.232431	-5.519361	-1.593772
28	1	0	2.355249	-5.933375	-0.288175

29	1	0	4.033599	-4.394024	0.079412
30	1	0	6.243332	-3.769526	-0.199104
31	1	0	7.078505	-2.636986	0.898494
32	6	0	-2.976909	3.024095	-0.186371
33	6	0	-1.819055	3.806769	-0.397825
34	6	0	-0.471742	2.174507	0.200576
35	6	0	-2.723724	1.709328	0.263942
36	6	0	-3.669241	4.932176	-0.854638
37	1	0	-4.287689	5.765144	-1.160549
38	7	0	0.870694	1.743217	0.536353
39	7	0	-0.556215	3.444305	-0.211899
40	7	0	-1.461262	1.314193	0.443140
41	7	0	-2.292169	5.026048	-0.827092
42	1	0	-1.724502	5.825752	-1.067559
43	7	0	-4.119809	3.760897	-0.481861
44	8	0	-3.635997	0.766782	0.531075
45	6	0	-5.052589	1.075932	0.417176
46	1	0	-5.273860	1.928630	1.066449
47	1	0	-5.280543	1.365723	-0.611093
48	6	0	-5.826184	-0.149652	0.825965
49	6	0	-5.726285	-0.648617	2.133694
50	6	0	-6.666401	-0.789519	-0.089660
51	6	0	-6.454365	-1.776665	2.506147
52	1	0	-5.067234	-0.162549	2.847526
53	6	0	-7.414881	-1.918382	0.277009
54	1	0	-6.743813	-0.423245	-1.109396
55	6	0	-7.293551	-2.408532	1.581569
56	1	0	-6.368142	-2.169328	3.515321
57	1	0	-7.856048	-3.290668	1.878970
58	8	0	1.048134	0.567479	0.941122
59	8	0	1.717375	1.980123	-0.825550
60	1	0	2.178909	2.818835	-0.651574
61	1	0	5.690714	-3.593022	1.490972
62	6	0	-8.358709	-2.578219	-0.712109
63	1	0	-9.286286	-1.992578	-0.768190
64	1	0	-8.640121	-3.569082	-0.319659
65	7	0	-7.793630	-2.613577	-2.066582
66	1	0	-6.982569	-3.225888	-2.106144
67	1	0	-8.475403	-2.959387	-2.735601

The reduction of AMNBP mediated by FMNH calculated at the M062X/6-31+G(d,p) theoretical level

RC

0 1

Standard orientation:

Center Number	Atomic Number	Atomic Type	Coordinates (Angstroms) X	Y	Z
1	6	0	1.786736	1.271920	0.123998
2	6	0	3.173408	1.166416	0.327578
3	6	0	3.153947	-0.971263	-0.021446
4	6	0	1.133944	0.054558	-0.178559
5	6	0	2.472645	3.236207	0.560312
6	1	0	2.529343	4.300030	0.745790
7	7	0	3.893076	-2.278480	-0.107715
8	7	0	3.915037	0.061748	0.259157
9	7	0	1.857968	-1.063543	-0.236564
10	7	0	3.590131	2.437984	0.606874
11	1	0	4.540793	2.716236	0.803218
12	7	0	1.378346	2.586460	0.276267
13	8	0	-0.155538	-0.106311	-0.418785
14	6	0	-1.030374	1.043906	-0.363383
15	1	0	-0.947255	1.498560	0.627799
16	1	0	-0.702249	1.774643	-1.108196
17	6	0	-2.422603	0.553562	-0.635160
18	6	0	-3.162623	-0.030999	0.392977
19	6	0	-2.971614	0.651093	-1.914601
20	6	0	-4.445264	-0.527147	0.157092
21	1	0	-2.748404	-0.107306	1.395292
22	6	0	-4.254223	0.167480	-2.158793
23	1	0	-2.396466	1.107306	-2.716134
24	6	0	-4.985425	-0.416164	-1.126416
25	1	0	-4.686792	0.250827	-3.150892
26	1	0	-5.989940	-0.786435	-1.317740
27	8	0	3.295067	-3.270109	0.248355
28	8	0	5.032745	-2.231269	-0.522689
29	6	0	-5.224781	-1.200409	1.266664
30	1	0	-6.286986	-1.232317	0.979730
31	1	0	-4.888629	-2.240986	1.349551
32	7	0	-4.974493	-0.558078	2.554492
33	1	0	-5.377567	0.373984	2.574836
34	1	0	-5.387136	-1.090611	3.312551

IC$_1$

0 2

Standard orientation:

Center Number	Atomic Number	Atomic Type	Coordinates (Angstroms) X	Y	Z
1	6	0	1.757665	1.322945	0.116451
2	6	0	3.144517	1.217892	0.320581
3	6	0	3.147226	-0.932044	-0.041285
4	6	0	1.115578	0.101119	-0.192310
5	6	0	2.436331	3.289908	0.557463
6	1	0	2.490615	4.353121	0.746596
7	7	0	3.856693	-2.143663	-0.132803
8	7	0	3.895317	0.124522	0.256689
9	7	0	1.835651	-1.010467	-0.265559
10	7	0	3.556614	2.492539	0.601880
11	1	0	4.505630	2.770211	0.804247
12	7	0	1.344944	2.637735	0.271741
13	8	0	-0.180622	-0.062382	-0.427139
14	6	0	-1.053628	1.083020	-0.348105
15	1	0	-0.961868	1.528596	0.646777
16	1	0	-0.739616	1.826112	-1.086934
17	6	0	-2.449597	0.594139	-0.608682
18	6	0	-3.153429	-0.059500	0.403849
19	6	0	-3.040770	0.764561	-1.861452
20	6	0	-4.439216	-0.551841	0.178140
21	1	0	-2.705935	-0.192873	1.385605
22	6	0	-4.327663	0.285948	-2.094820
23	1	0	-2.494535	1.273491	-2.651269
24	6	0	-5.021812	-0.366674	-1.078437
25	1	0	-4.791710	0.425716	-3.066204
26	1	0	-6.029324	-0.733516	-1.260457
27	8	0	3.095759	-3.258984	-0.406559
28	8	0	5.076718	-2.285637	0.047743
29	1	0	3.769695	-3.958336	-0.416247
30	6	0	-5.179999	-1.298184	1.267324
31	1	0	-6.249099	-1.327991	1.006264
32	1	0	-4.829606	-2.337280	1.282550
33	7	0	-4.903896	-0.726895	2.583023
34	1	0	-5.303622	0.203595	2.660895
35	1	0	-5.303932	-1.298943	3.318698

IC_2

0 1

Standard orientation:

Center Number	Atomic Number	Atomic Type	Coordinates (Angstroms) X	Y	Z
1	6	0	2.018874	0.974966	-0.133858
2	6	0	3.405022	0.899504	0.101678
3	6	0	3.364642	-1.273169	0.276936
4	6	0	1.349452	-0.269270	-0.135860
5	6	0	2.736403	2.976051	-0.187078
6	1	0	2.808888	4.052137	-0.267757
7	7	0	3.995666	-2.600926	0.491053
8	7	0	4.127771	-0.193495	0.312123
9	7	0	2.055947	-1.377553	0.073318
10	7	0	3.839753	2.198267	0.063548
11	1	0	4.793091	2.502709	0.195952
12	7	0	1.632159	2.293203	-0.311843
13	8	0	0.051913	-0.462472	-0.328411
14	6	0	-0.800401	0.680577	-0.551429
15	1	0	-0.712868	1.364548	0.297288
16	1	0	-0.461418	1.202143	-1.451753
17	6	0	-2.202512	0.164157	-0.701722
18	6	0	-3.066011	0.152521	0.392351
19	6	0	-2.637087	-0.334989	-1.932195
20	6	0	-4.362339	-0.355972	0.275975
21	1	0	-2.742267	0.543659	1.353579
22	6	0	-3.926886	-0.839819	-2.060219
23	1	0	-1.963284	-0.327392	-2.784991
24	6	0	-4.784924	-0.845343	-0.960729
25	1	0	-4.269583	-1.221871	-3.016736
26	1	0	-5.795659	-1.232290	-1.066873
27	8	0	5.180735	-2.538938	0.640523
28	6	0	-5.279549	-0.404983	1.479207
29	1	0	-5.033958	-1.294514	2.071700
30	1	0	-6.314044	-0.535153	1.126051
31	7	0	-5.086174	0.758215	2.341577
32	1	0	-5.400592	1.605147	1.877267
33	1	0	-5.613456	0.662791	3.202607

TS$_1$

0 2

Standard orientation:

Center Number	Atomic Number	Atomic Type	Coordinates (Angstroms) X	Y	Z
1	6	0	3.033615	-2.845919	0.625773
2	6	0	1.824518	-3.504496	0.910714
3	6	0	0.628431	-1.926601	0.009470
4	6	0	2.884003	-1.608292	-0.044847
5	6	0	3.566264	-4.652077	1.612972
6	1	0	4.115269	-5.461112	2.074826
7	7	0	-0.652259	-1.373248	-0.345571
8	7	0	0.593869	-3.096957	0.625909
9	7	0	1.668258	-1.176978	-0.343292
10	7	0	2.193986	-4.660876	1.544420
11	1	0	1.561401	-5.363989	1.896725
12	7	0	4.108203	-3.593130	1.077330
13	8	0	3.869524	-0.803978	-0.427250
14	6	0	5.223007	-1.158528	-0.109820
15	1	0	5.303500	-1.314422	0.971689
16	1	0	5.483392	-2.100010	-0.603140
17	6	0	6.119335	-0.038449	-0.564637
18	6	0	7.393840	-0.327749	-1.049520
19	6	0	5.706795	1.292074	-0.467956
20	6	0	8.248796	0.708077	-1.424188
21	1	0	7.715978	-1.361883	-1.143544
22	6	0	6.556031	2.333655	-0.841236
23	1	0	4.701311	1.511089	-0.116460
24	6	0	7.834033	2.032793	-1.322333
25	1	0	9.238810	0.478630	-1.806834
26	1	0	8.483393	2.848199	-1.625273
27	8	0	-0.747513	-0.288763	-0.907503
28	8	0	-1.696626	-2.069290	-0.024995
29	7	0	-3.134784	3.235476	1.428956
30	6	0	-1.904922	3.271140	2.058342
31	8	0	-1.424917	4.283044	2.524936
32	7	0	-1.192007	2.062651	2.170541
33	6	0	-1.581196	0.825577	1.713517
34	8	0	-0.962562	-0.200513	1.953640
35	6	0	-2.843440	0.862584	0.954077

36	7	0	-3.296182	-0.233606	0.378028
37	6	0	-4.515305	-0.239611	-0.250808
38	6	0	-4.976371	-1.411688	-0.870615
39	6	0	-6.204433	-1.452150	-1.505327
40	6	0	-6.684506	-2.716326	-2.167733
41	6	0	-7.000720	-0.280353	-1.526926
42	6	0	-8.340818	-0.293566	-2.211661
43	6	0	-6.542932	0.886272	-0.923350
44	6	0	-5.298589	0.932166	-0.281169
45	7	0	-4.805872	2.090232	0.310939
46	6	0	-3.572896	2.115499	0.927845
47	6	0	-5.600721	3.314101	0.258240
48	1	0	-0.322463	2.108327	2.688475
49	1	0	-2.535490	-1.213731	0.238993
50	1	0	-4.336278	-2.289659	-0.842278
51	1	0	-7.624088	-3.065827	-1.726490
52	1	0	-5.945226	-3.512748	-2.064517
53	1	0	-6.867320	-2.559445	-3.236106
54	1	0	-8.237411	-0.535628	-3.275010
55	1	0	-9.000918	-1.049627	-1.773279
56	1	0	-8.835011	0.676573	-2.131018
57	1	0	-7.168003	1.770692	-0.958271
58	1	0	-6.560589	3.150205	0.755480
59	1	0	-5.046104	4.096738	0.768280
60	1	0	-5.770709	3.597707	-0.784152
61	1	0	5.005808	3.786826	-0.624992
62	6	0	6.103499	3.771234	-0.698087
63	7	0	6.640449	4.605917	-1.771137
64	1	0	6.239382	4.335188	-2.664511
65	1	0	6.411627	5.581731	-1.614938
66	1	0	6.488941	4.169622	0.248436

TS_2

0 1

Standard orientation:

Center Number	Atomic Number	Atomic Type	Coordinates (Angstroms) X	Y	Z
1	7	0	0.582871	-1.026205	1.443788
2	6	0	-0.229797	0.001521	1.849254
3	8	0	-1.411307	-0.125854	2.107925

4	7	0	0.341286	1.288884	2.011948
5	6	0	1.595958	1.673442	1.625667
6	8	0	2.036273	2.795371	1.775064
7	6	0	2.347664	0.585806	0.918830
8	7	0	3.618202	0.827878	0.604969
9	6	0	4.416917	-0.231637	0.190679
10	6	0	5.722677	0.014672	-0.240722
11	6	0	6.561267	-1.015527	-0.641989
12	6	0	7.961626	-0.723395	-1.112910
13	6	0	6.082321	-2.342205	-0.596874
14	6	0	6.970181	-3.485185	-1.014572
15	6	0	4.782676	-2.590868	-0.166321
16	6	0	3.934943	-1.548368	0.222205
17	7	0	2.608828	-1.780213	0.624481
18	6	0	1.799830	-0.776333	1.038632
19	6	0	2.080487	-3.142558	0.572606
20	1	0	-0.262443	1.993427	2.420847
21	1	0	3.485342	1.760609	-0.150998
22	1	0	6.067032	1.045111	-0.253416
23	1	0	8.705659	-1.231291	-0.489849
24	1	0	8.168184	0.348132	-1.081154
25	1	0	8.113280	-1.066967	-2.141789
26	1	0	7.292352	-3.376507	-2.055793
27	1	0	7.876180	-3.529747	-0.400553
28	1	0	6.451287	-4.441406	-0.920501
29	1	0	4.431355	-3.615770	-0.144253
30	1	0	2.621120	-3.776953	1.279879
31	1	0	1.027433	-3.107808	0.835030
32	6	0	-2.160781	2.582309	-0.454136
33	6	0	-1.017382	3.393719	-0.518321
34	6	0	0.273344	1.699817	-1.000565
35	6	0	-1.941167	1.211877	-0.728690
36	6	0	-2.821594	4.561414	-0.049466
37	1	0	-3.421597	5.431349	0.178821
38	7	0	1.583799	1.039176	-0.951853
39	7	0	0.233967	3.001321	-0.770108
40	7	0	-0.705873	0.808184	-1.020203
41	7	0	-1.463365	4.658797	-0.257082
42	1	0	-0.890624	5.488827	-0.210189
43	7	0	-3.277413	3.345898	-0.157857
44	8	0	-2.852371	0.260597	-0.722943
45	6	0	-4.104392	0.500400	-0.041208

46	1	0	-3.877108	0.980686	0.916085
47	1	0	-4.722931	1.173159	-0.640371
48	6	0	-4.762603	-0.833953	0.165027
49	6	0	-4.106539	-1.811606	0.921417
50	6	0	-6.017967	-1.095194	-0.378386
51	6	0	-4.717807	-3.044193	1.120058
52	1	0	-3.125417	-1.595272	1.337864
53	6	0	-6.641571	-2.330645	-0.173062
54	1	0	-6.528200	-0.346270	-0.978709
55	6	0	-5.976783	-3.302522	0.573618
56	1	0	-4.212442	-3.811130	1.698991
57	1	0	-6.445011	-4.272126	0.727662
58	8	0	1.852179	0.147180	-1.732642
59	8	0	2.762853	2.305803	-1.100559
60	1	0	2.314244	3.098466	-0.753896
61	1	0	2.200954	-3.528598	-0.441729
62	6	0	-8.021346	-2.599422	-0.734754
63	1	0	-8.772005	-2.179629	-0.053851
64	1	0	-8.183923	-3.688115	-0.753072
65	7	0	-8.201336	-1.949827	-2.031663
66	1	0	-7.571622	-2.350304	-2.721144
67	1	0	-9.149847	-2.072098	-2.369714
